# Design and property investigation on a five-interaction-based fluorescent anion receptor clip†

**DOI:** 10.1039/d1ra00630d

**Published:** 2021-03-03

**Authors:** Romain Plais, Hamza Boufroura, Guy Gouarin, Anne Gaucher, Violette Haldys, Arnaud Brosseau, Gilles Clavier, Jean-Yves Salpin, Damien Prim

**Affiliations:** Université Paris-Saclay, UVSQ, CNRS, Institut Lavoisier de Versailles 78035 Versailles France damien.prim@uvsq.fr; Université Paris-Saclay, Univ Evry, CNRS, LAMBE 91025 Evry-Courcouronnes France; LAMBE, CY Paris Cergy Université, CNRS 95000 Cergy France; Université Paris-Saclay, ENS Paris-Saclay, CNRS, PPSM 91190 Gif-sur-Yvette France

## Abstract

A molecular clip combining a doubly substituted fluorescent anion–π donor probe and two flexible arms bearing H-bond motifs constitutes a new generation of anion receptors. Five simultaneous non-covalent interactions are highlighted by theoretical complexation studies with five different anions. A large range of analytical techniques (electrospray-tandem mass spectrometry, NMR, UV-visible, steady-state and time-resolved fluorescence) were deployed to evaluate the stoichiometry and association constants with the selected anions. The photophysical and anion–π donor properties of the tetrazine ring allowed fine characterization of the binding properties of the ligand. Based on previously published results, an anti-cooperativity effect in non-covalent interactions was demonstrated.

## Abstract 2

A new generation of anion acceptor is designed from three key structural fragments and five simultaneous non-covalent interactions.

## Graphical abstract

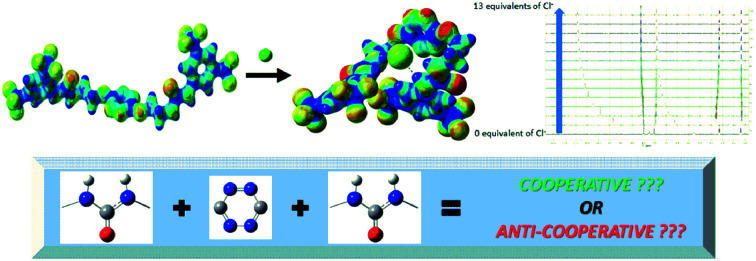

## Introduction

Non-covalent interactions have been widely used in the elaboration of new molecular organic architectures. Their use in molecular recognition is now established as a milestone in supramolecular chemistry, defined by Jean-Marie Lehn as the “chemistry beyond the molecule”.^1^ The hydrogen and halogen bonds, which were recently recognized by the IUPAC,^2,3^ have been reported independently in the binding of ions. More recently, subtle interactions such as cation–π^4^ or anion–π^5,6^ have emerged in the field. However, anion binding is more difficult to achieve than cation binding due to the high solvation energy and more diffuse charges of the former.^7^

Thus, the combination of non-covalent interactions is a promising strategy to bind anions, but also to construct supramolecular assemblies like folding structures or polymers.^8–10^ We recently turned our attention to a mix of hydrogen bonding and anion–π interaction. Such combination has been studied theoretically by Liu and coworkers,^11–14^ showing interesting cooperative effects between interactions. Despite these interesting reports, few studies in solution have been published to date.^15–18^

Following our interest in non-covalent interactions,^19,20^ we focused on the tetrazine ring, which exhibits both excellent photophysical^21–24^ and anion–π binding properties.^25,26^ We recently showed that photophysical properties are particularly useful to characterize anion complexes in solution in a new anion receptor (1) combining a tetrazine and an urea subunits linked by a short flexible arm.^27^

**Scheme 1:**
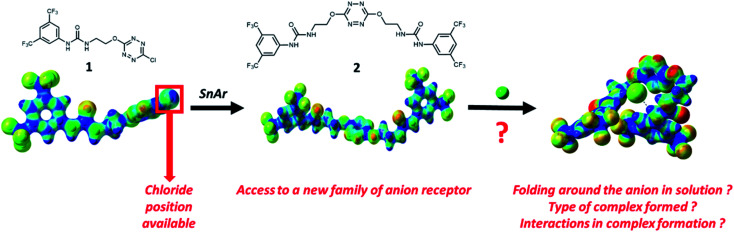
Design strategy.

Examination of the receptor 1 shows that an extra position is available at the tetrazine core for installing an additional urea motif (Scheme 1). We hypothesized that a second nucleophilic aromatic substitution (S_N_Ar) would enable access to a new family of anion receptors. In this context, the following questions arose from this design: will folding really occur in solution? What type of complex will be formed? Which interactions will help maintaining this complex? To address these different points, we report the synthesis and exploration of anion binding properties of a double-armed anion receptor, comprising a theoretical approach and a broad scope of experimental methods, namely electrospray-tandem mass spectrometry, NMR, UV-visible and steady state fluorescence as well as time-resolved fluorescence.

## Results and discussion

### Molecular design and quantum descriptors

Prior to the examination of the complexation properties of the new ligand synthesized, we evaluated the binding and conformational preferences of our new ligand with five different anions through density functional theory (DFT) calculation using Gaussian 09 software. In order to take into account the dispersion effects essential in the description of non-covalent interactions, the Austin-Frisch-Petersson functional (APFD) was selected.^28^ 1 : 1 host–guest complexes were obtained through geometry optimization and frequencies calculations at the APFD/6-31+G(d,p) level of theory without any solvent effect. For complexes including heavy atoms, a split basis set was applied, composed of APFD/aug-cc-pvtz for bromide or iodide anions, and APFD/6-31+G(d,p) for other atoms. Geometry optimization and frequencies calculations were also performed using Polarizable Continuum Model (PCM) for acetonitrile. Only a small influence on geometries was observed by application of the solvent model. Optimized geometries are detailed in the ESI (ESI pp.7–20).† Illustrations of the electronic and binding properties of the complexes were assessed by calculating the Electrostatic Potentials Surfaces (ESP) using the Gaussview software from optimized structures using a fine grid for total density and a medium grid for ESP. We used Non-Covalent Interaction plot (NCIplot) to visualize non-covalent interaction into real space, calculated using the script developed by Rzepa available on the website application of the Imperial College London.^29^ All the results of these calculations are showed in Table 1.

**Table 1:** Optimized geometries, ESP and NCIplot of receptor 2 and complexes with anions at the APFD/6-31+G(d,p) level

Entry		Optimized Geometry	ESP	NCIplot
1	2	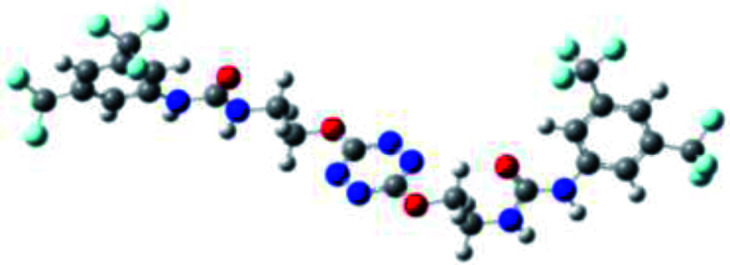	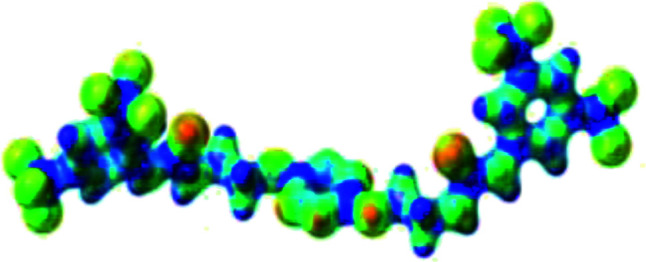	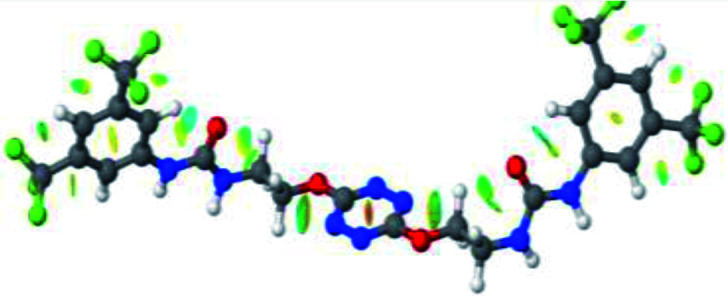
2	2–Cl	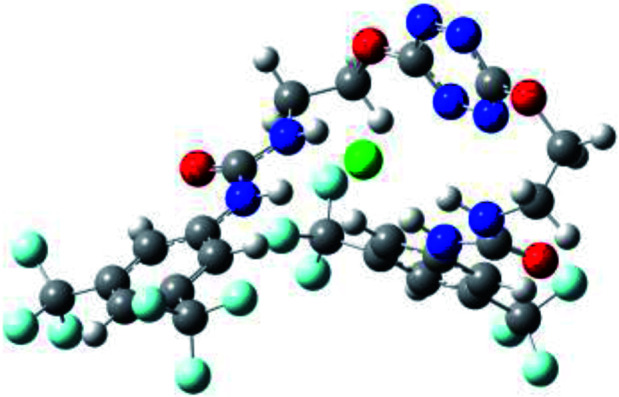	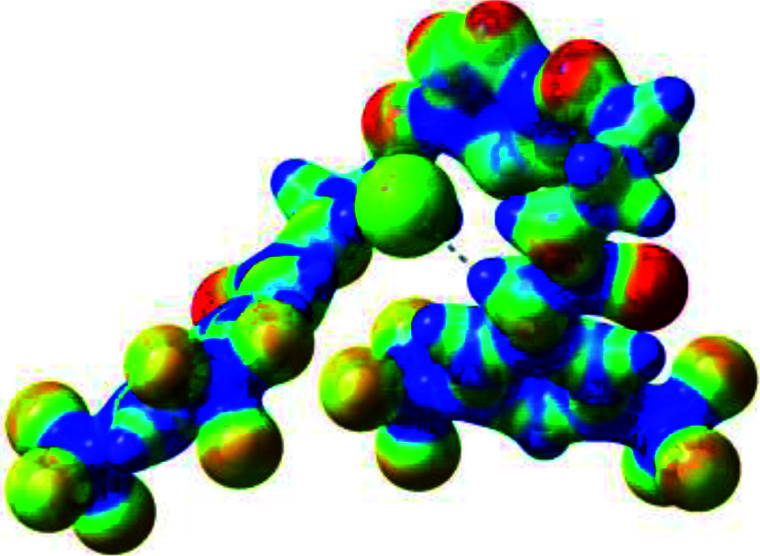	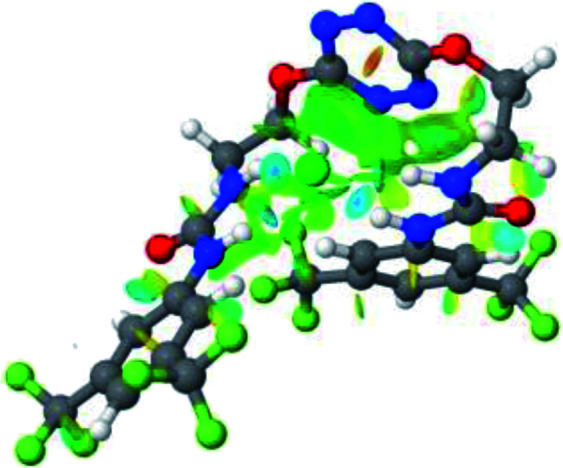
3	2–Br	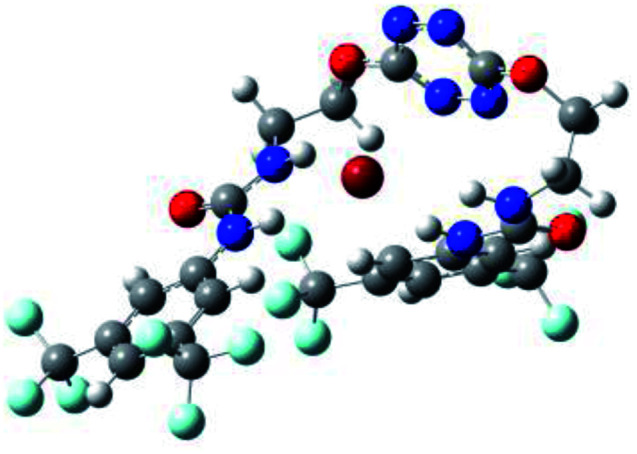	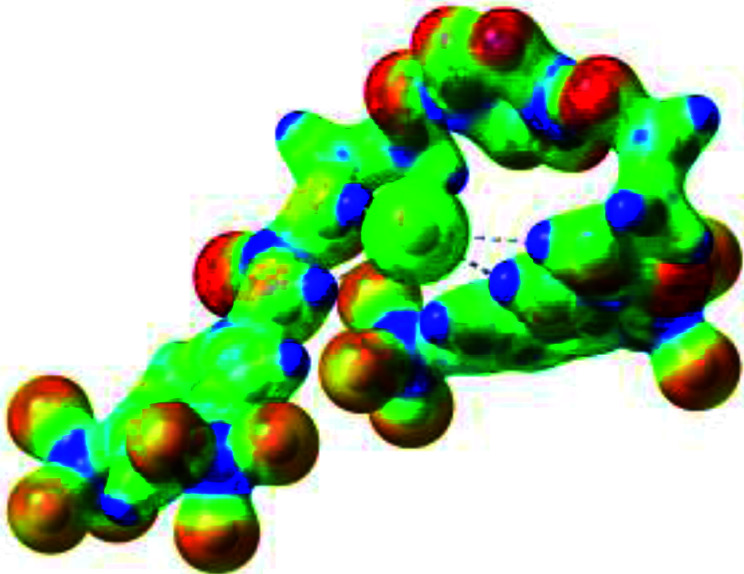	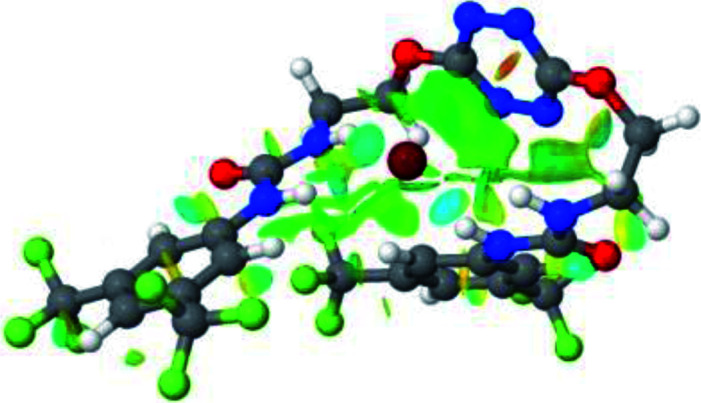
4	2–I	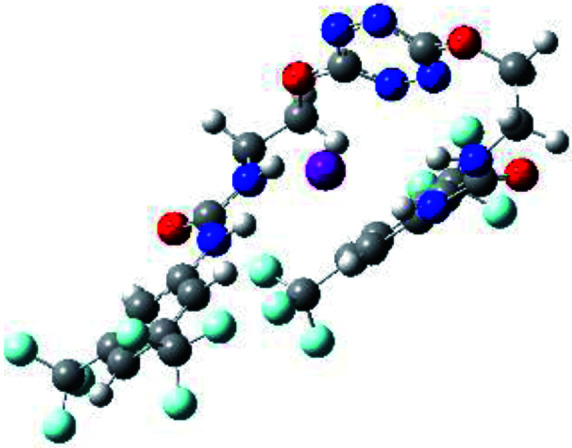	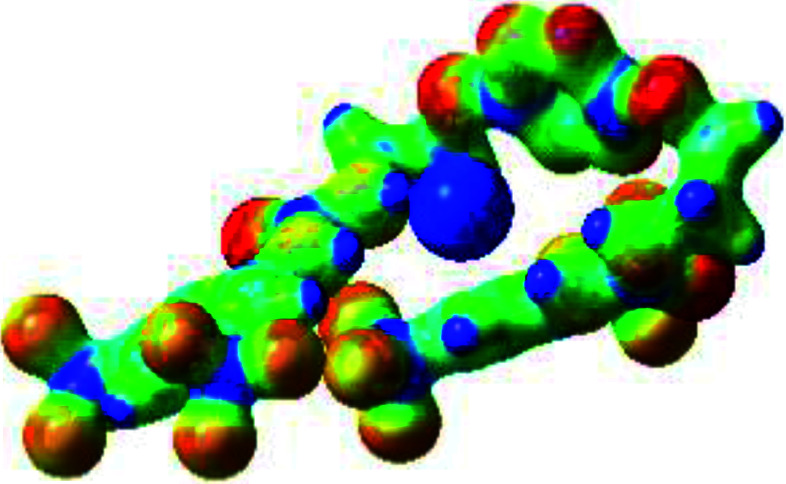	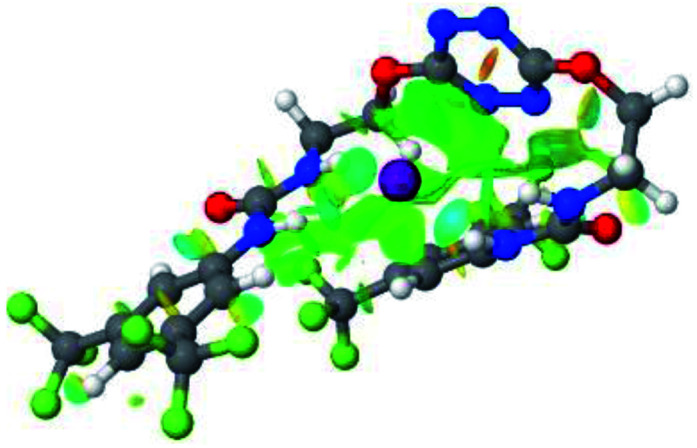
5	2–SCN	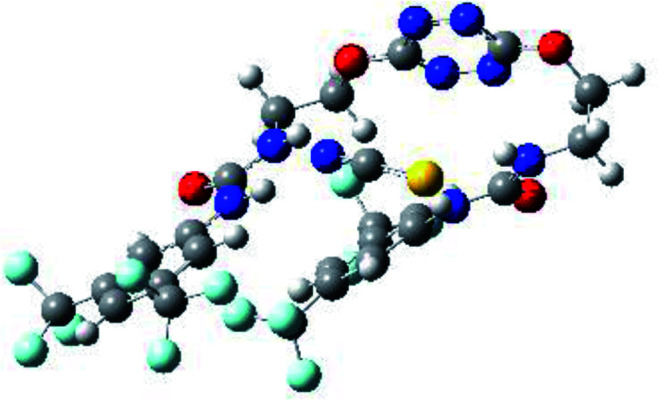	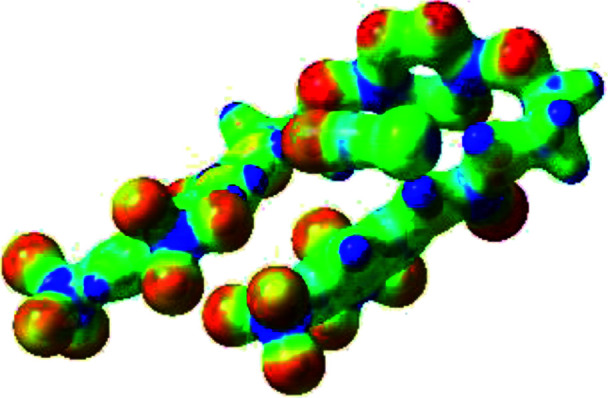	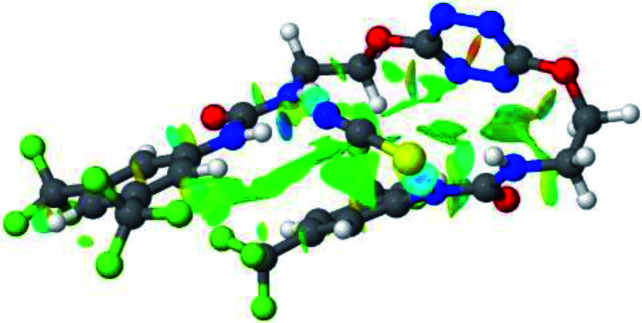
6	2–PF_6_	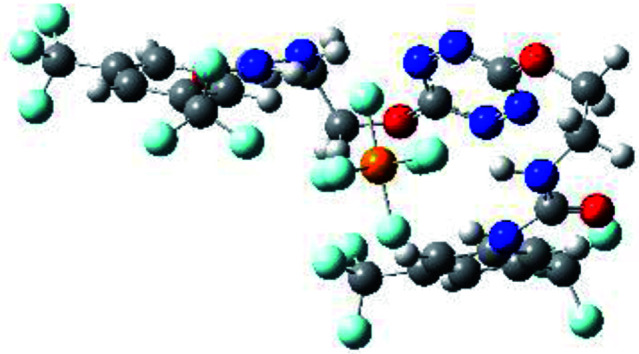	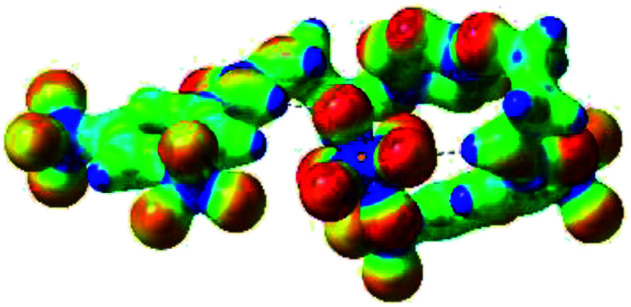	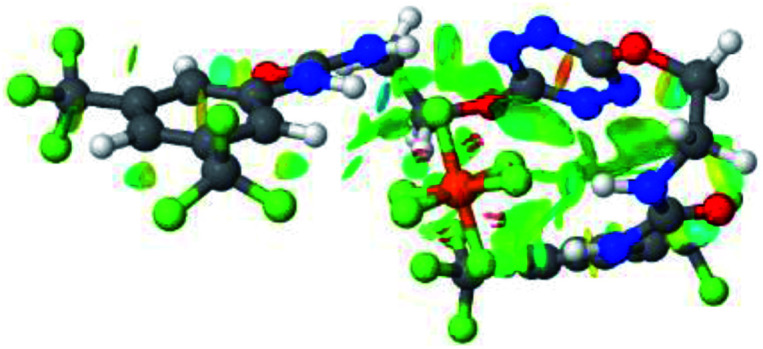

The receptor 2 adopts a linear conformation in which each arm is almost orthogonal to the tetrazine core. The linear conformation might be partly due to (i) the minimization of repulsive interactions between the tetrazine and each of the two arms, and (ii) the *anti* conformation of both N and O atoms with respect to the linear CH_2_–CH_2_ fragment (Table 1, entry 1, NCIplot). The receptor thus acquires a Z-shaped conformation which overall geometry is set by interactions including: (i) protons of the aliphatic spacer with nitrogen atoms of the tetrazine and oxygen atom of the ureas, respectively, (ii) *ortho* protons of the terminal aromatic ring with the oxygen atom of the urea, favoring a linear conformation between urea and aromatic ring. Different regions showing a deficit of electron density (in blue) are visible around the center of the tetrazine ring, on the N–H hydrogen bond donors and the aromatic substituent of the urea moiety, thus highlighting possible binding sites for anions (Table 1, entry 1, ESP).

Optimized geometries of 2–anion complexes are characterized by a marked folding of the receptor that wraps around the different anions (Table 1, optimized geometries). This folding tends to maximize attractive interactions between anions and both ureas and tetrazine moieties (Table 1, NCIplots). Large green dish-shaped surfaces between tetrazine and Cl, Br and I anions are observed, significant of anion–π interactions. Interestingly, in the case of SCN anion, the NCI plot did not evidence any surface representative of a weak interaction between the tetrazine and the anion. In contrast, the overall shape of complex 2–PF_6_ is only set by a weak anion–π interaction with hexafluorophosphate in which only one of the fluorine atoms of the anion is involved.

Moreover, in all the complexes studied, four small blue disk-shaped surfaces indicate the joint formation of four hydrogen bonds between ureas and anions.

**Fig. 1:**
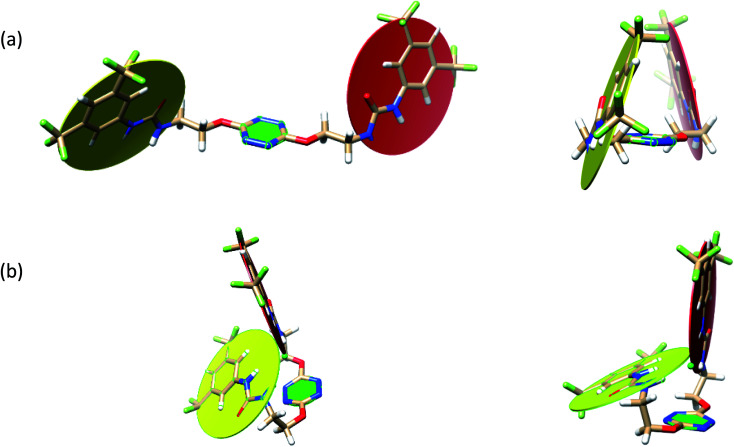
Views of the orientation of urea arms respectively to each other in (a) the receptor alone, (b) the chloride complex.

To characterize the folding process, angles between key structural fragments were measured using Chimera Software.^30^ As shown in Table 2, conformation of the complex is deeply impacted by the anion geometry. In fact, the receptor can be defined by three main planes connected together by O–CH_2_–CH_2_ flexible units: two planes A and B composed by aryl-urea fragments of each arms and one plane corresponding to the tetrazine heterocycle. In the receptor 2, the two urea arms are U-shaped with an angle of 31° (Table 2, entry 1 and Fig. 1a) with each urea pointing outside of the cavity.

**Table 2:** Key structural angles of 2–X topologies calculated on the APFD/6-31+G(d,p) level

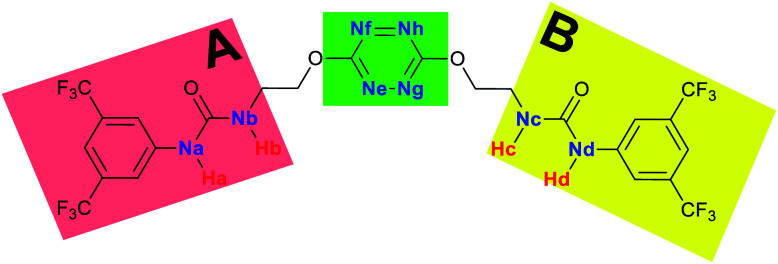							
Entry	Angles^a^	2	2–Cl	2–Br	2–I	2–SCN	2–PF_6_
1	Plane A/plane B	30.9	63.3	50.4	28.2	18.0	31.7
2	Plane A/tetrazine	74.7	88.1	90.0	79.3	62.2	51.0
3	Plane B/tetrazine	74.4	29.0	41.1	52.7	46.4	33.2

a Angles measured in degrees.

In the presence of spherical anions, in 2–Cl, 2–Br and 2–I, a conformation in which the two urea motifs are cross-shaped and face each other is observed (Fig. 1b). An angle of 63° is measured between the two planes A and B for 2–Cl. As the polarizability of the anion increases, the angle between planes A and B become more flattened (Table 2, entry 1). Moving from spherical to linear (SCN) and hexahedral (PF_6_) anions impacts the 3D-shape of the corresponding complexes. Orientation of aryl-urea planes with respect to tetrazine is characteristic of the molecular architecture of complexes. The A arm is almost orthogonal for 2–Cl and 2–Br. The orientation of this arm then become more flattened as the size of the anion increases (Table 2, entry 2). The B arm then adapts to the global conformation with more flattened angles towards tetrazine (Table 2, entry 3). In the case of the linear thiocyanate anion, the two lateral arms are almost parallel. Most likely, a favorable interaction between the electrodeficient aromatic ring of one arm and the urea of the other arm is observed as evidenced by the corresponding NCI plot (Table 1, entry 5).

Structural distances were also measured in order to see the influence of anion binding on different bonds in the case of complexation with spherical anions (Table 3). Receptor 2 displays almost the same four N–H bond lengths. Complexation of anions implies an increase of N–H bond lengths, significant of the complexation process (Table 3, entries 1 to 4). An increase of both the size and polarizability of the anion X^−^ implies a concomitant shortening of N–H bond lengths (Table 3, entries 1 to 4) and an elongation of H–X distances (Table 3, entries 5 to 8), characteristic of a decrease of the hydrogen bonding interaction. Influence of electrowithdrawing groups onto the H–X distances is observed (Table 3, entries 5 to 8). As the polarizability of the anion increases, the length of H–X bonds increases. In all complexes, a shortening of H–X distances is observed for H_a_ and H_d_ that are close to the bis(trifluoromethyl)phenyl group, compared to H_b_ and H_c_. Distances between anion and centroid of tetrazine ring were also determined (Table 3, entry 9).^30^ These distances are close for Cl^−^ (3.31 Å) and Br^−^ (3.42 Å) but markedly increased for I^−^ (3.79 Å). The orientation of the tetrazine ring towards the halide anions is also impacted by the presence of the two urea units. Indeed, in 2–Br, similar N_e_–X, N_f_–X, N_g_–X and N_h_–X distances are observed (Table 3, entries 10 to 13), representative of an almost cofacial arrangement between the tetrazine and the urea/Br planes. In contrast, in both 2–Cl and 2–I, N_e_–X, N_f_–X, N_g_–X and N_h_–X distances differ markedly from each other, inducing a twist of the tetrazine ring with respect to the 2–Br case.

**Table 3:** Key structural lengths of 2–X topologies calculated on the APFD/6-31+G(d,p) level

Entry	Bond^a^	2	2–Cl	2–Br	2–I
1	N_a_–H_a_	1.009	1.026	1.026	1.023
2	N_b_–H_b_	1.009	1.023	1.022	1.020
3	N_c_–H_c_	1.008	1.022	1.020	1.020
4	N_d_–H_d_	1.010	1.027	1.027	1.026
5	H_a_–X	—	2.190	2.343	2.617
6	H_b_–X	—	2.256	2.440	2.702
7	H_c_–X	—	2.243	2.464	2.745
8	H_d_–X	—	2.223	2.310	2.533
9	X–centroid	—	3.313	3.421	3.785
10	N_e_–X	—	3.494	3.761	4.109
11	N_f_–X	—	3.664	3.753	3.877
12	N_g_–X	—	3.561	3.851	4.223
13	N_h_–X	—	3.736	3.854	4.017

a Distances in Å.

### Synthesis

**Scheme 2:**
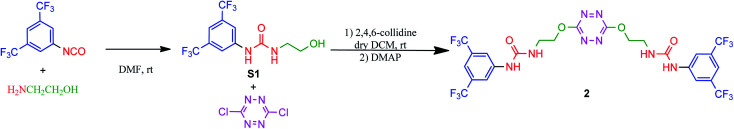
Synthesis of receptor 2.

Synthesis of receptor 2 was achieved using a two steps procedure involving the condensation of ethanolamine on the commercially available 3,5-bis-(trifluoromethyl)phenylisocyanate previously reported by us to form the intermediate urea S1 (Scheme 2).^27^ Secondly, the attachment of two arms on the tetrazine core required to set a one pot double S_N_Ar. First, it involves two equivalents of collidine to generate the intermediate 1 monitored by TLC but not isolated, and then four equivalents of 4-dimethylaminopyridine are added to generate receptor 2 in 38% yields. The newly formed receptor was fully characterized by spectroscopic and analytical methods (ESI pp. 21–26).†

### Tandem mass spectrometry

**Fig. 2:**
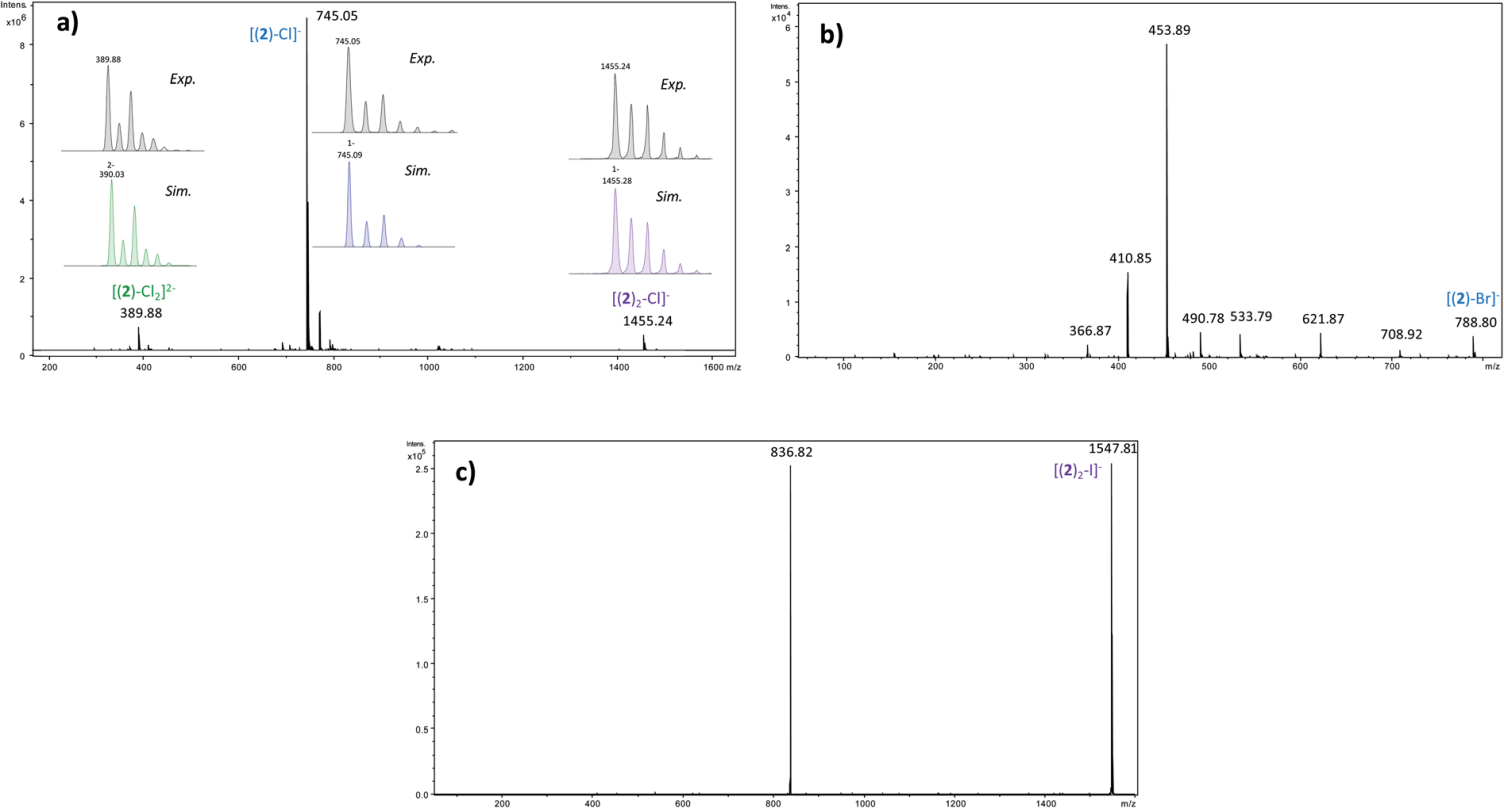
(a) Electrospray mass spectrum of an equimolar (10^−4^ M) mixture of 2/NBu_4_Cl (in inset comparison between the experimental and theoretical isotopic distributions of the various adducts) (b) MS/MS spectrum of the [(2)–Br]^−^ ion (*m*/*z* 789) – (c) MS/MS spectrum of the [(2)_2_–I]^−^ ion (*m*/*z* 1548).

In order to determine the structure of the complexes that can arise in the gas phase from the interaction of 2 with the different anions (X^−^), we performed a series of MS and MS^*n*^ experiments. To this end, 10^−4^ M equimolar mixtures of 2/Bu_4_NX were prepared in a 90/10 acetonitrile (ACN)/purified water solution, and electrosprayed into a 3D ion trap mass spectrometer (Bruker Amazon Speed ETD) with an Apollo II electrospray source (see ESI† for additional experimental details). Electrospray spectra obtained with the various anions are globally very similar. A typical ESI source spectrum is given in Fig. 2-a for NBu_4_Cl. Both the composition and stoichiometry of the complexes produced in the gas phase can be characterized thanks to their isotopic distribution (see inserts of Fig. 2-a for examples). For the sake of simplicity, the *m*/*z* values discussed all along this section correspond to the monoisotopic peak. Whatever the anionic partner, the ESI spectrum is dominated by a very intense [(2)–X]^−^ anion. [(2)_2_–X]^−^ adducts are systematically observed, but their intensity is significantly smaller. Depending on the experimental conditions, [(2)_3_–X]^−^ adducts could also be observed but generally in very weak abundance. The most intense complex therefore exhibits a 1 : 1 stoichiometry, and the experimental isotopic distributions observed indicate that we do not see [(2)_*n*_–X_*n*_]^*n*−^ complexes with *n* > 1. On the other hand, the rather long size of the anionic receptor 2 allows the incorporation of a second anion and the concomitant formation of [(2)–X_2_]^2−^ adducts (X = Cl, Br, I) as confirmed by the isotopic distribution of peaks separated by 0.5 Da (Fig. 2-a). Note that such doubly-charged complexes could not be observed with the smaller anion receptor 1.^27^

We then tried to obtain structural information about the [(2)_*n*_–X]^−^ anions. Starting with [(2)_2_–X]^−^ species, their unimolecular dissociation upon collision induced dissociation (CID) is solely characterized by the elimination of the intact receptor 2 (presently illustrated by Fig. 2-c), excepting the chloride adduct (X = Cl), which also expels a small fraction of hydrogen chloride (Fig. S10†). Then, we concentrated our effort to the study of the 1 : 1 complexes, namely the [(2)–X]^−^ ions adducts. To this end, we recorded a series of MS^n^ spectra (*n* = 2–4), which resulted in the following fragmentation scheme.

The overall fragmentation picture strongly depends on the nature of X. Like for the anionic receptor 1, the only fragmentation observed in presence of PF_6_^−^, is the formation of PF_6_^−^, again suggesting a very weak interaction in the gas phase with this anion. For the four remaining adducts, several common fragmentations are observed, and notably the elimination of HX, leading to deprotonated receptor [(2)–H]^−^ (*m*/*z* 709). Like with the smaller receptor 1, the extent of deprotonation of 2 and concomitant formation of HX is correlated to the gas-phase acidity of HX (or basicity of X^−^).^31^ It corresponds to the main primary process with and Cl^−^ and SCN^−^
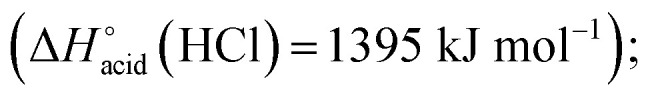

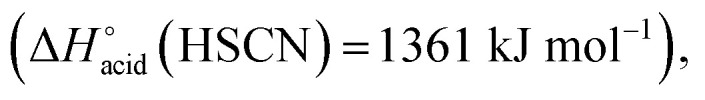
 which are more basic in the gas phase than Br^−^ and I^−^. Its abundance then logically decreased for Br^−^
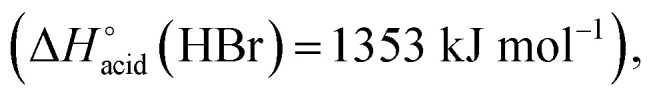
 and is even weaker for I^−^
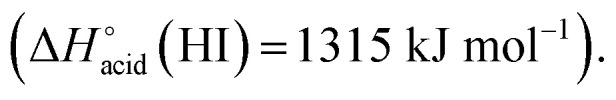
 In addition, MS^3^ experiments showed that the main fragment ions observed onto the MS/MS spectra of [(2)–SCN]^−^ and [(2)–Cl]^−^ ions, namely *m*/*z* 622 and 454, can arise from dissociation of [(2)–H]^−^, and according to our MS^4^ data, the former further dissociates to give *m*/*z* 324, whereas the latter gives rise to *m*/*z* 367, 199 and 156 ions. Consequently, for both Cl^−^ and SCN^−^ anions, most of the fragments ions observed imply preliminary formation of the deprotonated receptor, *m*/*z* 709. Reasonably, its formation may imply removal of one of the urea protons, and one may assume that removal of H_a_ or H_d_ should be more favorable due to resulting stabilization of the anionic charge through resonance with the aromatic ring. Removal of H_a_ is also consistent with the greater lengthening of N_a_–H_a_ and N_d_–H_d_ bonds with respect to N_b_–H_b_ and N_c_–H_c_ bonds evidenced by the calculations. Observation of deprotonation for all anions but PF_6_^−^ therefore indicates a strong interaction of the anion with the urea moiety, as evidenced by the geometrical parameters from theoretical study.

**Scheme 3:**
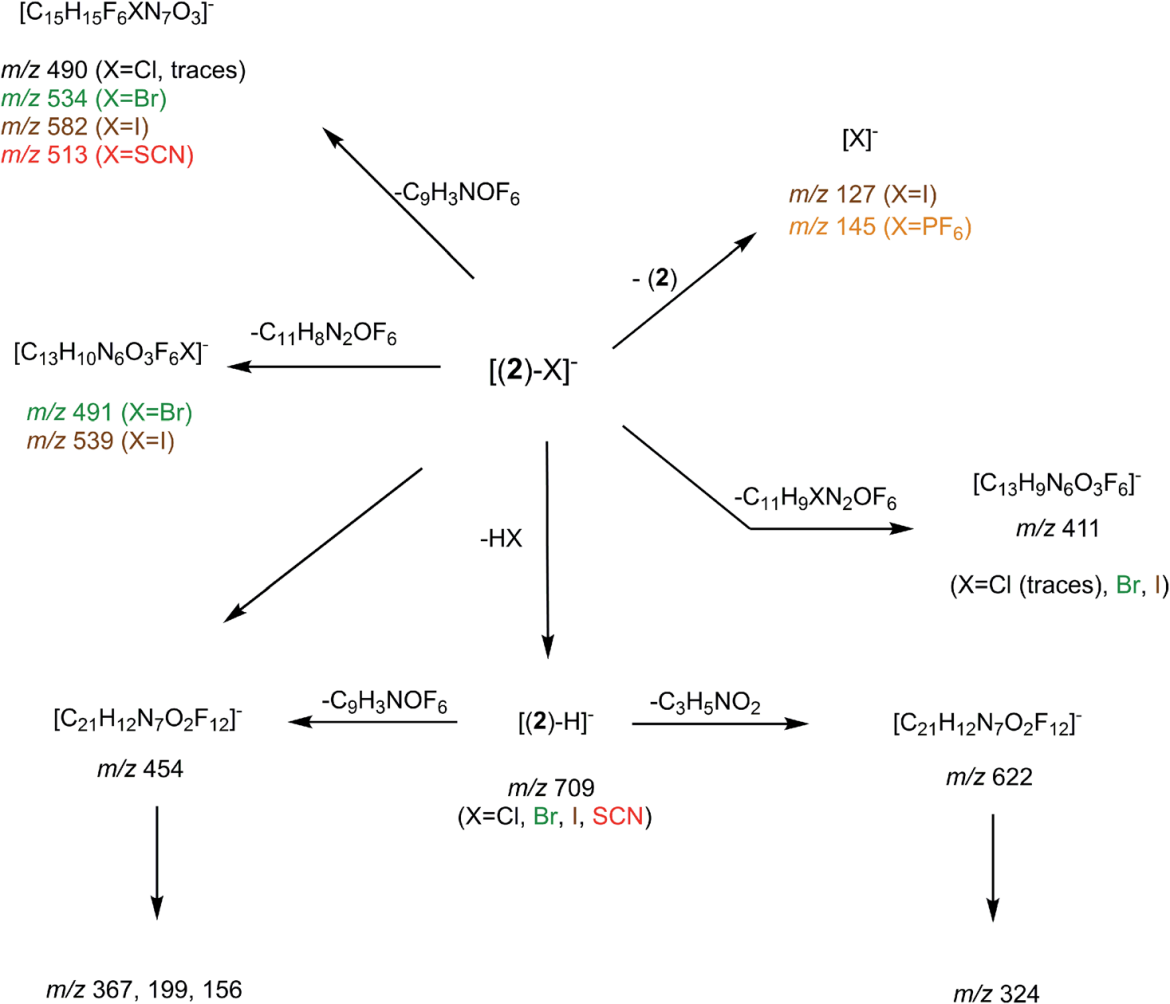
General dissociation scheme for the [(2)–X]^−^ complexes under CID conditions.

Fragment ions arising from alternate primary processes become significant with bromide and iodide anions, as the extent of formation of [(2)–H]^−^ decreases with the decrease of the basicity of X^−^. This is illustrated in Fig. 2-b, with the presence of *m*/*z* 534, 491, and 411. Note that the two former ions are shifted to *m*/*z* 582 and 539, respectively, with iodide. This means that the fragment ion generated incorporates the anion. Indeed, if one considers this second common fragmentation (top left of Scheme 3), the formation of [C_15_H_15_F_6_XN_7_O_3_]^−^ can be rationalized by assuming in a first step the direct attack of the tetrazine moiety by X^−^, followed by elimination of a C_9_H_3_NOF_6_ moiety. Consequently, this mechanism, together with the formation of [C_13_H_10_N_6_O_3_F_6_X]^−^, suggest an interaction of X^−^ with the tetrazine motif, prior to dissociation.

Finally, we also studied the fragmentation of the doubly-charged complexes observed with the halogens, [(2)–X_2_]^2−^. Their fragmentation spectra turned to be not informative, as characterized by a charge separation process leading to X^−^ and [(2)–X]^−^ ions.

### NMR titrations: fundamental state binding studies

The affinity of 2 towards Cl, Br, I, SCN and PF_6_ anions (as Bu_4_N^+^ salt) was assessed by ^1^H NMR titrations in CD_3_CN (Fig. S16 to S23†). The case of spherical anions is considered first. Linear and hexahedral anions will be discussed afterwards.

**Fig. 3:**
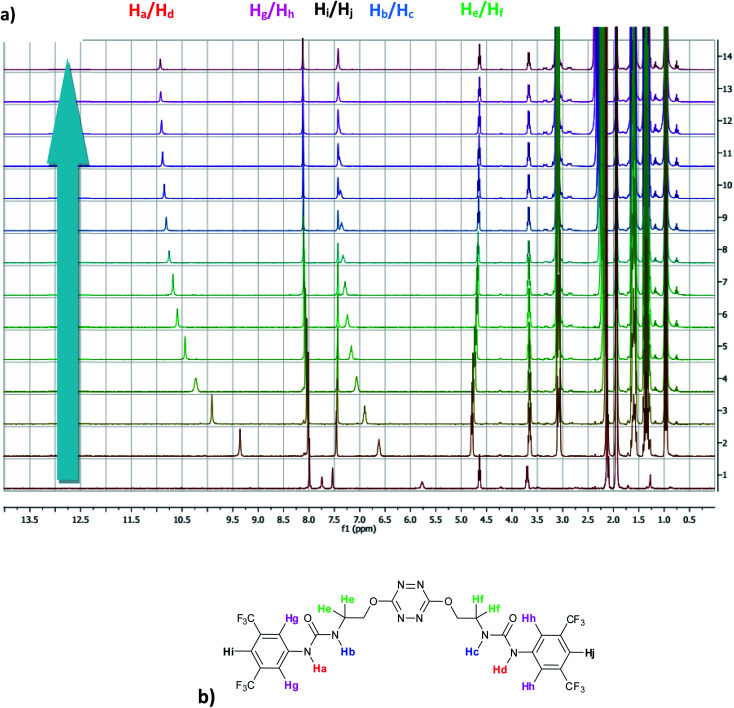
(a) NMR titration of anion receptor 2 by chloride anion (0 to 13 equivalents) (b) proton attribution.

**Fig. 4:**
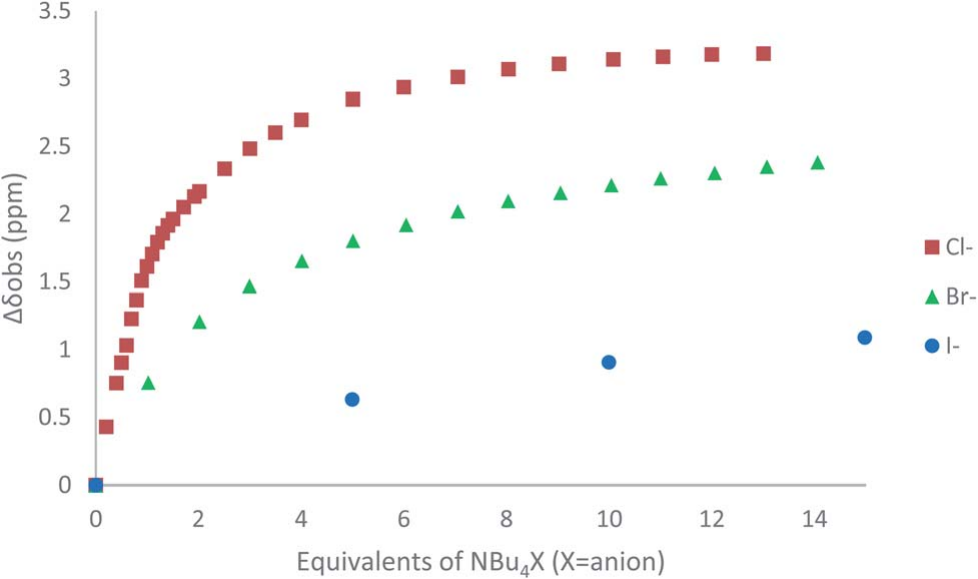
Δ*δ*_H_a_/H_d__ plotted as a function of the number of equivalents of NBu_4_X (X = Cl, Br and I) added for protons H_a_/H_d_.

^1^H NMR titration spectra of 2 with Cl^−^ is reported in Fig. 3. In CD_3_CN, the iterative addition of chloride anion to the receptor leads to substantial modifications of the ^1^H NMR spectrum. Mainly, a large downfield shift of the two mobile proton signals of both ureas is observed. Following the theoretical study and mass spectrometry experiments, a cooperative binding of the two urea partners to a similar extent is therefore observed. However, if both protons are downshifted, each of the two signals is differently impacted. H_a_/H_d_ undergoes a shift from 7.74 to 10.93 ppm (Δ*δ*_Cl–H_a_/H_d__ = 3.19 ppm) and H_b_/H_c_ from 5.77 to 7.43 ppm (Δ*δ*_Cl–H_b_/H_c__ = 1.66 ppm). The difference likely arises from the proximity of the electron withdrawing aromatic fragment proximal to H_a_/H_d_. These shifts are consistent with previously observed values for the monosubstituted compound 1.^27^ Titration was then extended to other spherical anions (Fig. S18 [Br] and S20 [I]†). The same trend was observed with Br (Δ*δ*_Br–H_a_/H_d__ = 2.74 ppm at 50 equivalents). With I, downfield shifts of urea protons arise compared to 1 (Δ*δ*_I–H_a_/H_d__ = 1.96 ppm at 150 equivalents). Once again, shifts of H_a_/H_d_ were increased compared to H_b_/H_c_. Comparison of differences in shifts observed as a function of the number of equivalents of salt added was plotted in Fig. 4. The reachpoint observed for each anion is decreasing with the size of the anion, implying a stronger interaction between Cl and 2 than between Br or I and 2 in decreasing order.

Titration was next performed on the linear anion SCN^−^. The same approach led to a ^1^H NMR spectrum which showed similar features (Fig. S22†). Signals of H_a_/H_d_ and H_b_/H_c_ are downshifted upon progressive addition of SCN^−^ with Δ*δ*_SCN–H_a_/H_d__ = 1.81 ppm and Δ*δ*_SCN–H_b_/H_c__ = 0.80 ppm, suggesting a weaker interaction with thiocyanate anion. Finally, in the case of PF_6_^−^, no significant shift was observed upon addition of 5 equivalents of the anion, implying a lack of interaction between 2 and PF_6_^−^.

As observed previously, the marked decrease of Δ*δ*_X–H_a_/H_d__ and Δ*δ*_X–H_b_/H_c__ moving from Cl^−^ to SCN^−^ is indicative of the general binding affinity trend between 2 and the anions. To clarify and confirm this tendency, binding constants of the corresponding complexes were determined using two different methods: a single proton method determined by Gonzàlez-Gaitano *et al.* using 1 : 1 host:guest binding model,^32^ and a global analysis of three protons shifts using SPECFIT® software.^33^ The first method is based on the differences in chemical shifts observed (Δ*δ*) plotted as a function of number of equivalents of NBu_4_X added (Table 4, Fig. 4, S16 to S24†).

**Table 4:** Binding constants calculated from NMR studies using mathematical equations and SPECFIT® software

Entry		*K*_A,H_a_/H_d__ a	*K*_A,H_b_/H_c__ a	*K*_A,H_g_/H_h__ a	*K*_A,SPECFIT_ a
1	2–Cl	459	487	61	424 ± 1 (0.4%)
2	2–Br	114	116	93	296 ± 1 (1.3%)
3	2–I	21	22	33	20 ± 1 (0.2%)
4	2–SCN	11	12	17	11 ± 1 (0.1%)
5	2–PF_6_	—b	—b	—b	—b

a Binding constants in L mol^−1^.

b No binding is observed.

As deduced from ^1^H NMR spectra analysis, all complexes fit well with 1 : 1 host guest binding model (relative errors of fits <1.5%) which is consistent with the hypothesis of folding in solution. 2–Cl exhibits the strongest binding constant of the series of anions studied (424 L mol^−1^). Moreover, a decrease is observed from 2–Br (296 L mol^−1^) to 2–I (20 L mol^−1^) and 2–SCN (11 L mol^−1^), and no binding constant could be determined for 2–PF_6_.

### Photophysical analysis: excited state binding studies

Based on interesting photophysical properties, the affinity of 2 towards Cl^−^, Br^−^, I^−^ and SCN^−^ (as Bu_4_N^+^ salts) was also measured by UV-visible and steady state fluorescence spectroscopies as well as time-resolved fluorescence in acetonitrile. Cases of chloride and bromide will be discussed first. In a second part, the behavior of I^−^ and SCN^−^ will be described.

The absorption spectrum of 2 was recorded in acetonitrile. It displays four absorption maxima at 520, 343, 290 and approx. 235 nm (Fig. S5†). A TDDFT calculation (PBE/6–311++G(d,p) level) has been done to assign these bands (Fig. S26 to S28†). The two former bands could be attributed to the n → π* and π → π* transitions of tetrazine, respectively, whereas the two latters could be assigned to π → π* transitions centered on urea moiety bearing a bis-3,5-trifluoromethylenephenyl substituent. Fluorescence emission displays the typical emission of tetrazine substituted by two oxygen atoms with a maximum at 572 nm. A quantum yield of 8.5% was measured in acetonitrile for 2 (Fig. S25†). These results are in line with our previous publications.^23,27^

**Fig. 5:**
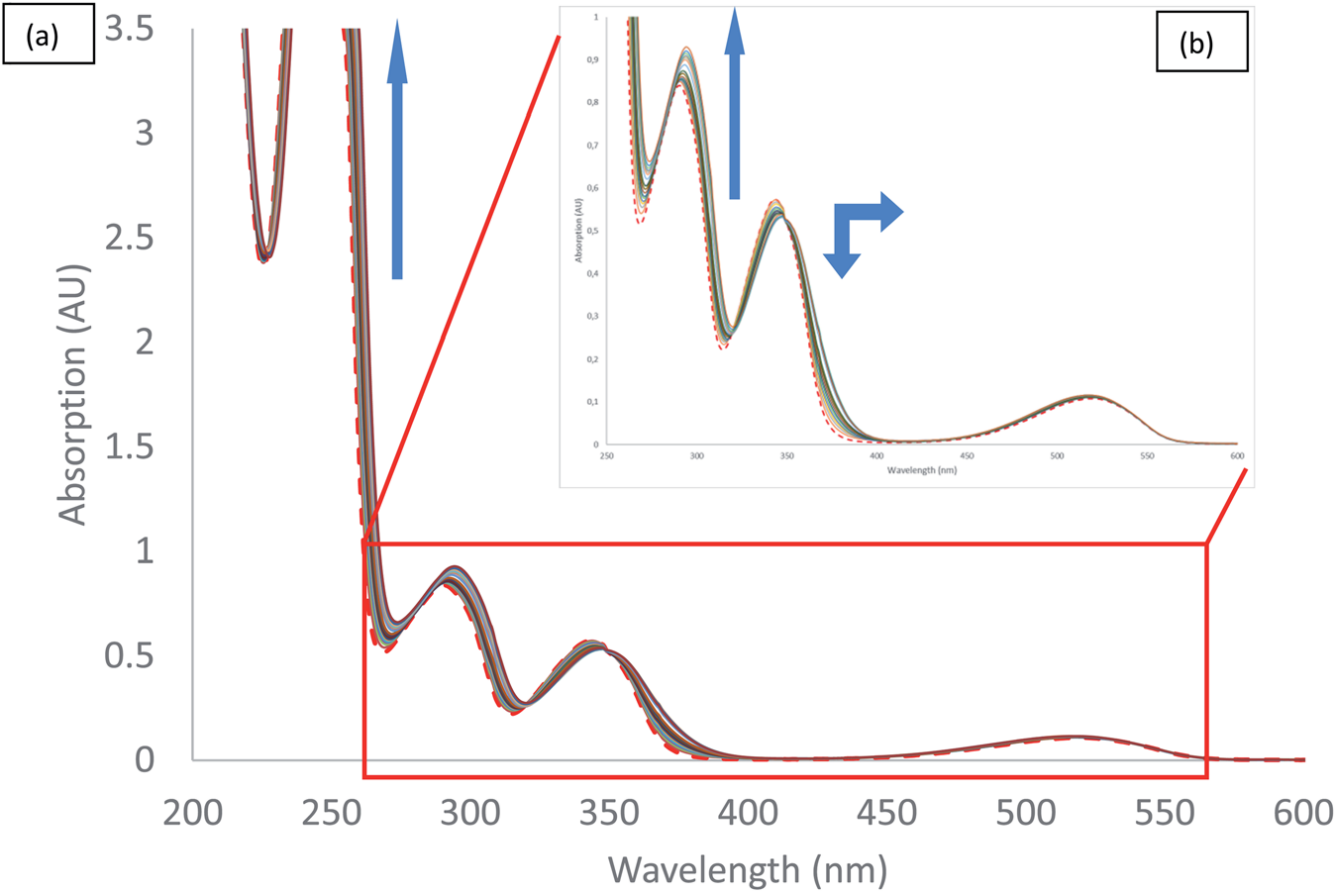
Absorption spectra measured during the titration of 2 with tetrabutylammonium chloride (0 to 11 equivalents of NBu_4_Cl): (a) total spectra, (b) zoom.

UV-visible spectra measured during the titration of 2 with chloride anion are reported in Fig. 5. We can observe different behaviours depending on the transition considered. Both hyperchromic and bathochromic shifts are observed on π–π* urea centred transitions due to the addition of chloride anion, consistent with a complexation mechanism. The π–π* bands of tetrazine moiety display a distinct behaviour with a hypochromic and bathochromic shift with the apparition of an isosbestic point at 349 nm. Similarly, an isosbestic point is observed in the case of bromide anion (Fig. S37†). Finally, the n–π* transition is weakly affected by the addition of chloride anion. Association constants for 1 : 1 binding model were determined using the SPECFIT software (Table 5). The value obtained for chloride anion is much larger than for other anions, indicative of a marked selectivity of receptor 2 for chloride anion.

**Table 5:** Binding constants calculated from photophysical experiments using mathematical equations and with SPECFIT® software

Entry		*K*_A,abs,SPECFIT_ a	*K*_A,fluo_ a	*K*_A,fluo,SPECFIT_ a	*K*_A,decay_ a
1	2–Cl	11 990 ± 1 (1%)	27 141	27 886 ± 1 (1%)	30 000
2	2–Br	196 ± 1 (2.1%)	1325	1267 ± 1 (1.0%)	1300
3	2–I	8 ± 1 (0.5%)	564	514 ± 1 (1.7%)	237
4	2–SCN	18 ± 1 (0.6%)	491	499 ± 1 (2.5%)	899
5	2–PF_6_	—b	—b	—b	—b

a Binding constants in L mol^−1^.

b No binding was observed.

In the case of both iodide and thiocyanate, UV-vis titrations (Fig. S45 [I^−^] and S53 [SCN^−^]†) showed small hyperchromic shifts of π–π* transitions of ureas. No isosbestic point and no significant shifts were observed for π–π* and n–π* transitions of tetrazine. From these results, we can conclude that the major interaction involved in the complexation process is the hydrogen bonding with ureas.

**Fig. 6:**
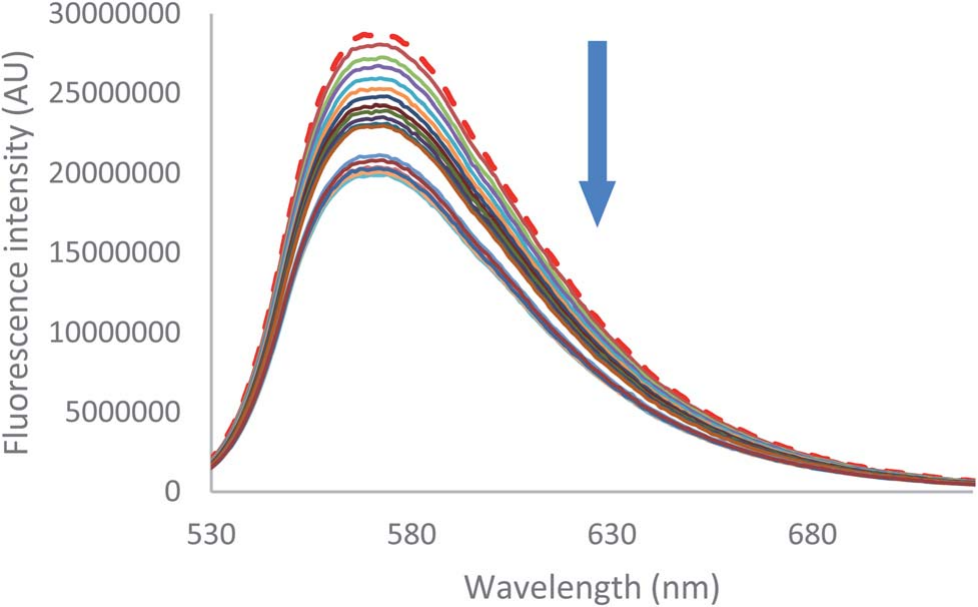
Fluorescence spectra measured during the titration of 2 with tetrabutylammonium chloride (0 to 11 equivalents of NBu_4_Cl).

Emission quenching is also anion dependant. As can be seen for both chloride and bromide (Fig. 6 [Cl^−^] and S39 [Br^−^]),† partial emission quenching is noted. A weak interaction between the tetrazine moiety and the anion is therefore underlined, which is attributed to the formation of a weak anion–π interaction. This property has been already used in the characterization of anion–π interaction in solution by us^27^ and others.^34^ Despite the large excess of salts added, a complete quenching cannot be reached. This behaviour indicates that complexes between 2 and chloride or bromide anions, respectively, exhibit a residual fluorescence. Such phenomenon will be further studied by time-resolved fluorescence.

**Fig. 7:**
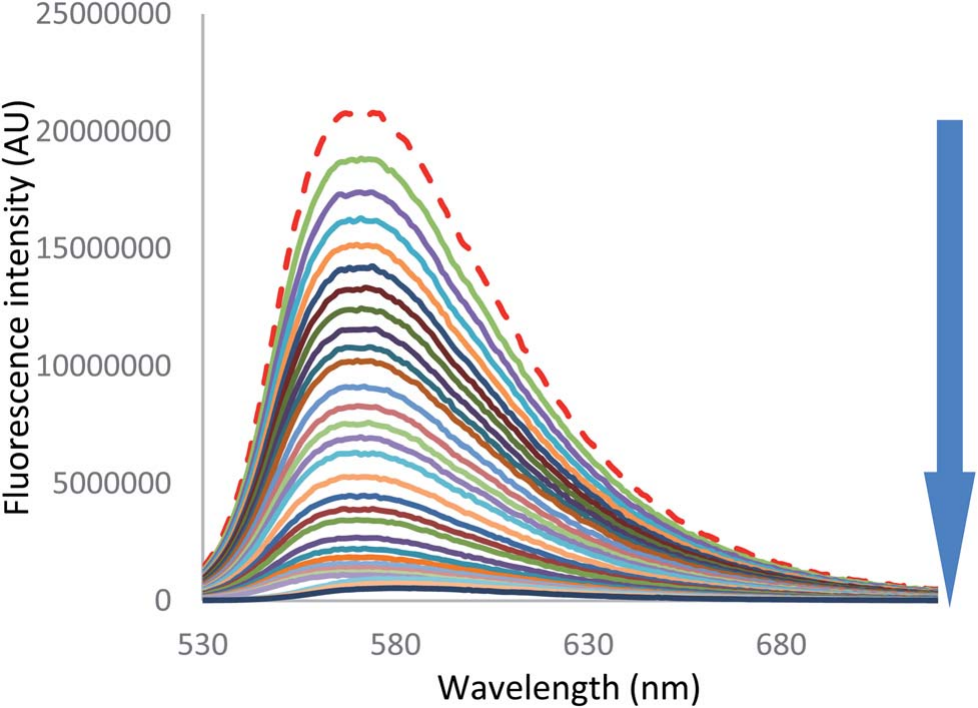
Fluorescence spectra measured during the titration of 2 with tetrabutylammonium iodide (0 to 200 equivalents of NBu_4_I).

On the other hand, in the case of iodide and thiocyanate anions (Fig. 7 [I^−^] and S55 [SCN^−^]),† addition of a large excess of anion leads to complete fluorescence quenching, characteristic of a stronger anion–π interaction with more polarizable anions.

From these data, new values of association constants were extracted (Table 5) by employing two methods: a global spectral analysis with the SPECFIT software (*K*_A,fluo SPECFIT_) and a single wavelength nonlinear least squares analysis (*K*_A,fluo_, see ESI† for details). Once again, 1 : 1 host : guest binding model was adapted to all complexes studied. A higher value was observed for chloride anion (27 886 L mol^−1^), followed by bromide anion (1267 L mol^−1^). Iodide (514 L mol^−1^) and thiocyanate (499 L mol^−1^) anions display similar binding constants. No binding constants could be determined for PF_6_^−^.

**Fig. 8:**
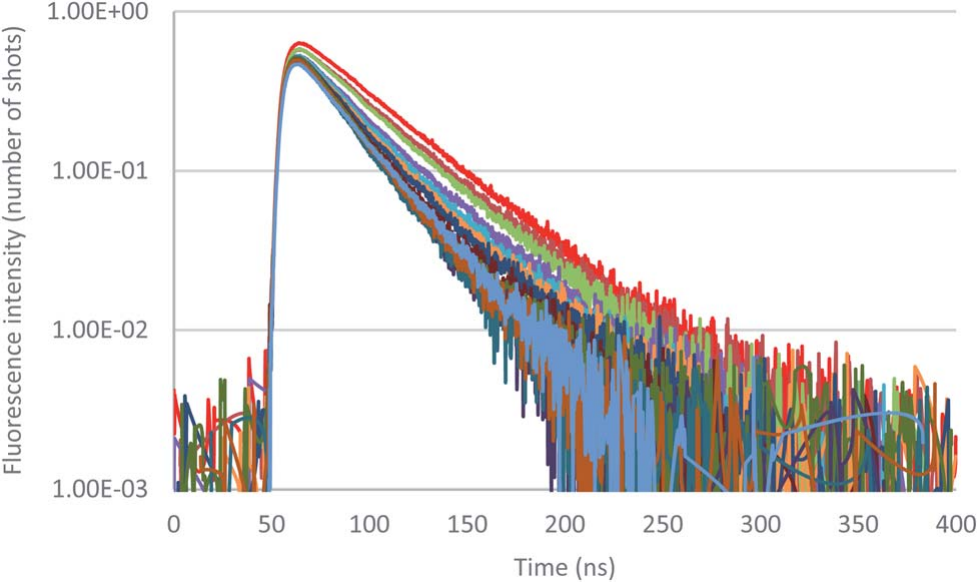
Fluorescence decay titration of 2 with tetrabutylammonium chloride (0 to 11 equivalents of NBu_4_Cl), logarithmic scale.

Further insight in the excited state behavior was gained by recording fluorescence decays during the titrations of the various anions. Fluorescence decays of 2 recorded for the titration with tetrabutylammonium chloride are presented in Fig. 8 and S36.† Upon addition of the chloride anion, the initial fluorescence intensity is weakly affected, whereas fluorescence lifetime decreases from 43 ns to 25 ns at one equivalent. Above one equivalent, the fluorescence lifetime remains almost constant. A second exponential with a larger lifetime (∼60 ns) is necessary to fit the decays. The contribution of this second lifetime remains minor during the entire titration. It can therefore be concluded from this titration that 2–Cl is slightly fluorescent and that the tetrazine–anion interaction is mainly dynamic. Moving on to Br^−^ anion (Fig. S42–S44†), a unique lifetime attributed to the formation of a fluorescent complex with a residual lifetime of 22 ns was observed. Therefore, small anions show a weak anion–π interaction, and an equilibrium between complexed and uncomplexed tetrazine is proposed that leads to the dynamic quenching observed (interaction is occurring after excitation of the tetrazine).

In the case of iodide (Fig. S50 to S52†) and thiocyanate (Fig. S58 to S60†) anions, the behavior is sensibly different since a much larger drop of the initial fluorescence intensity accompanies the shortening of the decay. A more static quenching is observed in this case. An interaction between more polarizable anions therefore exists in the ground state before excitation. After excitation, the tetrazine residue becomes a strong electron attractor, leading to a dynamic quenching.

### Impact of double-substitution on receptor properties

Up to date, anion receptors combining on a tetrazine core and a hydrogen bond donor are scarcely described.^35,36^ Only one aliphatic mono-substituted tetrazine receptor has been recently reported by us.^27^ The impact of a double substitution on receptor properties was therefore evaluated based on descriptors such as (a) geometrical parameters (b) NMR shifts (c) photophysical properties and (d) association constants determined by NMR experiments.

#### (a) Geometrical parameters

**Fig. 9:**
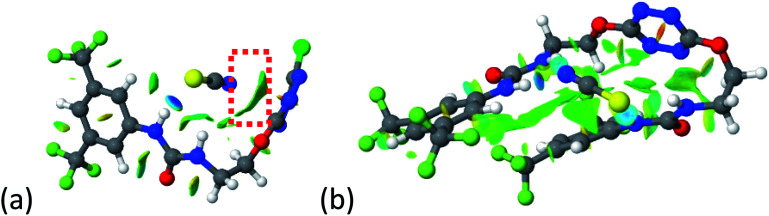
NCIplot of (a) 1–SCN and (b) 2–SCN complexes.

2–anion complexes show marked decrease of N–H bond lengths compared to 1–anion (Fig. S61,† 1.030 to 1.037 Å for 1–Cl *vs.* 1.022 to 1.027 Å for 2–Cl).^27^ An increase of anion-centroid distances is observed in the case of 2 (3.12 Å for 1–Cl *vs.* 3.31 Å for 2–Cl). These observations are in line with a decrease of the potent non-covalent interactions. A significant change in interactions involved is observed in the case of 2–SCN. In 1–SCN complex, the nitrogen atom points out towards the tetrazine ring, enabling the establishment of an anion–π interaction. In 2–SCN complex, a loss of the anion–π interaction is observed, as the nitrogen of thiocyanate anion now points towards the second urea (Fig. 9).^27^

#### (b) NMR shifts

**Fig. 10:**
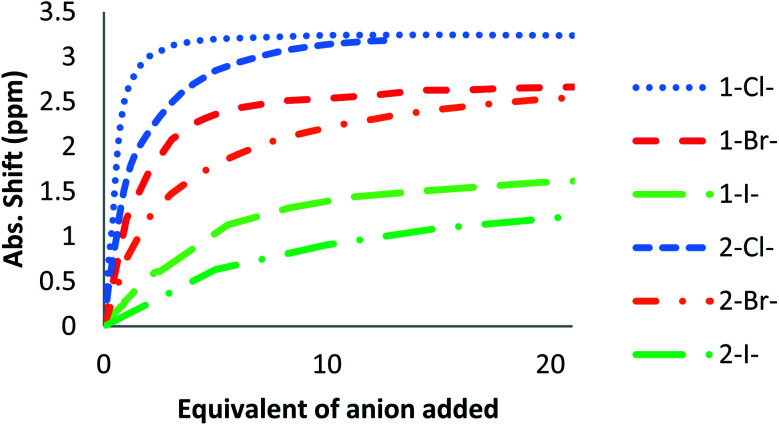
Absolute NMR Shifts for H_a_ in 1 and 2 during the titration of different anions.

The main difference between 1 and 2 lies in the initial shifts at low concentration of anion during titration (Fig. 10 and S63†). For instance, at one equivalent of added salt, H_a_/H_d_ shifted by 1.61 ppm for 2 whereas H_a_ for 1 was shifted by 2.59 ppm. Compared to 1 bearing a unique urea, interaction of anions with 2 is consequently shared between the two ureas, leading to a less potent unshielding effect on each urea.

#### (c) Photophysical properties

Double substitution led to a significant change in photophysical signature compared to 1. A large drop on fluorescence quantum yield is highlighted (39% for 1*vs.* 8.5% for 2), consistent with our previously reported study.^23^

#### (d) Association constants

A marked difference on association constants is observed both in NMR and photophysical experiments (Fig. S64†). Moreover, large discrepancies between values obtained by NMR and photophysical experiments are observed (Fig. S64 and S65†). This is in line with previously reported studies.^37,38^ Some hypothesis were made to explain disparities observed between association constants determined by different analytical procedures, such as the role of self-associating processes at high concentrations,^37^ or photoinduced phenomena at the excited state.^38^ In the following discussion, we will focus on general trends. Globally, binding constants trends are identical in NMR and photophysical experiments for iodide and thiocyanate anions, inducing the favorable binding properties of 1 towards 2. However, bromide and chloride complexes deserve further attention. Similar values are obtained for 1–Br and 2–Br complexes, displaying a change in selectivity as the size and the polarizability of the anion decrease. Using photophysical experiments, a marked selectivity is visible for 1–Cl and more pronounced with 2–Cl. However, such selectivity is inversed when comparing NMR titrations of 1–Cl and 2–Cl. To explain such behavior, we hypothesize that self-aggregation processes prevent the observation of this selectivity at high concentrations used for NMR experiments, as already observed by Albrecht.^37^ Regarding the respective behaviour of 1 and 2, an anti-cooperative effect between the five non-covalent interactions should be involved in the complexation process as defined by Sastry *et al.*^39^

## Conclusion

In summary, this paper reports the design of a new anion receptor based on a tetrazine core substituted by two arms bearing hydrogen bond donors. DFT calculations enabled to predict a substantial and adaptable folding of this receptor around different anions like chloride, bromide, iodide, thiocyanate and hexafluorophosphate. Based on these insightful preliminary investigations, we successfully synthetized the novel tetrazine based receptor 2. Application to the complexation of anions is reported in both ground and excited states. First, the mass spectrometry study demonstrates the prominent formation in the gas phase of the 1 : 1 stoichiometry complex [(2)–X]^−^, but also the strong interaction of the anion with both the urea moiety and the tetrazine group, except for PF_6_^−^, which exhibits a very weak interaction. NMR titrations in solution enable the characterization of 1 : 1 host : guest complexes and the determination of association constants. Photophysical properties and association constants were also determined using UV-visible and steady state fluorescence spectroscopies, confirming the tendency observed in NMR experiments. Furthermore, time-resolved fluorescence techniques highlighted a combination of both static and dynamic quenching. While smaller anions (Cl^−^, Br^−^) display a preponderant dynamic quenching, larger anions (I^−^, SCN^−^) show a more prominent contribution of static quenching. A marked selectivity for chloride anion was observed in both ground and excited states. An interesting anti-cooperative behavior was highlighted in 2 compared to 1. We believe that this work will help the design of future anion receptors combining hydrogen bonding and anion–π interaction. Further studies using other anion receptors are under way and will be reported in due time.

## Conflicts of interest

There are no conflicts to declare.

## Supplementary Material

RA-011-D1RA00630D-s001
