# Supplementary material for: Design and property investigation on a five-interaction-based fluorescent anion receptor clip
Source: RSC Adv. 2021 Mar 3;11(16):9476–87. doi: 10.1039/d1ra00630d (PMC8695457; doi:10.1039/d1ra00630d)
Supplement: RA-011-D1RA00630D-s001 [file RA-011-D1RA00630D-s001.pdf]

Electronic Supplementary Material (ESI) for RSC Advances.
This journal is © The Royal Society of Chemistry 2021

SUPPORTING INFORMATION

Design and Properties Investigation on a Five-Interaction-based Fluorescent Anion Receptor Clip

Romain PLAIS,[a] Hamza BOUFROURA,[a] Guy GOUARIN,[a] Anne GAUCHER,[a] Violette HALDYS,[b,c] Arnaud BROSSEAU,[d] Gilles CLAVIER,[d] Jean-Yves SALPIN,[b,c] and Damien PRIM,*[a]

a. *Université Paris-Saclay, UVSQ, CNRS, Institut Lavoisier de Versailles, 78035 Versailles (France)*
b. *Université Paris-Saclay, Univ Evry, CNRS, LAMBE, 91025 Evry (France)*
c. *LAMBE, CY Paris Cergy Université, CNRS, 95000 Cergy (France)*
d. *Université Paris-Saclay, ENS Paris-Saclay, CNRS, PPSM, 91190 Gif-sur-Yvette (France)*

Table of contents

1. General procedures, Material and Instrumentation 6
   1.1 General experimental procedures and Materials 6
   1.2 Instrumentation 6
   1.3 Molecular modelling and software 7
2. Computational Data of 2, anions and 2-anion complexes 7
   2.1 Coordinates of computed structures 7
   2.2 Planes defined using Chimera Software 20
3. Synthetic procedures and Characterization data 21
   3.1 Preparation of 1-(3,5-bis(trifluoromethyl)phenyl)-3-(2-hydroxyethyl)urea **S1** 21
   3.2 Preparation of 1,1'-(((1,2,4,5-tetrazine-3,6-diyl)bis(oxy))bis(ethane-2,1-diyl))bis(3-(3,5-bis(trifluoromethyl)phenyl)urea) **2** 21
4. Mass spectrometry experiments 27
   4.1 Fragmentation mechanisms associated to *Scheme 3* 27
   4.2 Typical MS/MS spectra obtained for the different adducts 30
5. NMR Titrations 32
   5.1 Practical analysis procedure 32
   5.2 Titration of **2** with NBu_4Cl 33
   5.3 Titration of **2** with NBu_4Br 35
   5.4 Titration of **2** with NBu_4I 37
   5.5 Titration of **2** with NBu_4SCN 39
   5.6 Superposition of experimental curves 41
6. Photophysical analysis and procedures 42
   6.1 General practical analysis procedure 42
   6.2 Determination of quantum yield of **2** 43
   6.3 Time Dependent DFT analysis of compound **2** 44
   6.4 Titration of **2** with NBu_4Cl 45
   6.5 Titration of **2** with NBu_4Br 50
   6.6 Titration of **2** with NBu_4I 55
   6.7 Titration of **2** with NBu_4SCN 60

7. Comparison of **1** and **2** 65
7.1 Geometrical parameters 65
7.2 NMR shifts 66
7.3 Association constants 67
8. Bibliography 68

Table of illustrations

Figure S1 : General procedure for Synthesis of **2** 21
Figure S2: ^{1}H NMR (300 MHz) spectrum of **2** in acetone d6 22
Figure S3: ^{13}C NMR (75 MHz) spectrum of **2** in acetone d6 23
Figure S4: ^{19}F NMR (282 MHz) spectrum of **2** in acetone d6 23
Figure S5: Absorption spectrum of **2** in acetonitrile 24
Figure S6: Fluorescence spectrum of **2** in acetonitrile 24
Figure S7: Mass spectrum of **2** (TOF ES+). Note also the presence of PDMS contaminant 25
Figure S8: Single mass analysis of **2** (TOF ES+) 26
Figure S9: Infrared spectrum of **2** 27
Figure S9: MS/MS spectrum of the [(**2**)$_2$-Cl]$^-$ adduct 31
Figure S10: MS/MS spectrum of the [(**2**)-SCN]$^-$ adduct 31
Figure S11: MS/MS spectrum of the [(**2**)-Cl]$^-$ adduct 32
Figure S12: MS/MS spectrum of the [(**2**)-I]$^-$ adduct 32
Figure S13: MS/MS spectrum of the [(**2**)-PF_6]$^-$ adduct 33
Figure S14: ^{1}H NMR titration of **2** with tetrabutylammonium chloride (0 to 13 equivalents) %c = percentage of complex %a = percentage of free receptor Δδcalc = chemical shift calculated Δδobs = chemical shift observed 34
Figure S15: Determination of binding constant using SPECFIT software for the ^{1}H NMR titration of **2** with tetrabutylammonium chloride 35
Figure S16: ^{1}H NMR titration of **2** with tetrabutylammonium bromide (0 to 50 equivalents) %c = percentage of complex %a = percentage of free receptor Δδcalc = chemical shift calculated Δδobs = chemical shift observed 36
Figure S17: Determination of binding constant using SPECFIT software for the ^{1}H NMR titration of **2** with tetrabutylammonium bromide 37
Figure S18: ^{1}H NMR titration of **2** with tetrabutylammonium iodide (0 to 150 equivalents) %c = percentage of complex %a = percentage of free receptor Δδcalc = chemical shift calculated Δδobs = chemical shift observed 38
Figure S19: Determination of binding constant using SPECFIT software for the ^{1}H NMR titration of **2** with tetrabutylammonium iodide 39
Figure S20: ^{1}H NMR titration of **2** with tetrabutylammonium thiocyanate (0 to 320 equivalents) %c = percentage of complex %a = percentage of free receptor Δδcalc = chemical shift calculated Δδobs = chemical shift observed 40

Figure S21: Determination of binding constant using SPECFIT software for the ^{1}H NMR titration of **2** with tetrabutylammonium thiocyanate 41
Figure S22: Differences in chemical shifts observed as a function of the number of equivalents of salts added protons a, b and c 42
Figure S23: Emission curves of compound **2** and reference in reference conditions 44
Figure S24: Compounds studied by time dependent DFT analysis 45
Figure S25: Experimental (orange) and calculated (blue) absorption spectra 45
Figure S26: Calculated transitions (major transitions in bold) 46
Figure S27: Experimental UV-Visible spectra measured during the titration of **2** with NBu_4Cl (0 to 10,5 equivalents) 46
Figure S28: Determination of binding constant using SPECFIT software for the UV-Visible titration of **2** with tetrabutylammonium chlorid 47
Figure S29: Experimental fluorescence spectra during the titration of **2** with NBu_4Cl (0 to 14,4 equivalents) 48
Figure S30: Mathematical fit during the fluorescence titration of **2** with NBu_4Cl (0 to 70 equivalents) and determination of the association constant 48
Figure S31: Determination of binding constant using SPECFIT software for the fluorescence titration of **2** with tetrabutylammonium chloride 49
Figure S32: Fluorescence decay titration of **2** with NBu_4Cl (0 to 11,6 equivalents) Logarithmic scale 50
Figure S33: Mathematical fit during the fluorescence decay titration of **2** with NBu_4Cl (0 to 11,6 equivalents) and determination of the association constant 50
Figure S34: Analysis of fluorescence decay titration of **2** with NBu_4Cl 51
Figure S35: Experimental UV-Visible spectra measured during the titration of **2** with NBu_4Br (0 to 160 equivalents) 51
Figure S36: Determination of binding constant using SPECFIT software for the UV-Visible titration of **1** with tetrabutylammonium bromide 52
Figure S37: Experimental fluorescence spectra during the titration of **2** with NBu_4Br (0 to 160 equivalents) 53
Figure S38: Mathematical fit during the fluorescence titration of **2** with NBu_4Br (0 to 160 equivalents) and determination of the association constant 53
Figure S39: Determination of binding constant using SPECFIT software for the fluorescence titration of **2** with tetrabutylammonium bromide 54
Figure S40: Fluorescence decay titration of **2** with tetrabutylammonium bromide (0 to 160 equivalents) Logarithmic scale 55
Figure S41: Mathematical fit during the fluorescence decay titration of **2** with NBu_4Br (0 to 160 equivalents) and determination of the association constant 55
Figure S42: Analysis of fluorescence decay titration of **2** with tetrabutylammonium bromide 56
Figure S43: Experimental UV-Visible spectra measured during the titration of **2** with NBu_4I (0 to 200 equivalents) 56

Figure S44: Determination of binding constant using SPECFIT software for the UV-Visible titration of **2** with tetrabutylammonium iodide 57
Figure S45: Experimental fluorescence spectra during the titration of **2** with NBu_4I (0 to 200 equivalents) 58
Figure S46: Mathematical fit during the fluorescence titration of **2** with NBu_4I (0 to 200 equivalents) and determination of the association constant 58
Figure S47: Determination of binding constant using SPECFIT software for the fluorescence titration of **2** with tetrabutylammonium iodide 59
Figure S48: Fluorescence decay titration of **2** with tetrabutylammonium iodide (0 to 200 equivalents) Logarithmic scale 60
Figure S49: Mathematical fit during the fluorescence decay titration of **2** with NBu_4I (0 to 200 equivalents) and determination of the association constant 60
Figure S50: Analysis of fluorescence decay titration of **2** with tetrabutylammonium iodide 61
Figure S51: Experimental UV-Visible spectra measured during the titration of **2** with NBu_4SCN (0 to 300 equivalents) 61
Figure S52: Determination of binding constant using SPECFIT software for the UV-Visible titration of **2** with tetrabutylammonium thiocyanate 62
Figure S53: Experimental fluorescence spectra during the titration of **2** with NBu_4SCN (0 to 300 equivalents) 63
Figure S54: Mathematical fit during the fluorescence titration of **2** with NBu_4SCN (0 to 300 equivalents) and determination of the association constant 63
Figure S55: Determination of binding constant using SPECFIT software for the fluorescence titration of **2** with tetrabutylammonium thioisocyanate 64
Figure S56: Fluorescence decay titration of **2** with tetrabutylammonium thiocyanate (0 to 300 equivalents) Logarithmic scale 65
Figure S57: Mathematical fit during the fluorescence decay titration of **2** with NBu_4SCN (0 to 300 equivalents) and determination of the association constant 65
Figure S58: Analysis of fluorescence decay titration of **2** with tetrabutylammonium thiocyanate 66
Figure S59: Geometrical parameters of receptors **1** and **2** 67
Figure S60: Absolute NMR shifts for H_a **1** and **2** 67
Figure S61: Absolute NMR shifts for H_b **1** and **2** 67
Figure S62: Association constants measured by NMR titrations of **1** and **2** with different anions 68
Figure S63: Association constants measured by photophysical titrations of **1** and **2** with different anions 69

Table of schemes

Scheme S1 Formation of $[C_{15}H_{15}F_6XN_7O_3]^-$ 26
Scheme S2 Formation of $[C_{13}H_{10}N_6O_3F_6X]^-$ 26
Scheme S3 Formation of *m/z* 622 from [(**2**)-H]$^-$ 27
Scheme S4 Formation of *m/z* 454 from [(**2**)-X]$^-$ and [(**2**)-H]$^-$ 28

1. General procedures, Material and Instrumentation

1.1 General experimental procedures and Materials

Unless otherwise noted, all starting materials were obtained from commercial suppliers and used without purification. N,N-Dimethylformamide (100mL, Anhydrous, 99.8%) was purchased at Sigma-Aldrich. Dichloromethane was distilled over Sodium and under argon. For NMR titrations, deuterated acetonitrile (99.80% D) was purchased in 0.75mL precoated bulbs from Eurisotop®. For photophysical analysis, acetonitrile RS –SPECTROSOL – For optical spectroscopy was purchased from Carlo Erba®.

Reaction progress was carried out using pre-coated TLC sheets ALUGRAM® Xtra SIL G/UV_{254} (0.20mm) from Macherey-Nagel® and visualized under 254 and 365 nm UV lamp from Fisher Bioblock Scientific®. Flash chromatography were proceeded using Silica 60M (0.04-0.063mm) for column chromatography silica gel from Macherey-Nagel®.

1.2 Instrumentation

^{1}H NMR spectra were recorded with Bruker AV-I 300MHz spectrometer at 298K, referenced to TMS signal and were calibrated using residual proton in Acetone d6 (δ=2.05ppm), according to the literature. [1] ^{19}F NMR spectra were recorded with Bruker AV-I 300MHz spectrometer at 282MHz and 298K and were not calibrated. ^{13}C NMR spectra were recorded with a Bruker AV-I 300MHz spectrometer at 75MHz and 298 K and were calibrated using Acetone d6 (δ = 30.60 ppm). [1] ^{1}H NMR spectroscopic data are reported as follow: chemical shift δ [parts per million] (multiplicity, coupling constants in Hertz, integration). Multiplicities are reported as follow: s = singlet, d = doublet, t = triplet, q = quadruplet, quint = quintuplet, sext = sextuplet, hept = heptuplet, dd = doublet of doublet, td = triplet of doublet, tt = triplet of triplet, ddd = doublet of doublet of doublet, m = multiplet. ^{13}C NMR spectroscopic data are reported in terms of chemical shifts δ [ppm] and when it is necessary multiplicity and coupling constant in Hertz.

To check the structure of the product obtained during the synthesis, high resolution mass spectra (HRMS) were obtained with a Waters Xevo QTOF instrument fitted with an electrospray ionization source (ESI+), using Leucine Enkephaline solution as internal calibrant.

Interactions of **2** with the various anions occurring in the gas phase were studied with a 3D ion trap instrument (Bruker Amazon Speed ETD). Complexes were generated in the gas phase by electrospray. To this end, equimolar mixtures of **2**/NBu_4X were prepared. Starting from 5 10^{-2}M stock solutions of **2** and NBu_4X solubilized in acetonitrile (ACN) and purified water, respectively, 10^{-4} M mixtures of **2**/ NBu_4X (90/10 ACN/H_2O) were introduced in the electrospray source by a syringe pump (3 µL/min). Typical experimental conditions were as followed: Capillary voltage: - 4000 V; End plate offset : -550 V; Dry gas: 5 L/min / Dry gas temperature: 200 °C, Nebuliser gas : 7.3 PSI ; Cap exit: -140 V; Trap Drive 49.5.

All spectra were recorded in the "Maximum Resolution mode"
MS analysis : ICC mode : "on" and acquisition time : auto.
MS^n analysis : ICC mode off / accumulation time 1 to 5 ms / Isolation window 6 to 10 Da / Fragmentation delay 40 ms/ amplitude of fragmentation : 0.20-1.0 depending on the ions.

UV-Visible spectra were recorded at 25°C on a Cary 400 (Agilent) double-beam spectrometer using a 10 mm path quartz cell.

Emission spectra were measured on a Fluoromax-3 (Horiba) or a Fluorolog-3 (Horiba) spectrofluorometer. An angle configuration of 90° was used. Optical density of the samples was checked to be less than 0.1 to avoid reabsorption artifacts.

Fluorescence decay curves were obtained using an Edinburgh instrument LP920 laser flash photolysis spectrometer combined with an Nd:YAG laser (Continuum) doubled at 530 nm via non linear crystals. This second harmonic is optimized to pump an OPO. The fluorescence photons were detected at 90° through a long pass filter (GG385 SCHOTT) and a monochromator by means of a Hamamatsu R928 photomultiplier. The Levenberg-Marquardt algorithm was used for non-linear least square fit (tail fit) as implemented in the L900 software (Edinburgh instrument). In order to estimate the quality of the fit, the weighted residuals were calculated.

Infrared spectrum was obtained using a Nicolet 6700 FTIR-CsI Spectrometer using a ATR SMART ORBIT modulus.

1.3 Molecular modelling and software

All calculations were carried out using Gaussian 09® program:

Gaussian 09, Revision D.01, M. J. Frisch, G. W. Trucks, H. B. Schlegel, G. E. Scuseria, M. A. Robb, J. R. Cheeseman, G. Scalmani, V. Barone, B. Mennucci, G. A. Petersson, H. Nakatsuji, M. Caricato, X. Li, H. P. Hratchian, A. F. Izmaylov, J. Bloino, G. Zheng, J. L. Sonnenberg, M. Hada, M. Ehara, K. Toyota, R. Fukuda, J. Hasegawa, M. Ishida, T. Nakajima, Y. Honda, O. Kitao, H. Nakai, T. Vreven, J. A. Montgomery, Jr., J. E. Peralta, F. Ogliaro, M. Bearpark, J. J. Heyd, E. Brothers, K. N. Kudin, V. N. Staroverov, T. Keith, R. Kobayashi, J. Normand, K. Raghavachari, A. Rendell, J. C. Burant, S. S. Iyengar, J. Tomasi, M. Cossi, N. Rega, J. M. Millam, M. Klene, J. E. Knox, J. B. Cross, V. Bakken, C. Adamo, J. Jaramillo, R. Gomperts, R. E. Stratmann, O. Yazyev, A. J. Austin, R. Cammi, C. Pomelli, J. W. Ochterski, R. L. Martin, K. Morokuma, V. G. Zakrzewski, G. A. Voth, P. Salvador, J. J. Dannenberg, S. Dapprich, A. D. Daniels, O. Farkas, J. B. Foresman, J. V. Ortiz, J. Cioslowski, and D. J. Fox, Gaussian, Inc., Wallingford CT, 2013.

Computed structures were preoptimized with a MM2 forcefield using Chem3D®. Then, optimizations were calculated at APFD/6-31G+(d,p) calculation level using Gaussian® software without any solvent correction. For the complex including bromide and iodide ions, a double basis set composed of APFD/6-31G+(d,p) for carbon, nitrogen, hydrogen, fluoride, oxygen, chloride and APFD/aug-cc-pvtz for bromide or iodine, respectively, was used. Basis sets for bromide and iodide were downloaded on basissetexchange.org. Stationary points were verified by a harmonic vibrational frequencies calculation. None of the predicated geometry has any imaginary frequency implying that the optimized geometry of each of the molecules under study lay at a minimum local point on the potential energy surface.

Effect of solvent (acetonitrile) on geometries was evaluated by proceeding optimization calculations at APFD/6-31G+(d,p) by adding the Polarizable Continuum Model (PCM) using the integral equation formalism variant (IEFPCM). For bromide and iodide, a double basis set composed of APFD/6-31G+(d,p) for carbon, nitrogen, hydrogen, fluoride, oxygen, chloride and APFD/aug-cc-pvtz for bromide and iodide, respectively, was used. Once again, stationary points were checked by a harmonic vibrational frequencies' calculation. None of the predicated geometry has any imaginary frequency implying that the optimized geometry of each of the molecules under study lay at a minimum local point on the potential energy surface.

Theoretical UV-Visible spectra were calculated on optimized geometries structures by an energy calculation using time dependant DFT calculation at TD APFD/6-311+g(d,p)//apfd/6-31+g(d,p) level and solving on 24 first singlet states. A standard solvation model (IEFPCM) for acetonitrile was used. Electrostatic Potentials Surfaces (ESP) were thus calculated using Gaussview® software from optimized structures using a fine grid for Total Density and a medium grid of ESP. NCIplots were generated from total densities using the script developed by Rzepa available on the website application of the Imperial College London. [2,3]

Distances were measured using Gaussview software on structures optimized without any solvent correction. Distances between tetrazine centroid (defined using the six atoms of tetrazine rings) and anions were measured using UCSF Chimera software. [4] Angles were measured using UCSF Chimera software. [4] Plans A and B were defined considering atoms of phenylurea (7C, 2N, 1O, 5H) and excluding -CF_3 groups. Plan of tetrazine ring was defined considering the six atoms of the tetrazine ring.

2. Computational Data of <u>2</u>, anions and <u>2</u>-anion complexes

2.1 Coordinates of computed structures

Energies reported, unless noted, are expressed in Hartree.

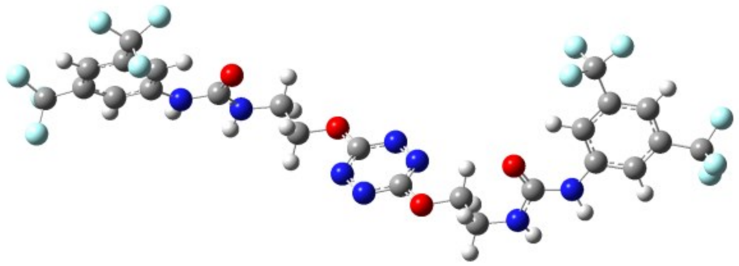

2 *(in vacuum)*

APFD/6-31+G(d,p)
Charge: 0
Spin Multiplicity: Singlet
Imaginary frequencies: 0
Electronic Energy (RAPFD) : -2860.445641 Hartree
Zero-point correction = 0.445448 (Hartree/Particle)
Thermal correction to Energy = 0.489827
Thermal correction to Enthalpy = 0.490771
Thermal correction to Gibbs Free Energy = 0.352771
Sum of electronic and zero-point Energies = -2860.000193
Sum of electronic and thermal Energies = -2859.955814
Sum of electronic and thermal Enthalpies= -2859.954870
Sum of electronic and thermal Free Energies= -2860.092870

Symbol	X	Y	Z	Symbol	X	Y	Z
C	-8.89649200	1.94314000	0.23689100	F	-7.55454600	3.81933800	-0.30632100
C	-10.21203200	1.57566800	0.49936800	F	-7.79215700	3.33455200	1.79463100
C	-10.59792600	0.27459500	0.19092000	F	-9.44679600	4.20475300	0.69099200
C	-9.69920300	-0.63293200	-0.35457600	C	-11.99663900	-0.18754900	0.50400600
C	-8.37617300	-0.24989500	-0.61199500	F	-12.44225000	-1.09285500	-0.39566000
C	-7.97291800	1.05760000	-0.31135300	F	-12.87727500	0.83048100	0.52149300
N	-7.52828900	-1.21389200	-1.16089500	F	-12.05914400	-0.78563300	1.71700700
C	-6.19941600	-1.06118100	-1.52848500	C	12.00952500	-0.16394900	-0.49550800
N	-5.62622500	-2.22119600	-1.99639600	F	12.13529000	-1.50305600	-0.62143500
O	-5.58990800	-0.00215000	-1.47063600	F	12.81546800	0.20966700	0.52532100
C	-4.22897200	-2.22163500	-2.37559200	F	12.51260400	0.39436700	-1.61429200
C	-3.31713500	-2.27515400	-1.15805000	C	8.40209500	3.31827200	-0.59395200
O	-1.98214400	-2.31529000	-1.67341700	F	7.44153300	3.29632700	-1.54438600
C	-0.98224500	-2.34274400	-0.79759700	F	9.39134500	4.11417700	-1.04617400
N	-1.24634000	-2.36761200	0.51602400	F	7.86531200	3.92002500	0.48859200
N	-0.23072900	-2.37176500	1.34125800	H	-10.91806600	2.28269800	0.91853000
C	0.98937700	-2.34910700	0.79250200	H	-10.03537400	-1.64045300	-0.58653100
N	1.25339800	-2.36850500	-0.52124300	H	-6.95810300	1.37005800	-0.51962200
N	0.23779700	-2.36605000	-1.34646500	H	-7.96902000	-2.09557400	-1.37723300
C	8.88702800	1.92703500	-0.27130700	H	-6.05930100	-3.11044200	-1.79723100
C	10.19547400	1.55700800	-0.55387300	H	-4.04337000	-3.07965800	-3.02818300
C	10.58300100	0.25277100	-0.25196200	H	-4.02581900	-1.31230900	-2.94639300
C	9.69391300	-0.64947100	0.31351000	H	-3.45480200	-1.38983000	-0.52988200
C	8.37462800	-0.26220000	0.59237400	H	-3.50566300	-3.16757500	-0.54917800
C	7.97037700	1.04424900	0.29688900	H	10.89240600	2.25773000	-0.99817500
N	7.53241800	-1.22712100	1.14836000	H	10.02778400	-1.66080900	0.53022400
C	6.20487500	-1.07639200	1.52116300	H	6.95753200	1.35776300	0.51467700
N	5.63397100	-2.23769600	1.98883300	H	7.97485400	-2.10918500	1.35952100
O	5.59455400	-0.01758600	1.46753800	H	6.06627000	-3.12621900	1.78472100
C	4.23708900	-2.23970900	2.36971500	H	4.05224400	-3.10048500	3.01886500
C	3.32429600	-2.28787900	1.15264400	H	4.03452700	-1.33278500	2.94452800
O	1.98943700	-2.32831200	1.66832400	H	3.46272400	-1.40042100	0.52768800
C	-8.42458100	3.32947400	0.59835600	H	3.51151900	-3.17830800	0.54044800

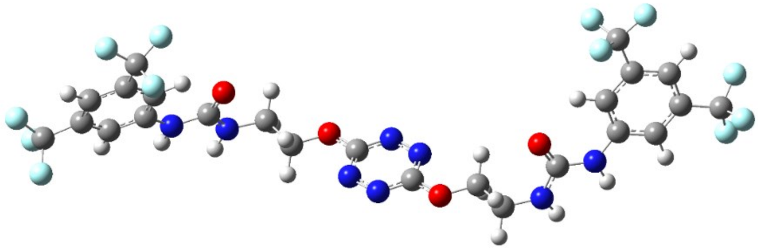

2 *(in acetonitrile)*

APFD/6-31+G(d,p), scrf=(iefpcm,solvent=acetonitrile)
Charge: 0
Spin Multiplicity: Singlet
Imaginary frequencies: 0
Electronic Energy (RAPFD) : -2860.485338 Hartree
Zero-point correction = 0.444322 (Hartree/Particle)
Thermal correction to Energy = 0.488686
Thermal correction to Enthalpy = 0.489630
Thermal correction to Gibbs Free Energy = 0.351745
Sum of electronic and zero-point Energies = -2860.041016
Sum of electronic and thermal Energies = -2859.996652
Sum of electronic and thermal Enthalpies= -2859.995708
Sum of electronic and thermal Free Energies= -2860.133594

Symbol	X	Y	Z	Symbol	X	Y	Z
C	-9.02047600	1.95043900	0.17322500	F	-7.70431700	3.81951100	-0.46412900
C	-10.33169600	1.57747600	0.44745500	F	-7.93467600	3.44173800	1.65853900
C	-10.68963200	0.25374200	0.20350100	F	-9.60140500	4.22789900	0.51242000
C	-9.77541500	-0.66778900	-0.29021200	C	-12.08296800	-0.21189900	0.52830500
C	-8.45433400	-0.27755500	-0.55972800	F	-12.52623100	-1.14644300	-0.34111600
C	-8.07877600	1.05193700	-0.32113700	F	-12.97717000	0.79784100	0.51793200
N	-7.58853800	-1.24923200	-1.05098500	F	-12.14721600	-0.77467700	1.76144000
C	-6.27142400	-1.08897600	-1.44857500	C	12.08563100	-0.22644500	-0.54500000
N	-5.68018300	-2.24619200	-1.86222100	F	12.20248400	-1.56540400	-0.65733600
O	-5.68439500	-0.00513900	-1.44691800	F	12.92560900	0.15579400	0.44945500
C	-4.29678200	-2.25881900	-2.27942500	F	12.57360600	0.31317300	-1.68403200
C	-3.36519000	-2.30291400	-1.07677600	C	8.58613200	3.35027000	-0.46778000
O	-2.03132100	-2.30990400	-1.61135900	F	7.63394200	3.41085100	-1.43078700
C	-1.01679400	-2.31507800	-0.75732800	F	9.59482500	4.15149300	-0.86409600
N	-1.24200800	-2.30555300	0.56377200	F	8.04348000	3.91322700	0.63754500
N	-0.20179600	-2.29822000	1.35685100	H	-11.05196100	2.29138600	0.82859000
C	1.00658800	-2.30185900	0.78106400	H	-10.08912500	-1.69167500	-0.47318800
N	1.23170800	-2.33129800	-0.53977000	H	-7.06614400	1.36765000	-0.53174500
N	0.19152700	-2.33754300	-1.33284500	H	-7.98819700	-2.17226700	-1.14946400
C	9.03066400	1.93512400	-0.21432600	H	-6.15481500	-3.13046100	-1.75464200
C	10.32856800	1.54387100	-0.51320500	H	-4.13530000	-3.13138400	-2.91718800
C	10.67224900	0.21273100	-0.27540500	H	-4.09493900	-1.36156100	-2.87038400
C	9.75851200	-0.69185800	0.24367700	H	-3.50594100	-1.42375000	-0.44125200
C	8.44759400	-0.28094300	0.54101600	H	-3.52491800	-3.20549800	-0.47789200
C	8.08641900	1.05118800	0.30626600	H	11.04877600	2.24474200	-0.91856000
N	7.58074900	-1.24163200	1.05122100	H	10.05889200	-1.72087400	0.41700700
C	6.26383900	-1.07199300	1.44500200	H	7.08133300	1.38133500	0.53230200
N	5.67057700	-2.21997400	1.88076600	H	7.97685000	-2.16521200	1.15832500
O	5.67862300	0.01262400	1.42158800	H	6.14508400	-3.10660100	1.79406600
C	4.28772700	-2.22164400	2.29976600	H	4.12634200	-3.07778000	2.95945200
C	3.35497300	-2.29483800	1.09948100	H	4.08738600	-1.30965900	2.86826400
O	2.02136800	-2.28097300	1.63459300	H	3.49906300	-1.43418500	0.43975100
C	-8.57163500	3.35743400	0.46311500	H	3.51013600	-3.21421600	0.52543300

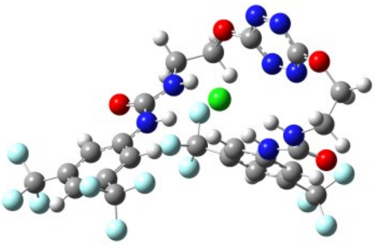

2-Cl *(in vacuum)*

APFD/6-31+G(d,p)
Charge: -1
Spin Multiplicity: Singlet
Imaginary frequencies: 0
Electronic Energy (RAPFD) : -3320.727352 Hartree
Zero-point correction = 0.448501 (Hartree/Particle)
Thermal correction to Energy = 0.492922
Thermal correction to Enthalpy = 0.493866
Thermal correction to Gibbs Free Energy = 0.360514
Sum of electronic and zero-point Energies = -3320.278851
Sum of electronic and thermal Energies = -3320.234431
Sum of electronic and thermal Enthalpies = -3320.233486
Sum of electronic and thermal Free Energies = -3320.366838

Symbol	X	Y	Z
C	-1.95835100	-2.71021700	-0.14933400
C	-3.06346500	-3.29170200	-0.77048300
C	-3.85088100	-2.47773000	-1.57497000
C	-3.57092700	-1.12727200	-1.76628400
C	-2.46006400	-0.55302000	-1.12919600
C	-1.65025300	-1.36726300	-0.31515900
N	-2.10277700	0.77440000	-1.25851200
C	-2.87461300	1.78473200	-1.80791900
N	-2.33057700	3.02645100	-1.61497900
O	-3.92800400	1.60567900	-2.41972000
C	-3.18911400	4.17316300	-1.77941800
C	-4.25646700	4.27591300	-0.69499300
O	-3.66598600	4.54537700	0.58856500
C	-3.33748800	3.53259700	1.39002700
N	-2.84233100	3.93052300	2.58066200
N	-2.37671000	3.01103300	3.36494200
C	-2.43235900	1.73704000	2.92461900
N	-3.12280700	1.33388500	1.86607700
N	-3.59018800	2.27212400	1.06400700
C	5.61796500	-0.79678900	-0.09101200
C	5.93802500	0.20319800	-0.99999600
C	4.98665700	1.19902100	-1.23075800
C	3.76330000	1.19838000	-0.58108300
C	3.45061600	0.17677600	0.33880700
C	4.39558800	-0.83183800	0.57865900
N	2.21331000	0.23806400	0.94345300
C	1.71321900	-0.58762400	1.94335500
N	0.45397500	-0.21556700	2.31489200
O	2.31256500	-1.54942800	2.41889300
C	-0.22465300	-0.87773400	3.40040900
C	-1.69333500	-0.51277200	3.36510300
O	-1.79866300	0.87866000	3.72044500
C	-1.13047100	-3.54899800	0.78168500
F	-1.07807000	-4.84438200	0.39367600
F	-1.65775000	-3.55565000	2.04021200
F	0.13343800	-3.11269800	0.89740400
C	-5.05687200	-3.04225800	-2.27354700
F	-5.20787800	-4.37017500	-2.06328300
F	-4.99693400	-2.86497500	-3.61475100
F	-6.20387000	-2.45119500	-1.86309700
C	5.32445600	2.27919100	-2.21940400
F	5.49903600	1.77895600	-3.46899000
F	6.48677000	2.90375600	-1.90212100
F	4.37752500	3.23179400	-2.30732900
C	6.59412800	-1.90869400	0.17994800
F	6.15185000	-3.09729900	-0.29659600
F	6.81144600	-2.08523900	1.50289300
F	7.80221500	-1.68979900	-0.38915200
H	-3.29252800	-4.34164800	-0.63541600
H	-4.19994500	-0.50604100	-2.39099600
H	-0.78703400	-0.94223600	0.19131600
H	-1.32919700	1.09610300	-0.66595000
H	-1.54973400	3.10121500	-0.95964900
H	-2.56363400	5.07112600	-1.77288300
H	-3.69272900	4.10834200	-2.75002000
H	-4.85508700	3.36207400	-0.65706600
H	-4.90735100	5.13559800	-0.88024100
H	6.89397800	0.21464600	-1.51019800
H	3.04006500	1.98632000	-0.77200800
H	4.16164900	-1.61796200	1.28606800
H	1.60761900	1.02129100	0.67123000
H	0.12830600	0.70904500	2.02468100
H	-0.10724000	-1.95830000	3.28746100
H	0.20140600	-0.59899200	4.37524600
H	-2.13443200	-0.68949800	2.38157700
H	-2.25579900	-1.07721400	4.11498300
Cl	-0.06463000	2.43194100	0.58226400

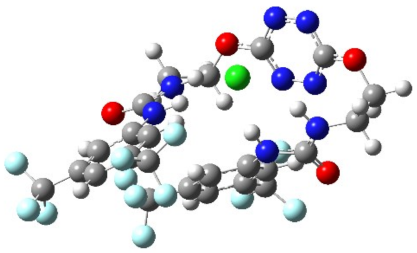

2-Cl *(in acetonitrile)*

APFD/6-31+G(d,p), scrf=(iefpcm,solvent=acetonitrile)
Charge: -1
Spin Multiplicity: Singlet
Imaginary frequencies: 0
Electronic Energy (RAPFD) : -3320.790557 Hartree
Zero-point correction = 0.446919 (Hartree/Particle)
Thermal correction to Energy = 0.491784
Thermal correction to Enthalpy = 0.492728
Thermal correction to Gibbs Free Energy = 0.358584
Sum of electronic and zero-point Energies = -3320.343638
Sum of electronic and thermal Energies = -3320.298773
Sum of electronic and thermal Enthalpies = -3320.297829
Sum of electronic and thermal Free Energies = -3320.431973

Symbol	X	Y	Z	Symbol	X	Y	Z
C	-0.52865600	-2.57269300	-0.81684800	F	0.96516200	-4.24039200	-1.57385300
C	-1.70582400	-3.31778400	-0.89481400	F	0.76394100	-3.99361400	0.56827400
C	-2.89093300	-2.63777500	-1.14658300	F	1.83746500	-2.50648300	-0.60309000
C	-2.92455000	-1.25532000	-1.31521000	C	-4.19569000	-3.38000900	-1.22780000
C	-1.73924800	-0.52104900	-1.22258200	F	-4.05749500	-4.70635600	-1.03002600
C	-0.53316300	-1.19391900	-0.97448600	F	-4.79139100	-3.22069500	-2.43441700
N	-1.70737700	0.86746300	-1.30699900	F	-5.08389300	-2.93433000	-0.30580000
C	-2.64686100	1.65108000	-1.94774200	C	4.96028500	3.15323400	-1.17667400
N	-2.55418200	2.97196300	-1.61499700	F	4.82450300	3.02659400	-2.52155700
O	-3.46152700	1.21315000	-2.76525700	F	6.24026100	3.53213300	-0.95923800
C	-3.59092400	3.89919200	-2.00776600	F	4.17305300	4.18367500	-0.80506500
C	-4.89150100	3.70026200	-1.23702300	C	6.17621400	-1.48825400	0.24812600
O	-4.66777300	3.87433000	0.18056300	F	5.65701800	-2.61977700	-0.28677200
C	-4.34345900	2.81890700	0.91872900	F	6.50829800	-1.79316200	1.52696100
N	-4.08251600	3.11061500	2.20911900	F	7.32903200	-1.23820500	-0.40535400
N	-3.60072900	2.15875600	2.94866100	H	-1.69447300	-4.39483900	-0.77172700
C	-3.41170900	0.95843600	2.36790600	H	-3.85982300	-0.74106000	-1.49609200
N	-3.90670100	0.61564000	1.18414400	H	0.39341000	-0.63206200	-0.91645900
N	-4.38224500	1.58376900	0.43015500	H	-1.09688300	1.34586100	-0.63812700
C	5.20566800	-0.34133300	0.18212800	H	-1.96589100	3.21625000	-0.82131500
C	5.56391500	0.84619100	-0.44283100	H	-3.21141500	4.91172700	-1.84811300
C	4.62051200	1.87477400	-0.46193000	H	-3.80384500	3.78327100	-3.07496900
C	3.37036000	1.72544900	0.11835900	H	-5.31892000	2.71470100	-1.42807600
C	3.02223900	0.51652800	0.74898600	H	-5.61590700	4.47332800	-1.50025500
C	3.95821000	-0.52560300	0.77590300	H	6.53981200	0.97156800	-0.89672200
N	1.75140300	0.43391400	1.28834100	H	2.65451500	2.54172200	0.08990300
C	1.19318600	-0.62101600	1.99401700	H	3.69800400	-1.46175700	1.25187700
N	-0.11590100	-0.41005600	2.28446700	H	1.14185500	1.24347100	1.13602700
O	1.80416700	-1.64632700	2.30682900	H	-0.51929600	0.50510700	2.09523400
C	-0.92211100	-1.41070700	2.94163200	H	-0.60817500	-2.40208300	2.60132600
C	-2.37260900	-1.19477800	2.57582500	H	-0.79868900	-1.37886300	4.03255400
O	-2.74699400	0.09110200	3.12120800	H	-2.51630200	-1.19855200	1.49294000
C	0.76182900	-3.31393400	-0.60117900	H	-3.01915000	-1.94932700	3.03070500
				Cl	-0.57286700	2.66148600	1.06087500

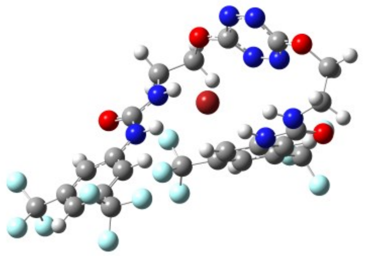

2-Br *(in vacuum)*

APFD/6-31+G(d,p) for C, N, F, H, O and APFD/aug-cc-pvtz for Br
Charge: -1
Spin Multiplicity: Singlet
Imaginary frequencies: 0
Electronic Energy (RAPFD) : -2873.804499 Hartree
Zero-point correction = 0.447846 (Hartree/Particle)
Thermal correction to Energy = 0.492801
Thermal correction to Enthalpy = 0.493745
Thermal correction to Gibbs Free Energy = 0.357953
Sum of electronic and zero-point Energies = -2873.356653
Sum of electronic and thermal Energies = -2873.311698
Sum of electronic and thermal Enthalpies = -2873.310754
Sum of electronic and thermal Free Energies = -2873.446547

Symbol	X	Y	Z	Symbol	X	Y	Z
C	-1.40786000	-2.75368600	-0.48570200	F	0.02001900	-4.63724400	-0.59355300
C	-2.56254700	-3.50420200	-0.70987300	F	-0.32886200	-3.76928400	1.36067500
C	-3.68463000	-2.83776500	-1.18814000	F	0.93068800	-2.73366300	-0.07792200
C	-3.68017200	-1.47024600	-1.45300900	C	-4.94001200	-3.60911400	-1.48913400
C	-2.51386100	-0.72805700	-1.21791800	F	-4.95388100	-4.82731000	-0.89974700
C	-1.37337700	-1.38745300	-0.72082000	F	-5.09346800	-3.82533600	-2.81985100
N	-2.40591100	0.63087000	-1.43629400	F	-6.05146800	-2.96118800	-1.07797300
C	-3.39136900	1.48584600	-1.89191900	C	5.44568400	2.42737300	-2.04026800
N	-3.02925900	2.80491000	-1.78726000	F	6.07047100	1.97749100	-3.15629800
O	-4.46785000	1.12590100	-2.36700800	F	6.30229100	3.30745300	-1.46069600
C	-4.05591600	3.81053900	-1.90568200	F	4.37474300	3.13554300	-2.44497700
C	-5.03294900	3.80452100	-0.73414600	C	6.73183600	-1.75482600	0.37036500
O	-4.36602200	4.14772500	0.49255900	F	6.34370700	-2.95590100	-0.12097200
C	-3.87881000	3.17500300	1.26343500	F	6.90230300	-1.92416800	1.70116300
N	-3.32652700	3.61995100	2.41208400	F	7.95420100	-1.50058900	-0.15133500
N	-2.71513300	2.74854800	3.14950500	H	-2.57879300	-4.57183700	-0.52428000
C	-2.69157600	1.47342300	2.70858800	H	-4.56010500	-0.96666600	-1.83281600
N	-3.43116800	1.00519200	1.71203800	H	-0.46555400	-0.82406300	-0.52549400
N	-4.04222500	1.89612900	0.95420100	H	-1.59396800	1.08224600	-1.00229800
C	5.73477200	-0.67202300	0.05972100	H	-2.20695600	3.02866100	-1.22914300
C	6.06231500	0.33985000	-0.83418200	H	-3.56643500	4.78656600	-1.97993000
C	5.08979700	1.30350200	-1.10780800	H	-4.62412000	3.63749900	-2.82629700
C	3.83983700	1.26341400	-0.51051200	H	-5.51740500	2.82891900	-0.64574800
C	3.52073700	0.23012900	0.39318800	H	-5.78989800	4.58398700	-0.86367300
C	4.48596500	-0.74817600	0.67457200	H	7.03726200	0.37990100	-1.30546500
N	2.25545500	0.24731400	0.94214000	H	3.09892400	2.02585600	-0.73679600
C	1.73311400	-0.60805500	1.90640000	H	4.24534600	-1.54478200	1.36798500
N	0.44051500	-0.29963800	2.20500200	H	1.63868000	1.00032300	0.62808700
O	2.34801100	-1.54395200	2.41268100	H	0.06854900	0.59729300	1.89389800
C	-0.28810000	-1.02453100	3.21504400	H	-0.12104800	-2.09574400	3.07652500
C	-1.75944500	-0.70768200	3.06666900	H	0.05068600	-0.76233100	4.22784000
O	-1.91948900	0.67304100	3.43761800	H	-2.10164000	-0.86791300	2.04066400
C	-0.19079900	-3.45966500	0.04478900	H	-2.37133600	-1.30980700	3.74650300
				Br	-0.25748900	2.49532200	0.30900400

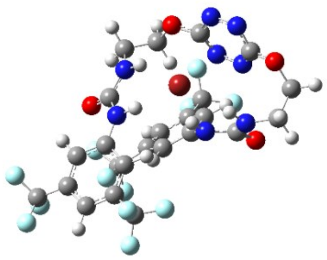

2-Br *(in acetonitrile)*

APFD/6-31+G(d,p) for C, N, F, H, O and APFD/aug-cc-pvtz for Br, scrf=(iefpcm,solvent=acetonitrile)
Charge: -1
Spin Multiplicity: Singlet
Imaginary frequencies: 0
Electronic Energy (RAPFD) : -3277.577175 Hartree
Zero-point correction = 0.446812 (Hartree/Particle)
Thermal correction to Energy = 0.491786
Thermal correction to Enthalpy = 0.492730
Thermal correction to Gibbs Free Energy = 0.359160
Sum of electronic and zero-point Energies = -3277.130364
Sum of electronic and thermal Energies = -3277.085389
Sum of electronic and thermal Enthalpies = -3277.084445
Sum of electronic and thermal Free Energies = -3277.218016

Symbol	X	Y	Z	Symbol	X	Y	Z
C	-0.55183700	2.47107000	-1.02669000	F	-2.32264400	3.57075100	-2.13106100
C	0.46497400	3.42198800	-1.08321200	F	-2.16294400	3.87605700	0.00635900
C	1.77194500	2.97735400	-1.25589900	F	-2.86063300	1.96827500	-0.77322000
C	2.07339500	1.62269100	-1.36337200	C	2.90944500	3.96004000	-1.25589800
C	1.04501100	0.67999900	-1.28556200	F	2.50416400	5.22565800	-1.48852000
C	-0.27623200	1.11179500	-1.12448400	F	3.84151800	3.66201700	-2.18838500
N	1.30569400	-0.69514700	-1.31959600	F	3.55622000	3.97285200	-0.06188400
C	2.29793700	-1.26918000	-2.09017700	C	-3.85309900	-3.18062300	-1.59622600
N	2.58737000	-2.55355500	-1.73077400	F	-4.76521200	-2.98142100	-2.57161400
O	2.83453100	-0.69216200	-3.04106700	F	-4.18966100	-4.34602000	-0.98638200
C	3.72409700	-3.23552400	-2.30599300	F	-2.66450000	-3.39592400	-2.20108000
C	5.06235200	-2.71419700	-1.79442800	C	-6.06207900	0.61929800	0.80588800
O	5.15800000	-2.87944800	-0.36183500	F	-5.94670200	1.81601900	0.17751300
C	4.77205400	-1.88631500	0.43084100	F	-6.22485300	0.89493500	2.12001600
N	4.90826800	-2.14765800	1.74689100	F	-7.21447900	0.06582800	0.37268100
N	4.40663000	-1.28862500	2.58060900	H	0.24248000	4.48075800	-1.00778100
C	3.80430200	-0.20069200	2.06239800	H	3.09665100	1.29033500	-1.48464800
N	3.89898400	0.16785100	0.79020900	H	-1.08095300	0.38482100	-1.09380100
N	4.38883900	-0.71091000	-0.05663200	H	0.95711100	-1.24486800	-0.53266600
C	-4.86826700	-0.26049300	0.55531400	H	2.24113500	-2.87868200	-0.82973600
C	-4.94290900	-1.27295600	-0.39699300	H	3.62555100	-4.30033700	-2.07818100
C	-3.80007200	-2.03725200	-0.62134700	H	3.70745500	-3.12111700	-3.39445100
C	-2.61781300	-1.79599000	0.06820800	H	5.20498900	-1.66479400	-2.05730200
C	-2.55463000	-0.76177800	1.01456100	H	5.88380900	-3.31019300	-2.19742400
C	-3.70118900	0.00362800	1.26456100	H	-5.85877700	-1.46195300	-0.94436300
N	-1.35203200	-0.56282400	1.67514400	H	-1.73604300	-2.39963600	-0.12950600
C	-0.89892000	0.63525100	2.20562600	H	-3.66182400	0.80554900	1.98991800
N	0.34012900	0.52403500	2.75583500	H	-0.65505600	-1.30967100	1.58278800
O	-1.55074700	1.68261900	2.18209200	H	0.82032100	-0.37069100	2.70111500
C	1.09113900	1.67292800	3.20258500	H	0.52275200	2.57180100	2.95040500
C	2.43929500	1.71782300	2.51639500	H	1.22392400	1.65163900	4.29153500
O	3.16371500	0.54475700	2.95387400	H	2.33080600	1.70796600	1.43042700
C	-1.97309400	2.95761500	-0.96580900	H	3.02026400	2.59429200	2.81706400
				Br	1.25134100	-2.63381800	1.4513930

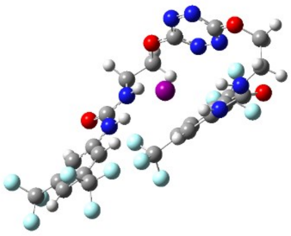

2-I *(in vacuum)*

APFD/6-31+G(d,p) for C, N, F, H, O, Cl and APFD/aug-cc-pvtz for I
Charge: -1
Spin Multiplicity: Singlet
Imaginary frequencies: 0
Electronic Energy (RAPFD) : -2872.023258 Hartree
Zero-point correction = 0.447287 (Hartree/Particle)
Thermal correction to Energy = 0.492424
Thermal correction to Enthalpy = 0.493369
Thermal correction to Gibbs Free Energy = 0.357815
Sum of electronic and zero-point Energies = -2871.575971
Sum of electronic and thermal Energies = -2871.530834
Sum of electronic and thermal Enthalpies = -2871.529890
Sum of electronic and thermal Free Energies = -2871.665443

Symbol	X	Y	Z	Symbol	X	Y	Z
C	1.09001200	2.61395200	-1.00901200	F	-0.44378600	3.93510200	0.21670700
C	2.20648900	3.42251900	-0.79543100	F	-1.27350900	2.36170500	-1.03626300
C	3.46457400	2.84175000	-0.91834800	F	-0.44198400	4.15265000	-1.93849200
C	3.63223900	1.50018700	-1.25301300	C	4.69865400	3.67829500	-0.72243900
C	2.50131700	0.69973900	-1.45602200	F	4.43008200	4.86661200	-0.13269600
C	1.22296700	1.27161200	-1.32893100	F	5.30871200	3.96165800	-1.89997200
N	2.55661600	-0.65592300	-1.72770300	F	5.62067500	3.05855500	0.04552500
C	3.69401000	-1.40721600	-1.96731700	C	-5.69714200	-2.69228000	-1.35037300
N	3.48576000	-2.74583700	-1.75256600	F	-6.37057000	-2.43524400	-2.49929700
O	4.76188300	-0.94538800	-2.36192000	F	-6.54273200	-3.42062300	-0.57685900
C	4.61518000	-3.64208300	-1.74473700	F	-4.66691200	-3.49739200	-1.66799600
C	5.54311600	-3.43453900	-0.55059700	C	-6.84109100	1.80038100	0.47060000
O	4.85011400	-3.67474500	0.68703500	F	-6.30356700	3.03638800	0.48639900
C	4.28608000	-2.64188200	1.31517300	F	-7.44290300	1.62484300	1.67514300
N	3.69509100	-2.96162500	2.48630400	F	-7.83471500	1.81224600	-0.45000300
N	3.00096300	-2.03530100	3.06923700	H	2.09520000	4.47098100	-0.54348100
C	2.94065600	-0.83273500	2.46014400	H	4.61846900	1.06099200	-1.33896600
N	3.72752400	-0.45701900	1.46041800	H	0.34179400	0.65380400	-1.47148600
N	4.41734400	-1.40501700	0.85737600	H	1.73118900	-1.17990000	-1.42556800
C	-5.82300700	0.72782000	0.19502600	H	2.66191900	-3.01614700	-1.21876200
C	-6.22218700	-0.42924100	-0.46478400	H	4.22920600	-4.66614200	-1.74634500
C	-5.26730900	-1.42710600	-0.66154800	H	5.19999900	-3.49249000	-2.65938700
C	-3.96338200	-1.28325000	-0.21165200	H	5.96119900	-2.42542400	-0.56367100
C	-3.57331900	-0.10609600	0.45571600	H	6.35051500	-4.17274400	-0.56464900
C	-4.52239900	0.90821700	0.65919700	H	-7.23786300	-0.54878300	-0.82466200
N	-2.25700700	-0.01867500	0.86025300	H	-3.23579200	-2.07393800	-0.37637100
C	-1.65968900	0.99783100	1.59577300	H	-4.22513300	1.81639200	1.16787500
N	-0.32097100	0.79877300	1.75031000	H	-1.65360700	-0.79854100	0.59347400
O	-2.25594900	1.97641000	2.04016700	H	0.06701900	-0.11668400	1.52759200
C	0.46375800	1.68526800	2.57421000	H	0.30722700	2.71777400	2.24712900
C	1.92221700	1.32269900	2.42381600	H	0.16402400	1.62448600	3.62992800
O	2.08151500	0.01846100	3.01094800	H	2.22224400	1.30231400	1.37281200
C	-0.27097300	3.25016000	-0.93584600	H	2.56994000	2.02350800	2.96102100
				I	0.32454700	-2.52576500	0.23624300

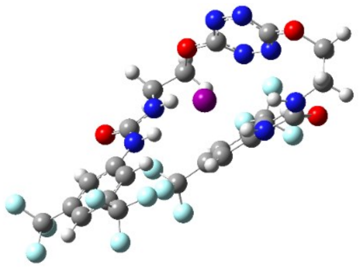

2-I *(in acetonitrile)*

APFD/6-31+G(d,p) for C, N, F, H, O, Cl and APFD/aug-cc-pvtz for I
Charge: -1
Spin Multiplicity: Singlet
Imaginary frequencies: 0
Electronic Energy (RAPFD) : -3156.461982 Hartree
Zero-point correction = 0.445969 (Hartree/Particle)
Thermal correction to Energy = 0.491373
Thermal correction to Enthalpy = 0.492317
Thermal correction to Gibbs Free Energy = 0.355340
Sum of electronic and zero-point Energies = -3156.016013
Sum of electronic and thermal Energies = -3155.970609
Sum of electronic and thermal Enthalpies = -3155.969665
Sum of electronic and thermal Free Energies = -3156.106642

Symbol	X	Y	Z	Symbol	X	Y	Z
C	0.89507500	2.60792900	-1.04927200	F	-0.67300200	3.91687800	0.14591600
C	1.98635400	3.44745400	-0.82537900	F	-1.45993100	2.27929700	-1.04832500
C	3.25872900	2.89820500	-0.93544300	F	-0.69643600	4.06557400	-2.01547000
C	3.46188300	1.55903700	-1.26059500	C	4.47472000	3.75497200	-0.71439500
C	2.35608600	0.72815300	-1.47248900	F	4.17505100	4.95910000	-0.18642100
C	1.06361900	1.26870900	-1.36840600	F	5.14087100	3.98960300	-1.87237200
N	2.45641500	-0.63316800	-1.72195000	F	5.36246400	3.16623900	0.12063000
C	3.61308300	-1.33727200	-2.00776100	C	-5.54631500	-2.74636000	-1.38893500
N	3.49060200	-2.67493100	-1.76696100	F	-6.22648500	-2.48566900	-2.53003000
O	4.63486200	-0.81971500	-2.46407400	F	-6.36163300	-3.52230600	-0.63122200
C	4.64464500	-3.54364800	-1.83120400	F	-4.48679100	-3.50825000	-1.72561800
C	5.62101500	-3.33162600	-0.67869600	C	-6.79315600	1.69885300	0.50512900
O	4.96768700	-3.56223800	0.58943000	F	-6.27532900	2.94594200	0.45609800
C	4.41149200	-2.53776900	1.22625800	F	-7.33013200	1.55675800	1.74428100
N	3.80750700	-2.87306200	2.38529800	F	-7.82392400	1.66356400	-0.36595800
N	3.10829600	-1.95518300	2.97896000	H	1.84655800	4.49221600	-0.57225200
C	3.05411200	-0.74221600	2.39559900	H	4.46131600	1.14851200	-1.32614600
N	3.84396400	-0.35437700	1.40039200	H	0.19868400	0.63320300	-1.53049400
N	4.53946700	-1.28981700	0.78853400	H	1.66051300	-1.18437600	-1.39739400
C	-5.76285000	0.63982800	0.22143900	H	2.68853400	-2.99007700	-1.22785600
C	-6.13369700	-0.50932000	-0.46646800	H	4.28364900	-4.57535700	-1.83375800
C	-5.15627700	-1.48464900	-0.66945000	H	5.18176800	-3.37211800	-2.76942100
C	-3.86197700	-1.32595900	-0.19873500	H	6.04717800	-2.32753400	-0.70000800
C	-3.50148700	-0.15732300	0.49717900	H	6.42171100	-4.07288100	-0.71825400
C	-4.47094800	0.83324000	0.70725900	H	-7.14322300	-0.64065700	-0.83945400
N	-2.18690700	-0.05617300	0.91599400	H	-3.11981400	-2.10015400	-0.37137100
C	-1.60074100	0.96579400	1.64536700	H	-4.20337000	1.73757200	1.23712700
N	-0.25532600	0.82030000	1.75819300	H	-1.56960000	-0.81671000	0.62831600
O	-2.22323900	1.91625300	2.12826100	H	0.16706200	-0.06241500	1.47669200
C	0.53514600	1.73743400	2.54784300	H	0.36454400	2.76250200	2.20434600
C	1.99650700	1.40035100	2.37464800	H	0.25848200	1.69254300	3.60942600
O	2.18335500	0.09025600	2.95114800	H	2.28295400	1.38640600	1.32006600
C	-0.48362500	3.20416500	-0.98764700	H	2.63670500	2.10797400	2.90821600
				I	0.39502000	-2.58695600	0.39011800

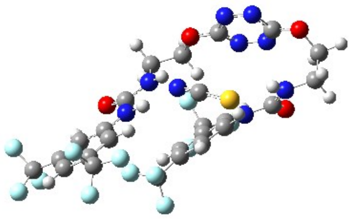

2-SCN *(in vacuum)*

APFD/6-31+G(d,p)
Charge: -1
Spin Multiplicity: Singlet
Imaginary frequencies: 0
Electronic Energy (RAPFD) : -3351.485046 Hartree
Zero-point correction = 0.457131 (Hartree/Particle)
Thermal correction to Energy = 0.504183
Thermal correction to Enthalpy = 0.505127
Thermal correction to Gibbs Free Energy = 0.364665
Sum of electronic and zero-point Energies = -3351.027915
Sum of electronic and thermal Energies = -3350.980863
Sum of electronic and thermal Enthalpies = -3350.979919
Sum of electronic and thermal Free Energies = -3351.120381

Symbol	X	Y	Z	Symbol	X	Y	Z
C	-0.16375300	1.25553400	-1.68640400	F	-1.80634500	1.49563000	-3.36119300
C	0.31333500	2.53739900	-1.42008600	C	2.21219800	4.05261500	-0.85024500
C	1.67871500	2.69324800	-1.20586900	F	1.47394000	5.05271700	-1.38144000
C	2.56120000	1.61512700	-1.23887900	F	3.48347700	4.23625900	-1.25940000
C	2.05828700	0.32901400	-1.47260600	F	2.20958400	4.25343500	0.49639900
C	0.68434700	0.15898600	-1.70284200	C	-5.23045000	-3.28043000	-0.49172300
N	2.85075000	-0.80651700	-1.44579000	F	-6.00704700	-3.22260200	-1.60178200
C	4.21765600	-0.87146000	-1.64290400	F	-5.95640900	-3.95700600	0.43658300
N	4.72068900	-2.11205400	-1.35487100	F	-4.16878500	-4.05194400	-0.79217200
O	4.91622500	0.06273200	-2.02744800	C	-6.53993900	1.36691800	0.77607700
C	6.11092500	-2.41777500	-1.57677600	F	-6.15476100	2.55743200	0.26669200
C	7.02985000	-1.77579700	-0.53799600	F	-6.81639300	1.58386400	2.08669600
O	6.62838100	-2.17811500	0.78236800	F	-7.71512800	1.04856800	0.18460300
C	5.70039900	-1.42896400	1.38644100	H	-0.36065400	3.38591000	-1.39123100
N	5.02529500	-2.05042600	2.37338300	H	3.62366000	1.75605400	-1.08135000
N	4.02786700	-1.40918100	2.90594000	H	0.29051400	-0.83420300	-1.89438600
C	3.75347000	-0.18356500	2.41669600	H	2.36939900	-1.70442600	-1.33997100
N	4.55921100	0.50723600	1.61270100	H	4.09072500	-2.82927500	-1.00497100
N	5.57606900	-0.14163700	1.08558200	H	6.21714900	-3.50608500	-1.54592800
C	-5.49346600	0.30255200	0.59433600	H	6.42123400	-2.06657100	-2.56923500
C	-5.85333300	-0.95160300	0.11740600	H	7.01052200	-0.68789500	-0.62670000
C	-4.84714000	-1.91132800	-0.00582000	H	8.05439800	-2.14007700	-0.65148700
C	-3.53389800	-1.63289100	0.33665100	H	-6.87656800	-1.17721100	-0.15876800
C	-3.18211300	-0.35622000	0.81480600	H	-2.76601300	-2.39245600	0.22332500
C	-4.18236800	0.61745900	0.94461800	H	-3.91460100	1.60463100	1.29940200
N	-1.85177400	-0.14113000	1.10534800	H	-1.21206300	-0.93561800	0.98249400
C	-1.28039200	0.99118100	1.66711500	H	0.43976800	-0.11914400	1.82430700
N	0.05002500	0.81890200	1.90926400	H	0.47191200	2.81001000	2.28734500
O	-1.88659300	2.03526600	1.90810600	H	0.59670100	1.76927400	3.71163500
C	0.79101500	1.82131000	2.62960700	H	2.47069100	1.68996400	1.28710500
C	2.26376700	1.65299600	2.35787900	H	2.86383000	2.41984100	2.85912700
O	2.62945300	0.35684000	2.86898300	S	1.74317500	-3.81782500	-0.68590200
C	-1.60764200	1.09404000	-2.07559900	C	0.91907100	-2.78358600	0.30068400
F	-2.43040300	1.84119200	-1.31589200	N	0.32769200	-2.01230400	0.96939200
F	-2.02954500	-0.18311100	-2.00646900				

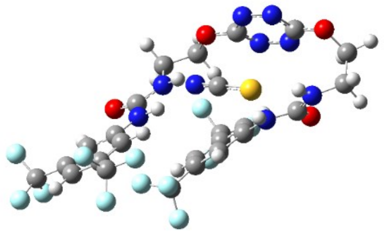

2-SCN *(in acetonitrile)*

APFD/6-31+G(d,p), scrf=(iefpcm,solvent=acetonitrile)
Charge: -1
Spin Multiplicity: Singlet
Imaginary frequencies: 0
Electronic Energy (RAPFD) : -3351.548334 Hartree
Zero-point correction = 0.456095 (Hartree/Particle)
Thermal correction to Energy = 0.503354
Thermal correction to Enthalpy = 0.504298
Thermal correction to Gibbs Free Energy = 0.364811
Sum of electronic and zero-point Energies = -3351.092239
Sum of electronic and thermal Energies = -3351.044980
Sum of electronic and thermal Enthalpies = -3351.044036
Sum of electronic and thermal Free Energies = -3351.183523

Symbol	X	Y	Z	Symbol	X	Y	Z
C	-0.18437800	1.34777900	-1.67530600	F	-1.80860400	1.65480000	-3.36072600
C	0.30361800	2.61810200	-1.37814000	C	2.21093700	4.10037700	-0.75468400
C	1.66639100	2.75029300	-1.13189700	F	1.57031400	5.10749000	-1.38645000
C	2.53143500	1.65995100	-1.16608500	F	3.52456400	4.22527700	-1.03420000
C	2.02078500	0.38770200	-1.45128200	F	2.07624500	4.33885800	0.57706900
C	0.65067800	0.23992000	-1.70789900	C	-5.03877600	-3.34195500	-0.58704500
N	2.81235500	-0.75227000	-1.45721700	F	-5.68884100	-3.28961100	-1.77491200
C	4.17167600	-0.81679700	-1.71841300	F	-5.85017700	-4.02731700	0.25679300
N	4.67979000	-2.06506200	-1.52764800	F	-3.93989500	-4.09963600	-0.77349900
O	4.84681600	0.14648000	-2.08521700	C	-6.55744500	1.22410900	0.75830900
C	6.08113800	-2.36273400	-1.70968000	F	-6.24232200	2.41265200	0.19069700
C	6.94763400	-1.85336400	-0.55827200	F	-6.77985100	1.48017100	2.07219100
O	6.40035600	-2.31100600	0.69713800	F	-7.73819200	0.83827100	0.23270200
C	5.49397300	-1.54099600	1.29476000	H	-0.35625700	3.47828900	-1.35528400
N	4.73330300	-2.18058100	2.20589200	H	3.58941400	1.78467800	-0.97560100
N	3.77331600	-1.50589300	2.76440300	H	0.24875100	-0.73962100	-1.94636400
C	3.61456400	-0.22352800	2.38500200	H	2.33097100	-1.64108400	-1.34934500
N	4.46418200	0.44887600	1.61265300	H	4.07304400	-2.78283000	-1.14213100
N	5.43926500	-0.23448600	1.05337600	H	6.17425500	-3.44773800	-1.79781400
C	-5.47056500	0.20302100	0.56626700	H	6.44323800	-1.91447500	-2.64069600
C	-5.77497100	-1.05867600	0.07356900	H	7.01283900	-0.76513900	-0.55910300
C	-4.72412100	-1.96841100	-0.06360700	H	7.95018200	-2.28121800	-0.60976700
C	-3.42436200	-1.63363900	0.27799500	H	-6.78914000	-1.32725600	-0.19878900
C	-3.13029400	-0.34905700	0.77152600	H	-2.62578500	-2.35813200	0.15294100
C	-4.17309600	0.57332900	0.91711000	H	-3.95602600	1.56601500	1.28838700
N	-1.80626400	-0.07469200	1.05676800	H	-1.14053800	-0.84137700	0.92550200
C	-1.28129000	1.05464800	1.66486900	H	0.47955300	0.02558200	1.74396300
N	0.05569200	0.94357900	1.87535200	H	0.49290300	2.92062300	2.36556600
O	-1.94219800	2.05385800	1.96828300	H	0.51002500	1.81223500	3.74419200
C	0.76550500	1.90940200	2.67957000	H	2.51566100	1.83092600	1.42525600
C	2.24764000	1.71826600	2.47645800	H	2.83615500	2.41747800	3.07701900
O	2.54436400	0.36911300	2.89649500	S	1.93252800	-3.88398600	-0.33183200
C	-1.62376100	1.21316500	-2.08776100	C	1.09300100	-2.72876000	0.49273900
F	-2.44983200	1.94614900	-1.31252200	N	0.49130100	-1.88475900	1.05687200
F	-2.06235300	-0.06005900	-2.05869700				

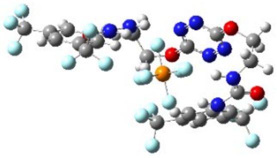

2-PF6 *(in vacuum)*

APFD/6-31+G(d,p)
Charge: -1
Spin Multiplicity: Singlet
Imaginary frequencies: 0
Electronic Energy (RAPFD) : -3800.744433 Hartree
Zero-point correction = 0.469017 (Hartree/Particle)
Thermal correction to Energy = 0.519567
Thermal correction to Enthalpy = 0.520511
Thermal correction to Gibbs Free Energy = 0.372854
Sum of electronic and zero-point Energies = -3800.275415
Sum of electronic and thermal Energies = -3800.224866
Sum of electronic and thermal Enthalpies = -3800.223922
Sum of electronic and thermal Free Energies = -3800.371578

Symbol	X	Y	Z	Symbol	X	Y	Z
C	6.93089800	0.00807900	-0.55277700	F	-2.75025700	-4.23058300	-0.65710900
C	5.93062800	0.96498100	-0.71051000	F	-1.37749400	-2.94452900	-1.74560700
C	4.68427500	0.81873700	-0.11589700	F	-3.00653900	-3.93098600	-2.78819500
C	4.40990700	-0.30998800	0.67438400	C	6.22933900	2.21422400	-1.49220900
C	5.40471700	-1.28641400	0.83619900	F	8.93364300	-1.73080100	0.18994900
C	6.64152500	-1.11025900	0.22295900	F	7.50051000	-3.17712000	-0.55862200
N	3.17115200	-0.37882900	1.28901300	F	7.63620100	-2.79370200	1.57126800
C	2.54919900	-1.52960200	1.76141800	C	-2.67245400	-3.29470400	-1.63593600
N	1.34981300	-1.26829800	2.36198000	F	7.12859300	1.99430500	-2.48065600
O	3.03758200	-2.65400200	1.66991200	F	6.76644700	3.18057100	-0.70089100
C	0.36988100	-2.31717500	2.51020800	F	5.13318400	2.74710300	-2.06133100
C	-0.73128100	-2.26579300	1.44203100	H	7.89982900	0.12971800	-1.02224100
O	-1.98688000	-1.68179600	1.84908200	H	3.91217200	1.56424100	-0.28230400
C	-2.07449100	-0.37580700	2.08580100	H	5.19426800	-2.17034500	1.42397200
N	-3.27718200	0.15499700	1.83956000	H	2.55827500	0.41217700	1.12292300
N	-3.38433100	1.45131400	1.96170200	H	0.97157700	-0.32754100	2.36391000
C	-2.30179100	2.14135700	2.33619700	H	-0.07958000	-2.28315500	3.50870000
N	-1.16765700	1.58999200	2.78105200	H	0.91263100	-3.26168300	2.41872700
N	-1.05663900	0.29524400	2.63654700	H	-1.03253200	-3.27424600	1.15534500
C	-4.88285500	-2.37880600	-0.95378000	H	-0.37297400	-1.73909800	0.55266300
C	-3.57594300	-2.12564100	-1.36570600	H	-5.23245700	-3.39288100	-0.79836900
C	-3.10912400	-0.83237000	-1.54344600	H	-2.07710600	-0.65989400	-1.83217400
C	-3.96157500	0.25793400	-1.30447400	H	-5.93915800	0.86087000	-0.71669200
C	-5.28407400	0.02173000	-0.91242900	H	-2.43230800	1.53030100	-1.66072700
C	-5.71646500	-1.28924900	-0.73430600	H	-1.92563900	3.26114100	-0.53285900
N	-3.42096000	1.53227600	-1.44179300	H	-4.06869500	5.18747600	0.06778100
C	-3.88796400	2.66170400	-0.77695000	H	-2.32989200	5.32474600	0.40933400
N	-2.89515900	3.55156800	-0.46943700	H	-4.35021700	3.54143200	1.88131400
O	-5.06842700	2.85699800	-0.50141700	H	-3.59746700	5.03547400	2.51813800
C	-3.18674300	4.64495700	0.42601500	P	0.41024600	1.39385800	-0.79357300
C	-3.46311100	4.17698600	1.85356100	F	-0.99734900	1.00256200	-0.03108300
O	-2.32295900	3.47342200	2.37949900	F	1.78266300	1.79924800	-1.53940400
C	7.67291100	-2.19575900	0.36091500	F	0.81760400	-0.17795800	-0.82429500
F	-7.60068000	-0.59951500	0.53537500	F	1.14075300	1.55544400	0.67355900
F	-7.34015400	-2.73396200	0.22502500	F	-0.37463700	1.24672100	-2.22349300
F	-7.97977400	-1.46558600	-1.41552000	F	-0.03842100	2.97252300	-0.72278500
C	-7.14888500	-1.51995800	-0.34068100				

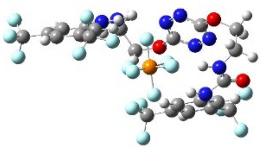

2-PF6 *(in acetonitrile)*

APFD/6-31+G(d,p), scrf=(iefpcm,solvent=acetonitrile)
Charge: -1
Spin Multiplicity: Singlet
Imaginary frequencies: 0
Electronic Energy (RAPFD) : -3800.804832 Hartree
Zero-point correction = 0.467451 (Hartree/Particle)
Thermal correction to Energy = 0.518317
Thermal correction to Enthalpy = 0.519261
Thermal correction to Gibbs Free Energy = 0.371186
Sum of electronic and zero-point Energies = -3800.337381
Sum of electronic and thermal Energies = -3800.286515
Sum of electronic and thermal Enthalpies = -3800.285571
Sum of electronic and thermal Free Energies = -3800.433646

Symbol	X	Y	Z	Symbol	X	Y	Z
C	6.64594300	0.01222400	-0.60908500	F	-2.92435000	-4.26044900	-0.42491600
C	5.70742800	1.03781700	-0.49750500	F	-1.45315100	-3.08044200	-1.50374100
C	4.54223000	0.88055900	0.23829500	F	-3.08633100	-4.02794500	-2.57442300
C	4.28321700	-0.33307900	0.89584200	C	5.99702200	2.35727100	-1.15769500
C	5.21919400	-1.37018300	0.80119700	F	8.53943300	-1.96567900	-0.55058300
C	6.37836600	-1.18100600	0.05071600	F	6.86278400	-3.30223100	-0.88774800
N	3.11631700	-0.42356200	1.64443900	F	7.55956000	-2.93055900	1.13113100
C	2.43934300	-1.59120300	1.96845600	C	-2.76858900	-3.36907500	-1.43209700
N	1.30443400	-1.36552500	2.69064800	F	6.57198200	2.20159300	-2.37183200
O	2.84522900	-2.71499300	1.66206000	F	6.85896800	3.10597600	-0.42206200
C	0.27705400	-2.38127200	2.76468000	F	4.89053500	3.10655400	-1.33512700
C	-0.79641800	-2.24471100	1.68202200	H	7.55117300	0.14214500	-1.19057400
O	-1.98455200	-1.50964900	2.05767800	H	3.82076500	1.68856400	0.29088400
C	-1.96081000	-0.18672300	2.17713400	H	5.03330600	-2.30977700	1.30422200
N	-3.12656800	0.41234200	1.90714700	H	2.56410300	0.42396100	1.66678500
N	-3.14216300	1.71705500	1.91116000	H	0.94255200	-0.41913000	2.77270200
C	-2.00206400	2.36041500	2.19250600	H	-0.18706300	-2.37023000	3.75599800
N	-0.89818100	1.76433400	2.65701000	H	0.76914000	-3.34845800	2.64104900
N	-0.88369400	0.45720800	2.63796600	H	-1.20291900	-3.22570700	1.43437500
C	-4.95037900	-2.31752600	-0.84392100	H	-0.37834400	-1.79176700	0.77891500
C	-3.62315400	-2.14821800	-1.23758300	H	-5.35671300	-3.30392500	-0.65217800
C	-3.08868900	-0.89064900	-1.46863400	H	-2.04669300	-0.78577600	-1.75080800
C	-3.88745600	0.25286400	-1.30417100	H	-5.85190600	0.97699000	-0.81156700
C	-5.22834100	0.10149500	-0.93631000	H	-2.29252700	1.41669400	-1.72347800
C	-5.73096700	-1.17700600	-0.70173500	H	-1.68555400	3.21166100	-0.80317300
N	-3.27707700	1.48792500	-1.50257500	H	-3.70182200	5.28509300	-0.26080600
C	-3.67183100	2.67999700	-0.90307700	H	-1.94825800	5.36645000	-0.03466800
N	-2.63694100	3.53712600	-0.68187000	H	-3.96430500	3.88623000	1.75125000
O	-4.84023400	2.94182600	-0.61501600	H	-3.04832500	5.36216700	2.16537400
C	-2.82902500	4.73493900	0.10453800	P	0.58721000	1.12226600	-0.92517300
C	-3.03741600	4.43649000	1.58659600	F	-0.82643000	0.93086900	-0.11008700
O	-1.91858200	3.68260100	2.10649800	F	1.96632500	1.33327100	-1.74906900
C	7.33688100	-2.33440900	-0.06314100	F	0.98216300	-0.42129100	-0.60461000
F	-7.56423300	-0.36120000	0.56315000	F	1.31197700	1.61246900	0.45703800
F	-7.47226500	-2.50434600	0.22055700	F	-0.18430800	0.65832700	-2.29599600
F	-7.99395500	-1.16351400	-1.40463200	F	0.15543600	2.68108900	-1.23481100
C	-7.18150500	-1.30537500	-0.32604600				

2.2 Planes defined using Chimera Software

**2**	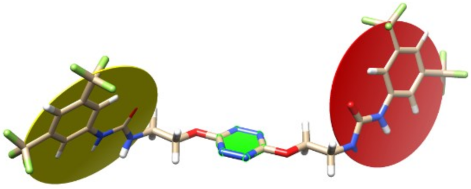	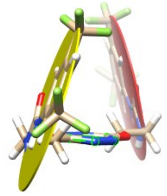
**2**-Cl	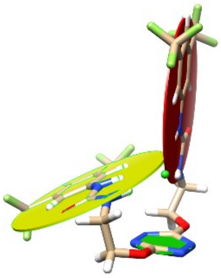	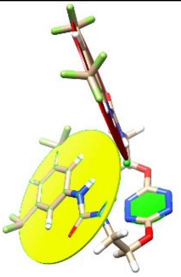
**2**-Br	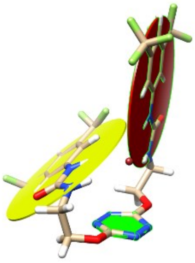	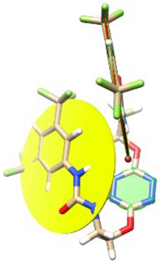
**2**-I	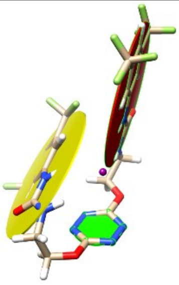	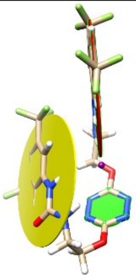
**2**-SCN	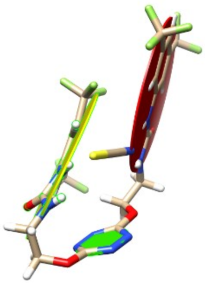	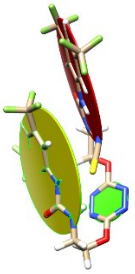
**2**-PF_6	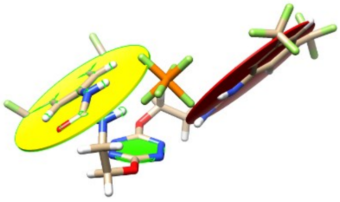	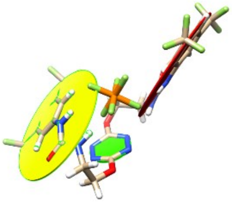

3. Synthetic procedures and Characterization data

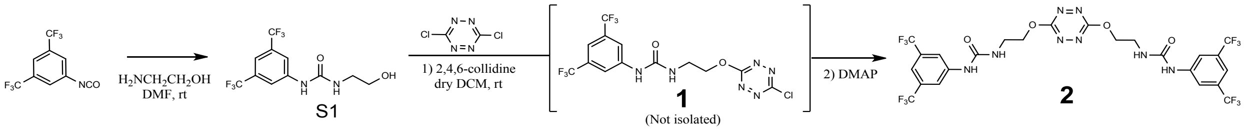

Figure S1 : General procedure for Synthesis of **2**

3.1 Preparation of 1-(3,5-bis(trifluoromethyl)phenyl)-3-(2-hydroxyethyl)urea **S1**

Procedure was reported in our previous publication. [5]

3.2 Preparation of 1,1'-(((1,2,4,5-tetrazine-3,6-diyl)bis(oxy))bis(ethane-2,1-diyl))bis(3-(3,5-bis(trifluoromethyl)phenyl)urea) **2**

Inspired from a published procedure reported by us. [6]

1-(3,5-bis(trifluoromethyl)phenyl)-3-(2-hydroxyethyl)urea (200mg, 0.63mmol, 2.0eq) was added to a round bottom flask equipped with a magnetic stirrer and dry dichloromethane (7 mL) were added under inert atmosphere. A solution of 2,4,6-collidine (87µL, 0.66mmol, 2.05eq) diluted into dry dichloromethane (1 mL) was added dropwise to the flask. Dichlorotetrazine diluted into dry dichloromethane (1.4 mL) was added to the flask dropwise. The mixture was stirred for two hours at room temperature. After checking that intermediate was formed on TLC, 4-dimethylaminopyridine (160 mg, 1.31 mmol, 4.1eq) was added to the flask and the mixture was stirred for two extra hours. The solvent was evaporated. The crude product was purified by a flash chromatography on silica gel using a solvent gradient (petroleum ether/ethyl acetate 1/1 to 4/6) followed by a washing of obtained solid with pentane. 88 mg of 1,1'-(((1,2,4,5-tetrazine-3,6-diyl)bis(oxy))bis(ethane-2,1-diyl))bis(3-(3,5-bis(trifluoromethyl)phenyl)urea) **2** were obtained as a pink fluorescent solid. Yield 88 mg, 38%

^{1}H NMR (300 MHz, Acetone d6, 25°C, TMS) δ 8.76 (s, 2H, 2 Ar-N-*H*), 8.14 (s, 4H, Ar-*H*), 7.53 (s, 2H, Ar-*H*), 6.51 (s, 2H, 2 CH$_2$-N-*H*), 4.67 (t, 4H, 2 C*H*$_2$-O), 3.77 (q, 4H, 2 C*H*$_2$-N).

^{13}C NMR (75 MHz, Acetone d6, 25°C, TMS) δ 167.2 (s, 2C, O-C_{Tz}), 155.8 (s, 2C, *C*=O), 143.4 (s, 2C, C_{arom}-N), 133.0-131.7 (q, 4C, $^2J_{C-F}$ = 33 Hz), 129.9-122.7 (q, 4C, $^3J_{C-F}$ = 270 Hz, C_{arom}), 118.5 (s, 1C, C_{arom}), 115.0 (quint, 4*C*, $^1J_{C-F}$ = 4 Hz, *C*F$_3$), 69.4 (s, 1C, *C*H$_2$-O), 39.6 (s, 1C, *C*H$_2$-N).

^{19}F NMR (282 MHz, Acetone d6, 25°C) (not calibrated) δ 113.9 (s,12F)

IR (cm^{-1}) 3347, 3086, 1648, 1577, 1472, 1428, 1269

UV/Visible (Acetonitrile) λ_{max} (ε)= 237 nm (4.00 AU), 290 nm (0.84 AU), 343 nm (0.57 AU), 520 nm (0.11 AU).

Fluorescence (Acetonitrile) λ_{exc}= 520 nm, λ_{em}= 572 nm.

HRMS (ESI$^+$-TOF) *m/z [M+Na]$^+$* calculated for $C_{24}H_{18}F_{12}N_8NaO_4$: 733.1158, found 733.1157

Rf 0.32 in petroleum ether/ethyl acetate (4:6)

Melting Point 206-210°C

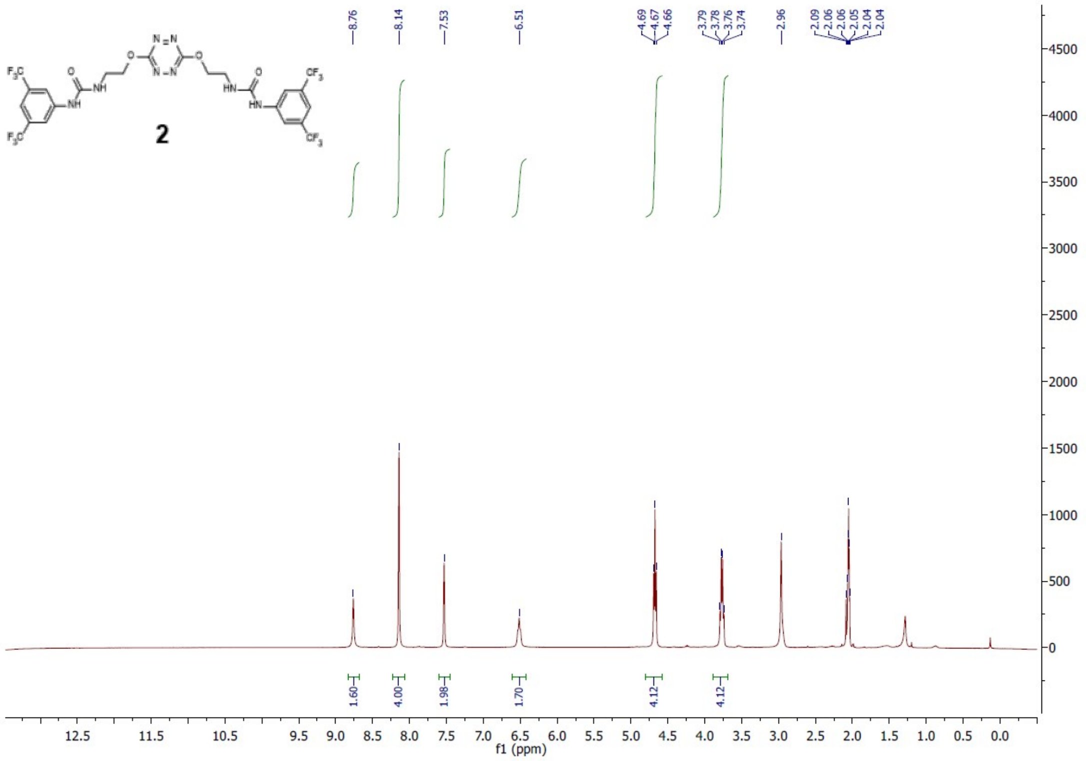

*Figure S2:¹H NMR (300 MHz) spectrum of **2** in acetone d6*

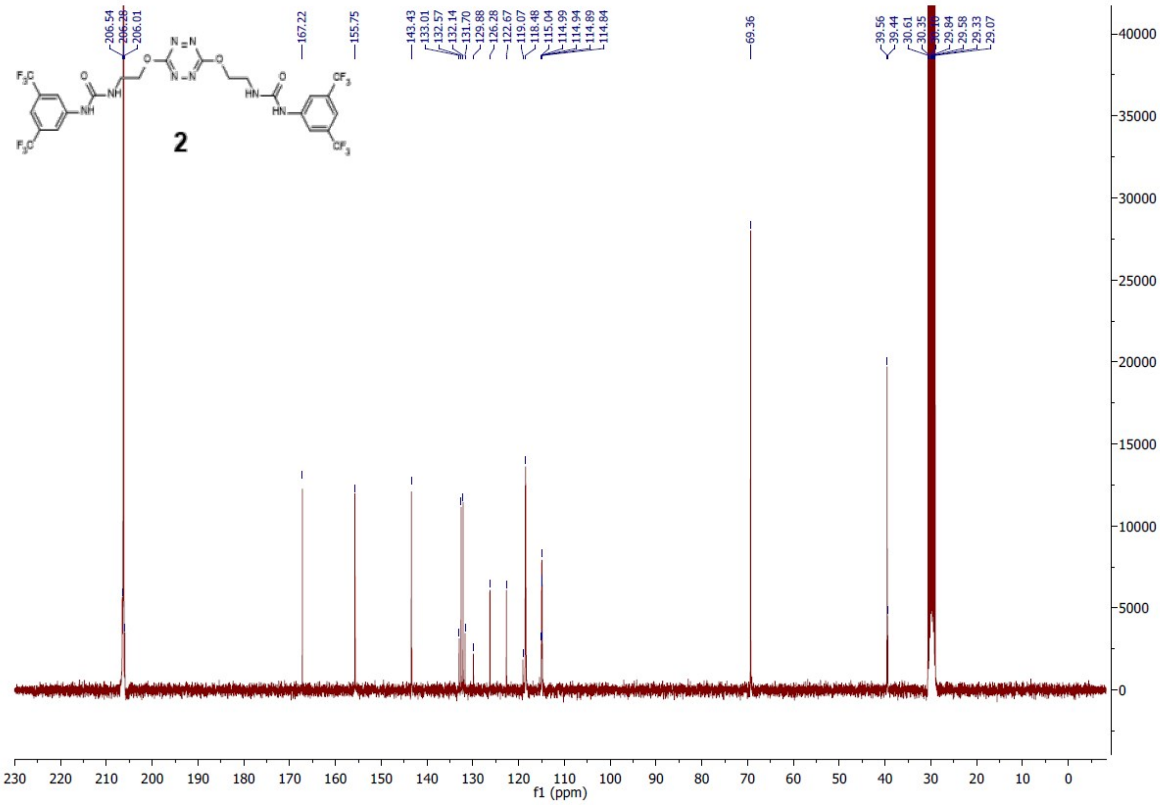

*Figure S3: ¹³C NMR (75 MHz) spectrum of **2** in acetone d6*

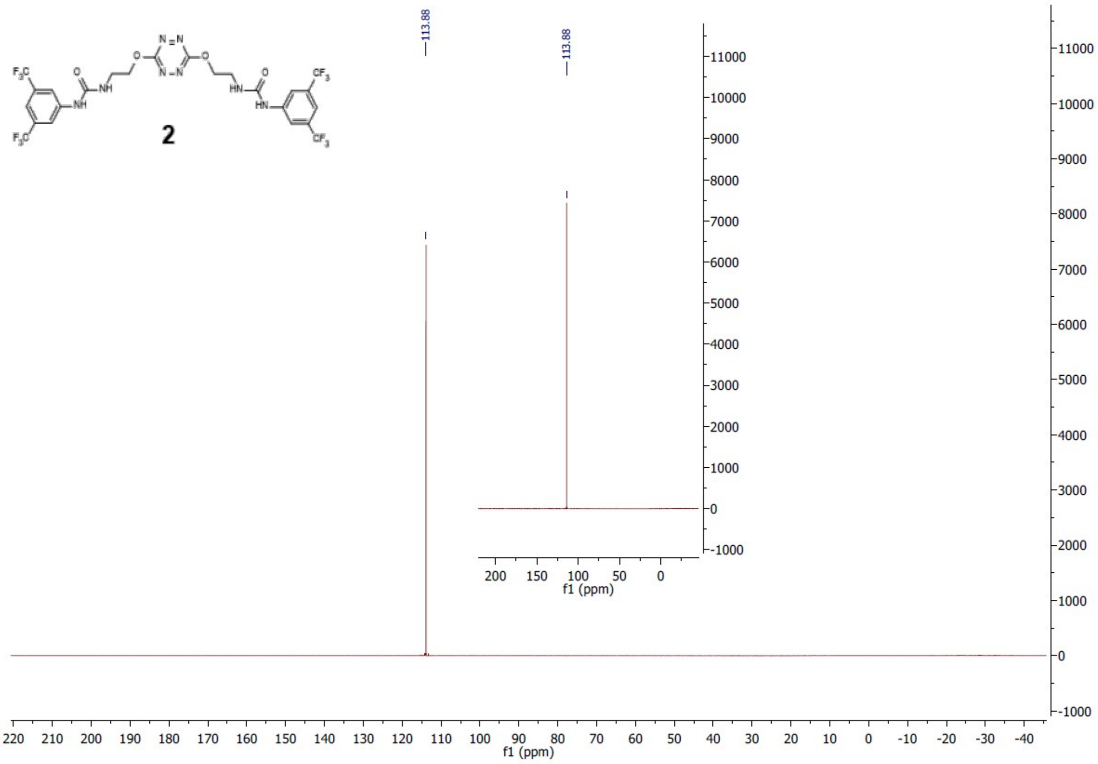

*Figure S4: ^{19}F NMR (282 MHz) spectrum of **2** in acetone d6*

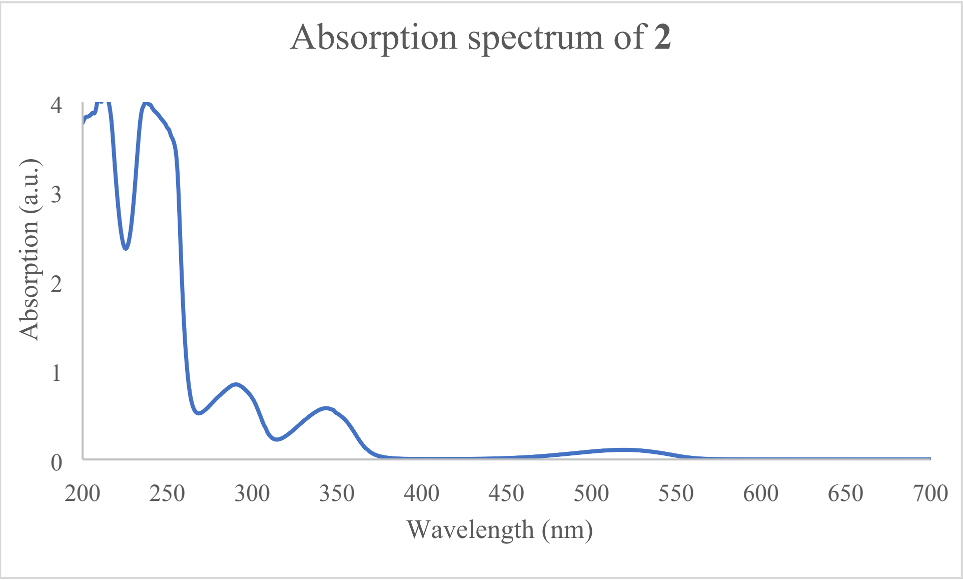

*Figure S5: Absorption spectrum of **2** in acetonitrile*

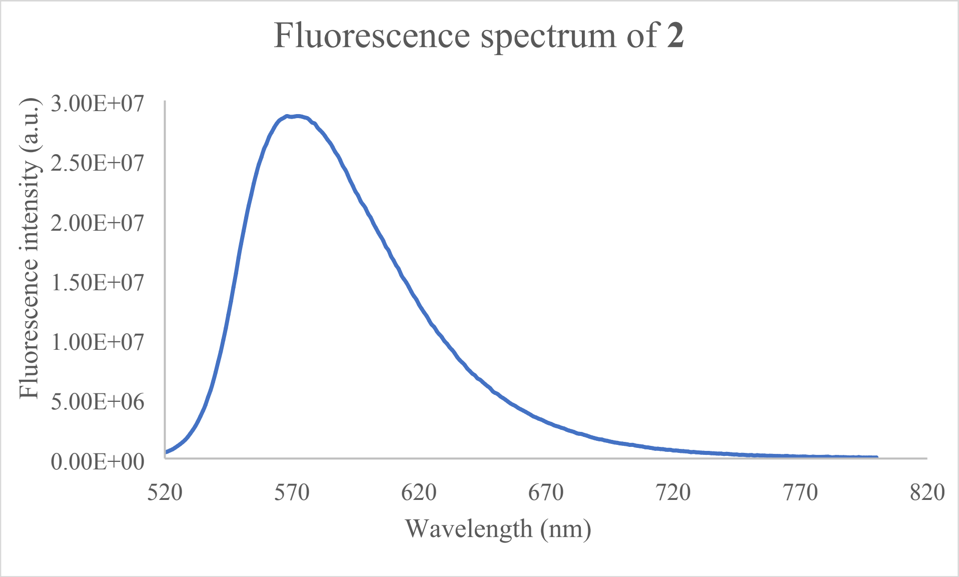

*Figure S6: Fluorescence spectrum of **2** in acetonitrile*

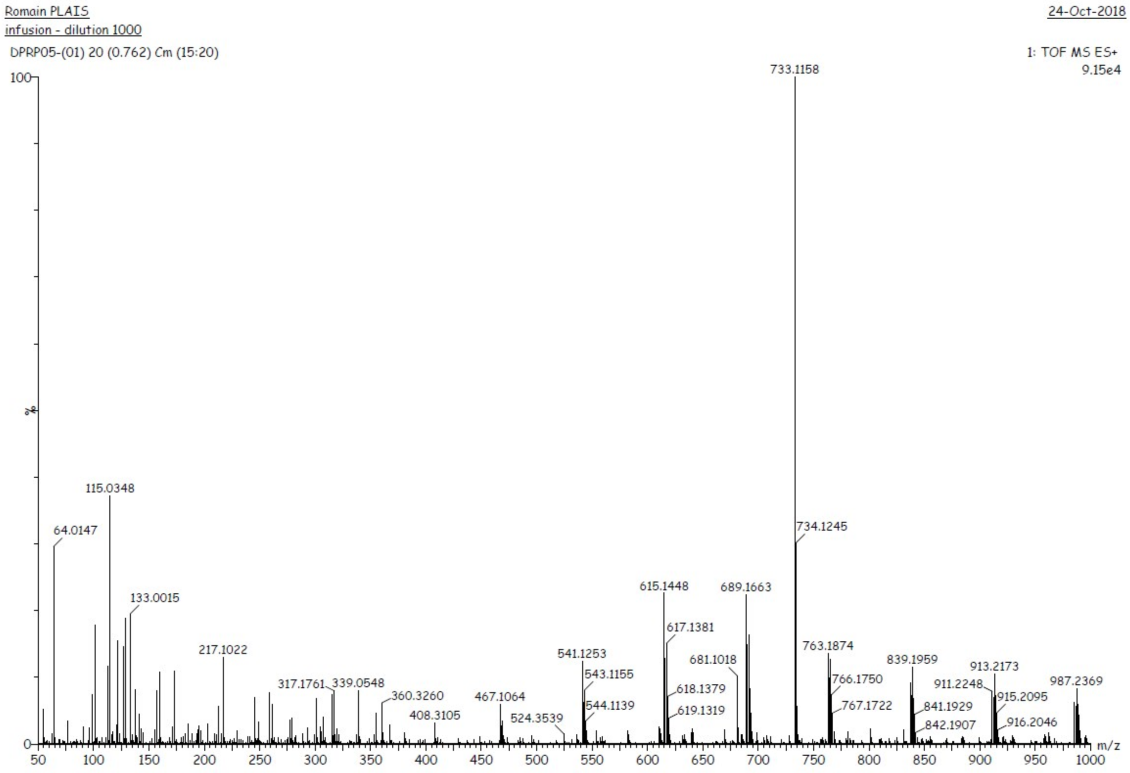

*Figure S7: Mass spectrum of **2** (TOF ES+). Note also the presence of PDMS contaminant*

Elemental Composition Report **Page 1**

Single Mass Analysis
Tolerance = 5.0 PPM / DBE: min = -1.5, max = 100.0
Element prediction: Off
Number of isotope peaks used for i-FIT = 7

Monoisotopic Mass, Even Electron Ions
9730 formula(e) evaluated with 57 results within limits (all results (up to 1000) for each mass)
Elements Used:
C: 0-150 H: 0-150 N: 0-10 O: 0-10 F: 6-12 Na: 1-1 I: 0-2

Romain PLAIS 24-Oct-2018
Infusion - dilution 1000
DPRP05-(01) 20 (0.762) Cm (15:20) 1: TOF MS ES+ 9.15e+004

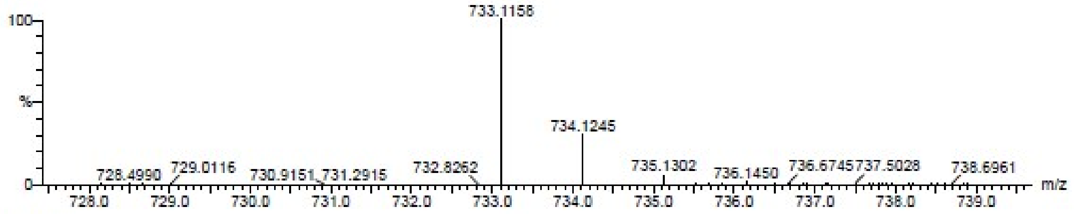

Mass	Calc. Mass	mDa	PPM	DBE	i-FIT	i-FIT (Norm)	Formula
Minimum:				-1.5			
Maximum:		5.0	5.0	100.0			
733.1158	733.1158	0.0	0.0	8.5	511.6	6.3	C22 H29 N8 O4 F6 Na I
	733.1157	0.1	0.1	13.5	509.8	4.4	C24 H18 N8 O4 F12 Na
	733.1159	-0.1	-0.1	24.5	514.7	9.4	C32 H17 N8 O4 F7 Na
	733.1157	0.1	0.1	15.5	509.9	4.6	C28 H22 N4 O9 F8 Na
	733.1156	0.2	0.3	-0.5	515.2	9.9	C18 H34 N4 O9 F7 Na I
	733.1161	-0.3	-0.4	4.5	511.2	5.8	C24 H32 N2 O4 F9 Na I
	733.1161	-0.3	-0.4	20.5	514.5	9.1	C34 H20 N2 O4 F10 Na
	733.1153	0.5	0.7	8.5	513.8	8.4	C18 H20 N10 O9 F10 Na
	733.1164	-0.6	-0.8	4.5	516.3	11.0	C15 H21 N10 O10 F11 Na
	733.1166	-0.8	-1.1	15.5	506.8	1.5	C23 H20 N10 O10 F6 Na
	733.1150	0.8	1.1	24.5	516.4	11.1	C37 H19 N2 O3 F9 Na
	733.1149	0.9	1.2	8.5	509.5	4.2	C27 H31 N2 O3 F8 Na I
	733.1168	-1.0	-1.4	11.5	506.5	1.1	C25 H23 N4 O10 F9 Na
	733.1147	1.1	1.5	28.5	516.7	11.3	C35 H16 N8 O3 F6 Na
	733.1170	-1.2	-1.6	4.5	514.4	9.1	C19 H30 N8 O5 F7 Na I
	733.1146	1.2	1.6	17.5	510.4	5.0	C27 H17 N8 O3 F11 Na
	733.1170	-1.2	-1.6	20.5	512.1	6.8	C29 H18 N8 O5 F8 Na
	733.1145	1.3	1.8	1.5	516.8	11.5	C17 H29 N8 O3 F10 Na I
	733.1145	1.3	1.8	3.5	512.4	7.0	C21 H33 N4 O8 F6 Na I
	733.1145	1.3	1.8	19.5	513.3	8.0	C31 H21 N4 O8 F7 Na
	733.1144	1.4	1.9	8.5	509.7	4.4	C23 H22 N4 O8 F12 Na
	733.1172	-1.4	-1.9	0.5	514.0	8.7	C21 H33 N2 O5 F10 Na I
	733.1173	-1.5	-2.0	16.5	511.8	6.5	C31 H21 N2 O5 F11 Na
	733.1174	-1.6	-2.2	27.5	517.8	12.4	C39 H20 N2 O5 F6 Na
	733.1174	-1.6	-2.2	9.5	511.8	6.4	C25 H28 N6 F9 Na I

*Figure S8: Single mass analysis of **2** (TOF ES+)*

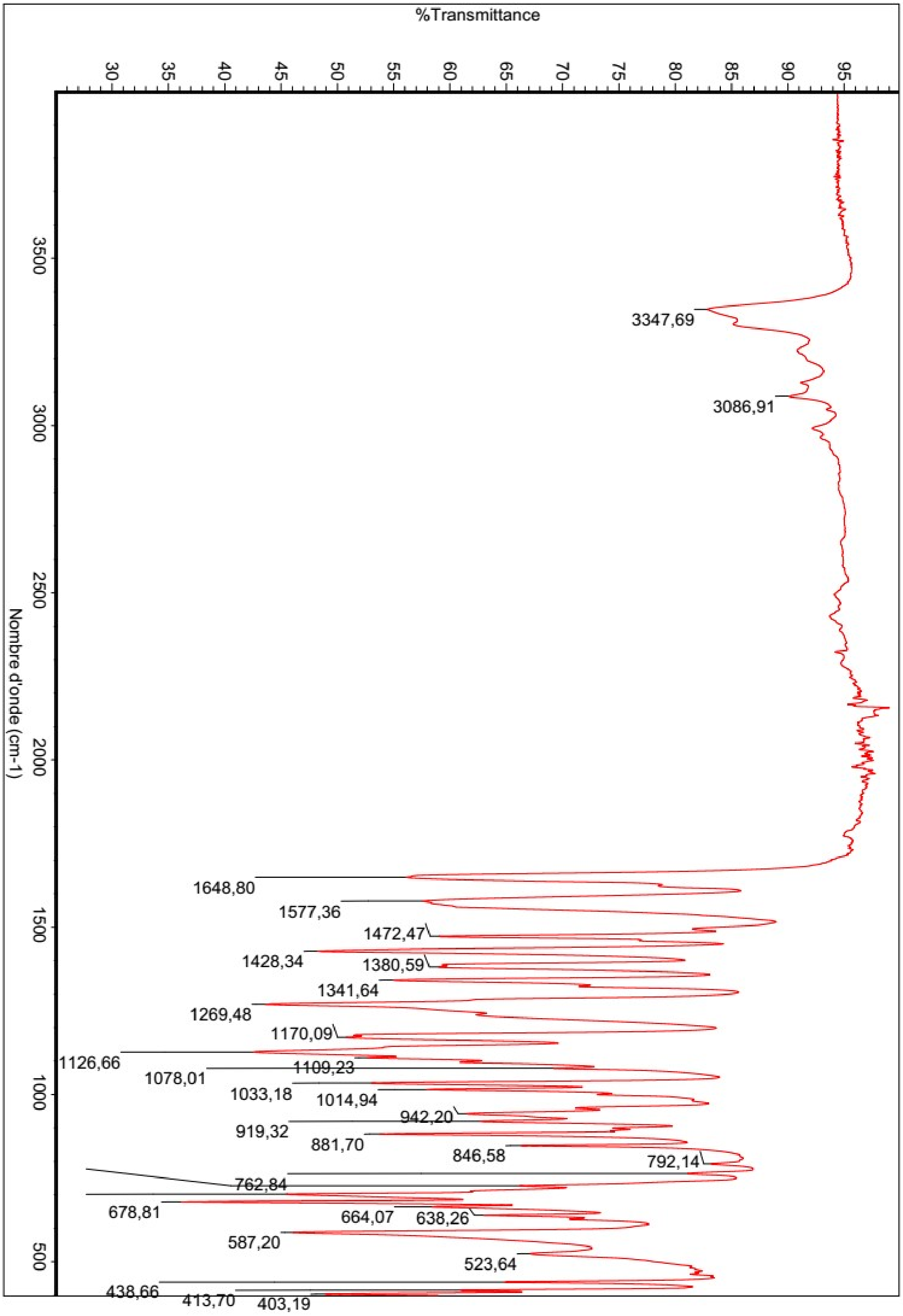

*Figure S9: Infrared spectrum of **2***

4. Mass spectrometry experiments

4.1 Fragmentation mechanisms associated to *Scheme 3*

m/z 490, 513, 534, 582, for X=Cl⁻, SCN⁻, Br⁻, I⁻, respectively.

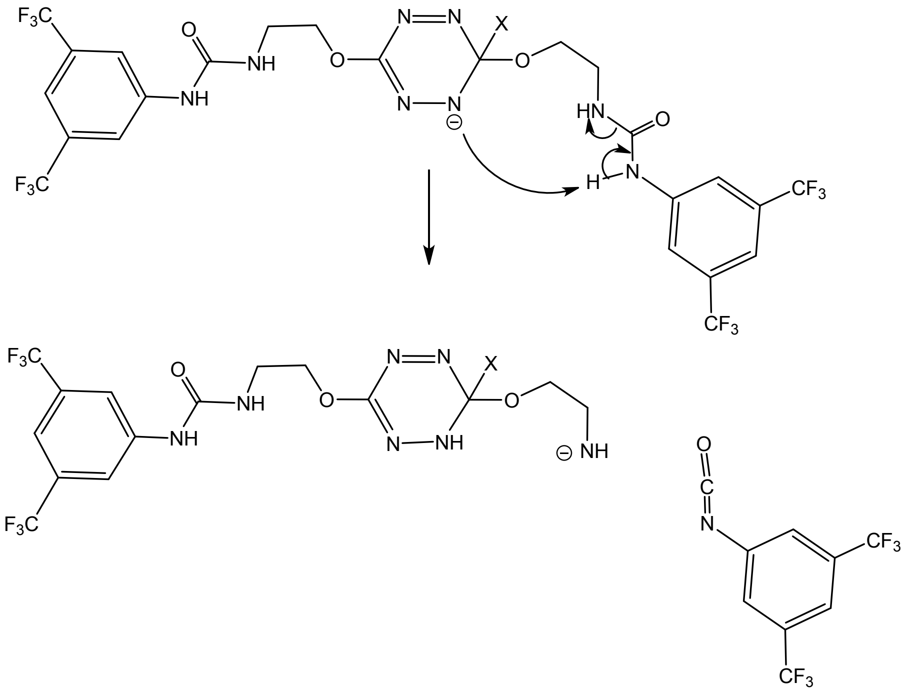

Scheme S1 Formation of [$C_{15}H_{15}F_6XN_7O_3$]$^-$

m/z 491, 539, for Br⁻, I⁻, respectively.

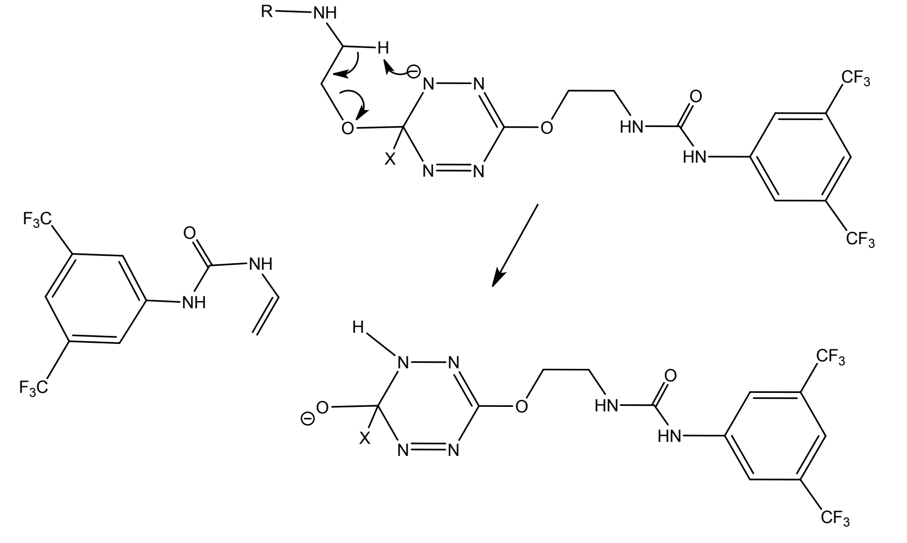

Scheme S2 Formation of [$C_{13}H_{10}N_6O_3F_6X$]$^-$

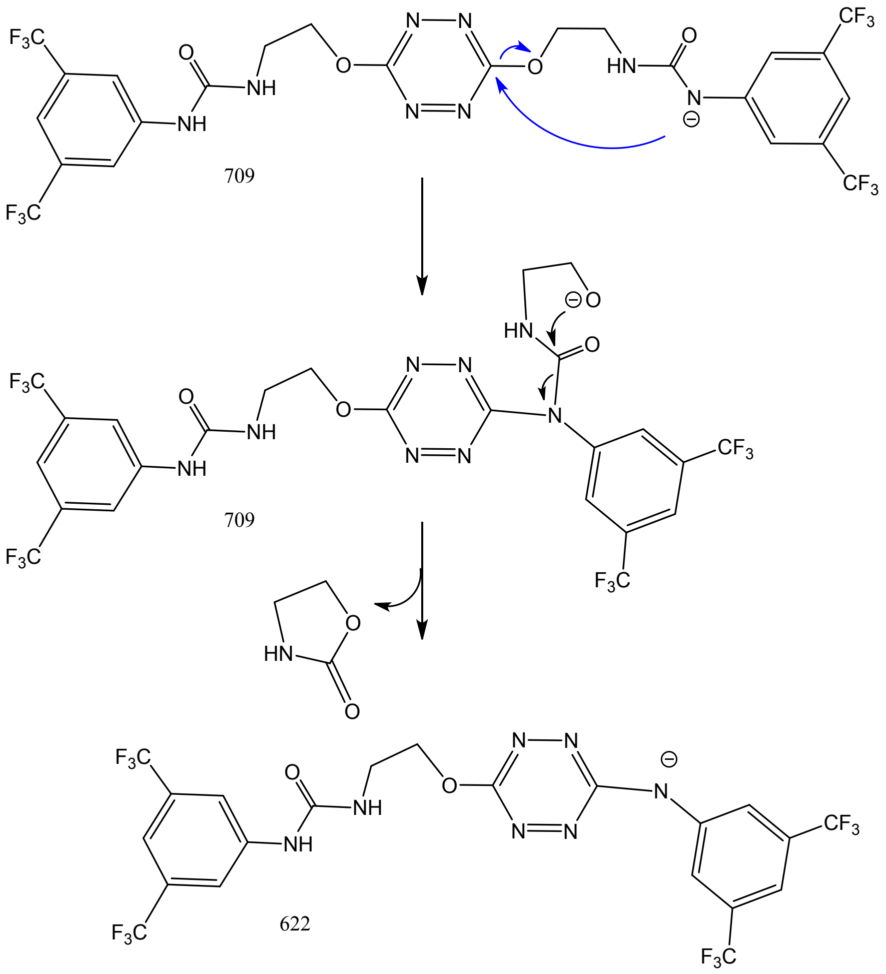

*Scheme S3 Formation of m/z 622 from [(**2**)-H]⁻*

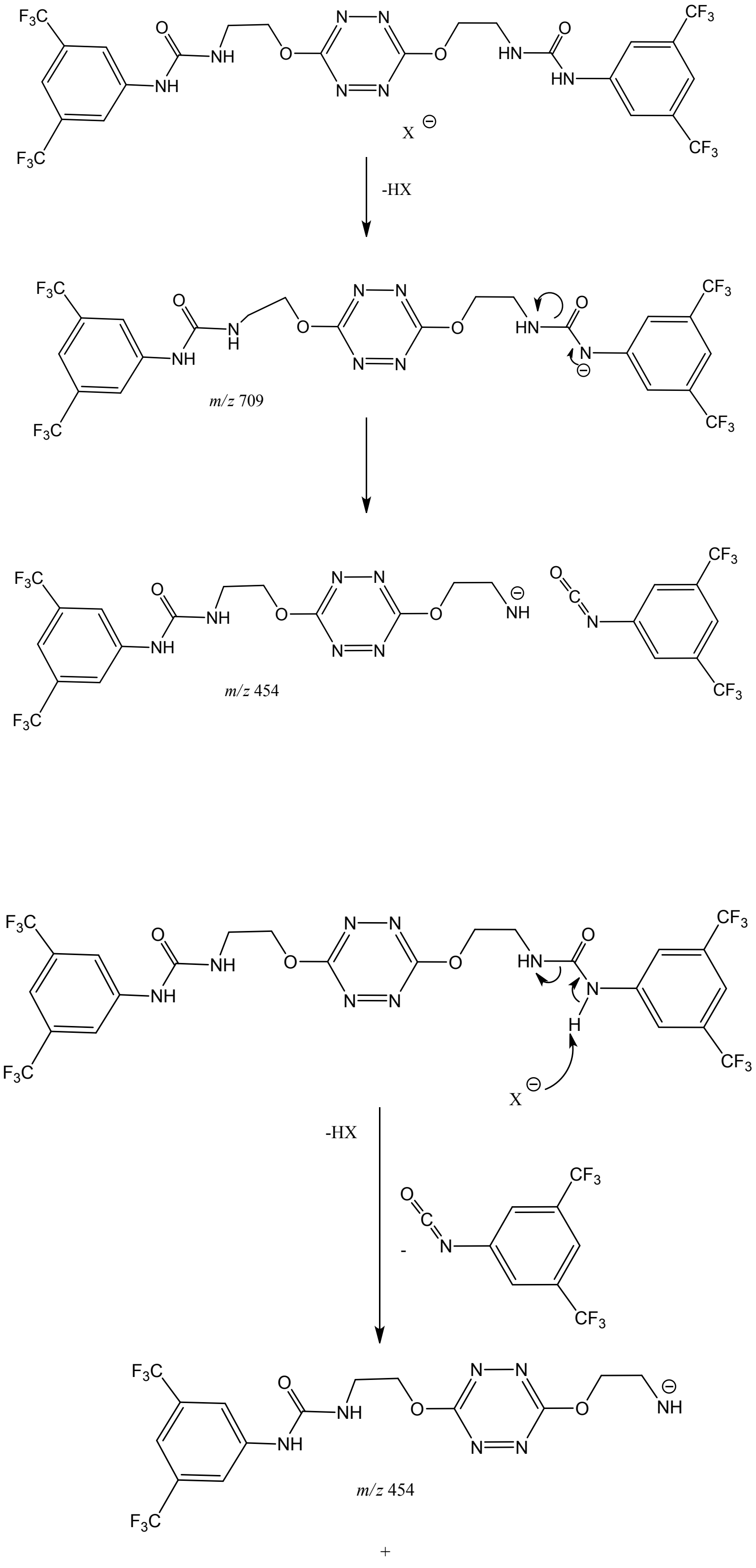

*Scheme S4 Formation of m/z 454 from [(**2**)-X]⁻ and [(**2**)-H]⁻*

4.2 Typical MS/MS spectra obtained for the different adducts

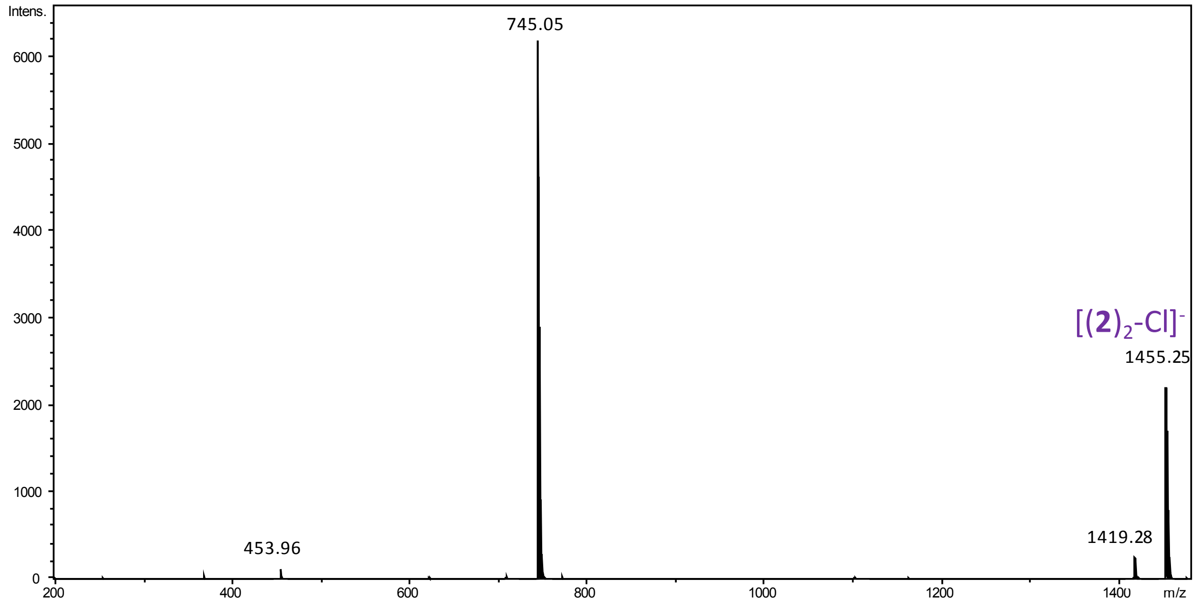

*Figure S10: MS/MS spectrum of the [(**2**)$_2$-Cl]$^-$ adduct*

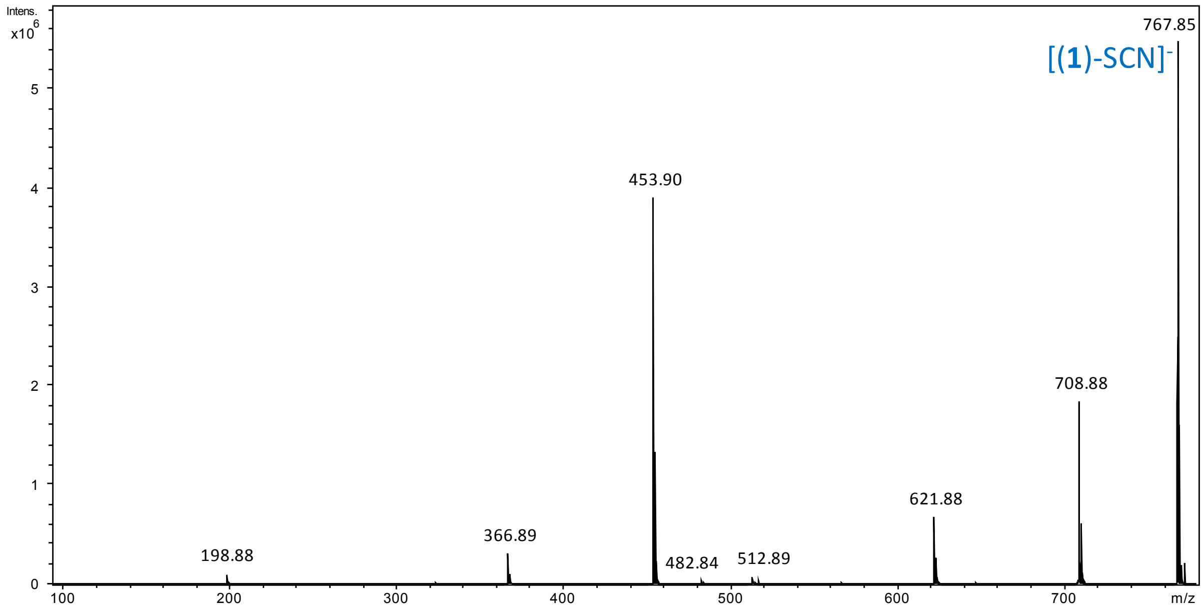

*Figure S11: MS/MS spectrum of the [(**2**)-SCN]$^-$ adduct*

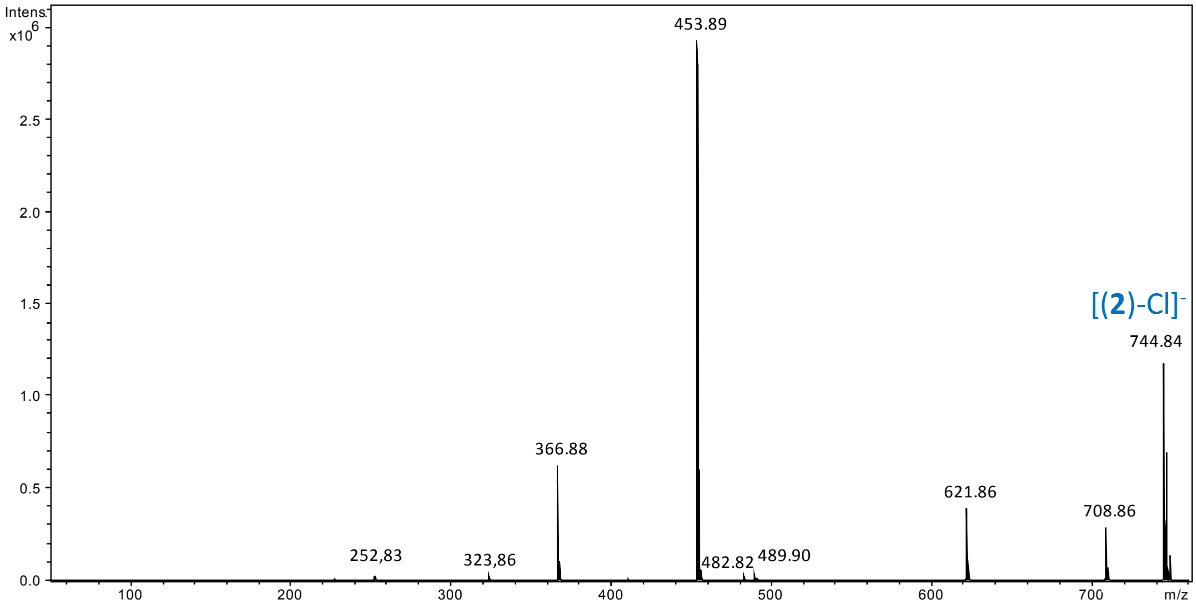

*Figure S12: MS/MS spectrum of the [(**2**)-Cl]⁻ adduct*

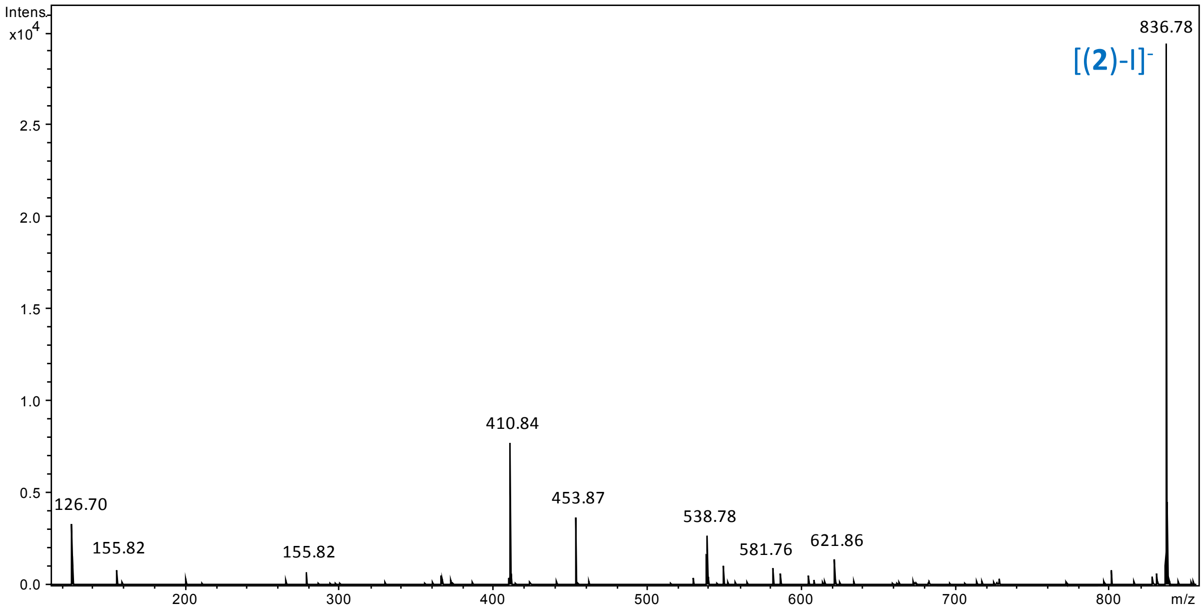

*Figure S13: MS/MS spectrum of the [(**2**)-I]⁻ adduct*

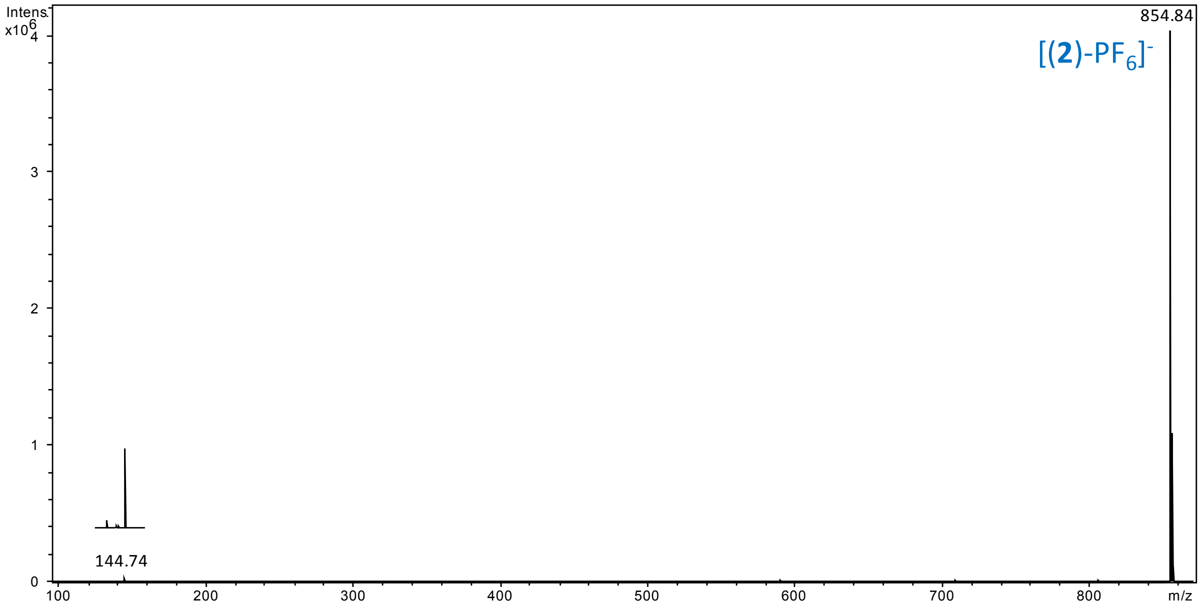

*Figure S14: MS/MS spectrum of the [(**2**)-PF_6]$^-$ adduct*

5. NMR Titrations

5.1 Practical analysis procedure

Previously to each analysis, anion salts were solubilized into acetone and precipitated by addition of diethylether to remove water. Salts were then dried to remove residual solvents and stored in the dessicator until use.

2mL of a solution containing the anion receptor was prepared (3.5mmol/L). 500µL were placed into a new NMR tube. 1mL of stock solution was taken and desired amount of anionic guest as the tetrabutylammonium (NBu_4^+) salt was added.

NMR titrations were performed by adding aliquots of a solution containing the anionic guest (0.07M for NBu_4Cl, 0.28M for NBu_4Br, 0.81M for NBu_4I and 1.83M for NBu_4SCN) and the receptor (3.5mM) in MeCN-d_3 to the NMR tube. After each addition, a ^1H NMR spectrum was recorded.

^1H NMR spectra were calibrated to the residual proton solvent peak in MeCN-d_3 (δ = 1.94ppm) at 300 K. Plot stackings were made using MestReNova Version 6.0. Non-linear least-square curve fitting of the titration data were double checked to be a 1:1 binding model using a reported procedure [7] on Excel software and SPECFIT software.

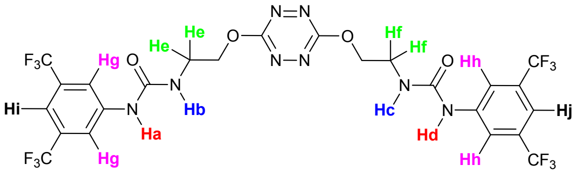

Figure S15: Proton attribution

5.2 Titration of **2** with NBu_4Cl

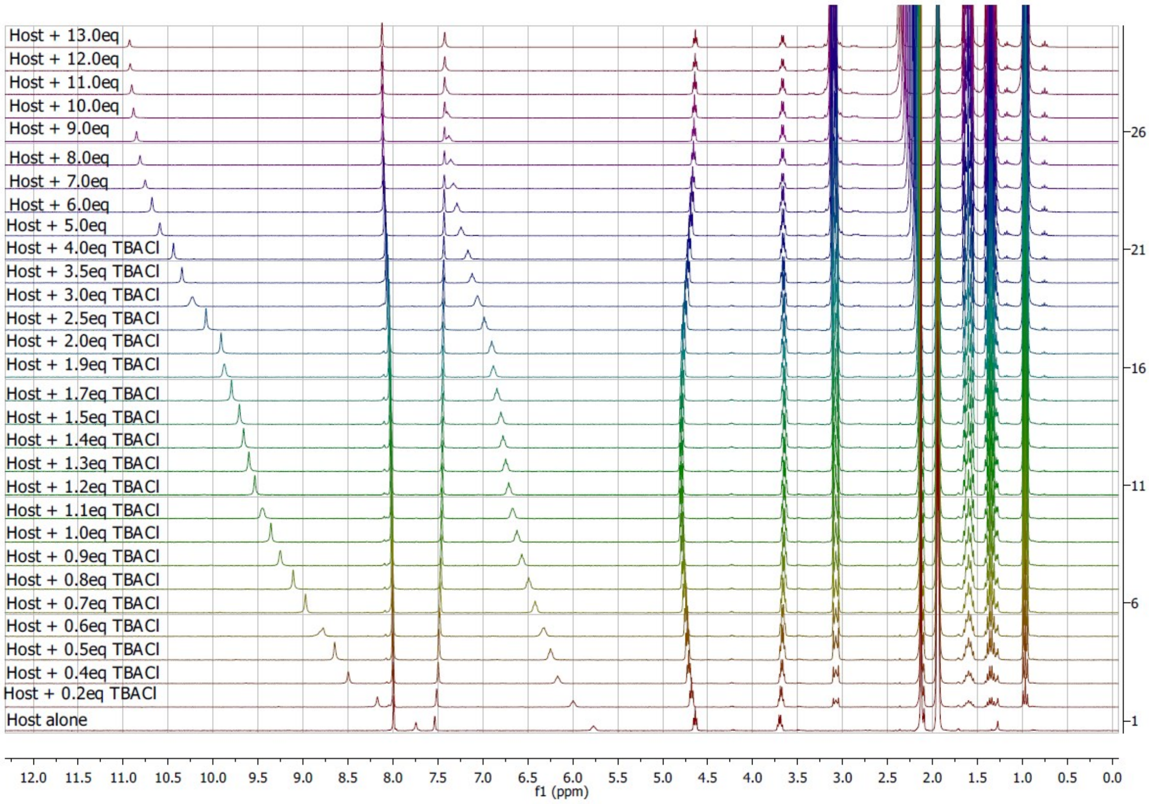

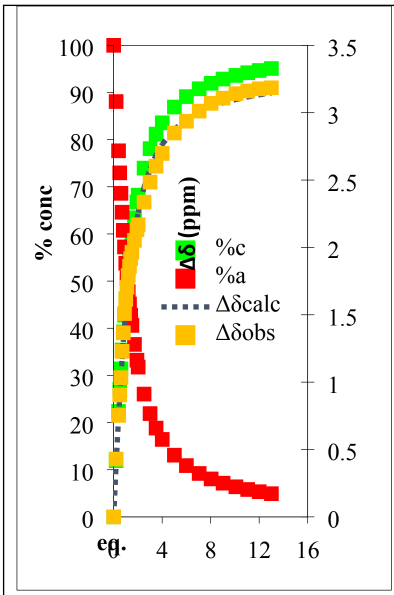	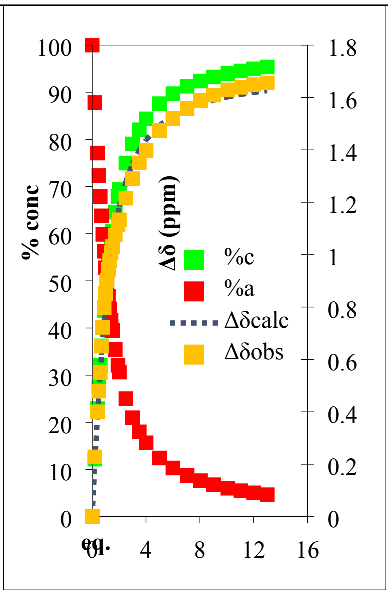	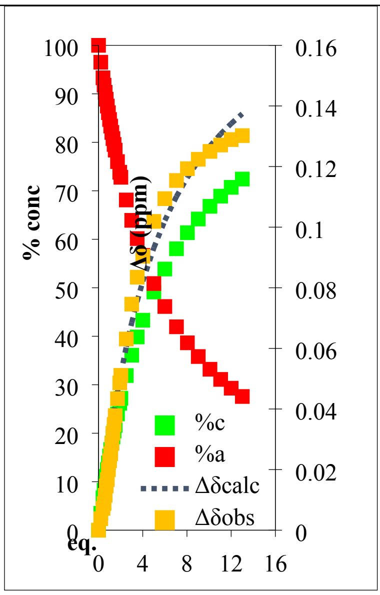
**Ha/Hd**	**Hb/Hc**	**He/Hf**
Ka = 459 L/mol	Ka = 487 L/mol	Ka = 61 L/mol
[ligand] = 0.0035 M	[ligand] = 0.0035 M	[ligand] = 0.0035 M
$\delta_{c,\ lim}$ = 11.05 ppm	$\delta_{c,\ lim}$ = 7.47 ppm	$\delta_{c,\ lim}$ = 8.18 ppm

*Figure S16: ^{1}H NMR titration of **2** with tetrabutylammonium chloride (0 to 13 equivalents)*
%c = percentage of complex %a = percentage of free receptor Δδcalc = chemical shift calculated Δδobs = chemical shift observed

[PROGRAM]
Name = SPECFIT
Version = 3.0

[FILE]
Name = RP05+TBACL_RMN_FORMAT_SPECFIT.FAC
Path = C:\Program Files\SPECFIT\DATA\
Date = 18-juil-19
Time = 18:52:51
Ncomp = 2
Nmeas = 30
Nwave = 3

[FACTOR ANALYSIS]
Tolerance = 1,000E-09
Max.Factors = 10
Num.Factors = 3
Significant = 3
Eigen Noise = 5,373E-08
Exp't Noise = 5,373E-08

#	Eigenvalue	Square Sum	Residual	Prediction
1	6,265E+03	8,364E+00	3,066E-01	Data Vector
2	8,362E+00	1,750E-03	4,459E-03	Data Vector
3	1,750E-03	2,512E-13	5,373E-08	Data Vector

[MODEL]
Date = 18-juil-19
Time = 18:53:14
Model = 0
Index = 3
Function = 1
Species = 3
Params = 3

[SPECIES]	[COLORED]	[FIXED]	[SPECTRUM]
1 0 0	False	False	
0 1 0	True	False	
1 1 0	True	False	

[SPECIES]	[FIXED]	[PARAMETER]		[ERROR]
1 0 0	True	0,00000E+00	+/-	0,00000E+00
0 1 0	True	0,00000E+00	+/-	0,00000E+00
1 1 0	False	2,62757E+00	+/-	1,73555E-02

[CONVERGENCE]
Iterations = 3
Convergence Limit = 1,000E-03
Convergence Found = 4,861E-08
Marquardt Parameter = 0,0
Sum(Y-y)^2 Residuals = 1,18399E-01
Std. Deviation of Fit(Y) = 3,64737E-02

[STATISTICS]
Experimental Noise = 5,373E-08
Relative Error Of Fit = 0,4344%
Durbin-Watson Factor = 0,1878
Goodness Of Fit, Chi^2 = 4,608E+11
Durbin-Watson Factor (raw data) = None
Goodness Of Fit, Chi^2 (raw data) = None

[COVARIANCE]
1,662E-03

[CORRELATION]
1,000E+00

[END FILE]

*Figure S17: Determination of binding constant using SPECFIT software for the ^{1}H NMR titration of **2** with tetrabutylammonium chloride*

5.3 Titration of **2** with NBu_4Br

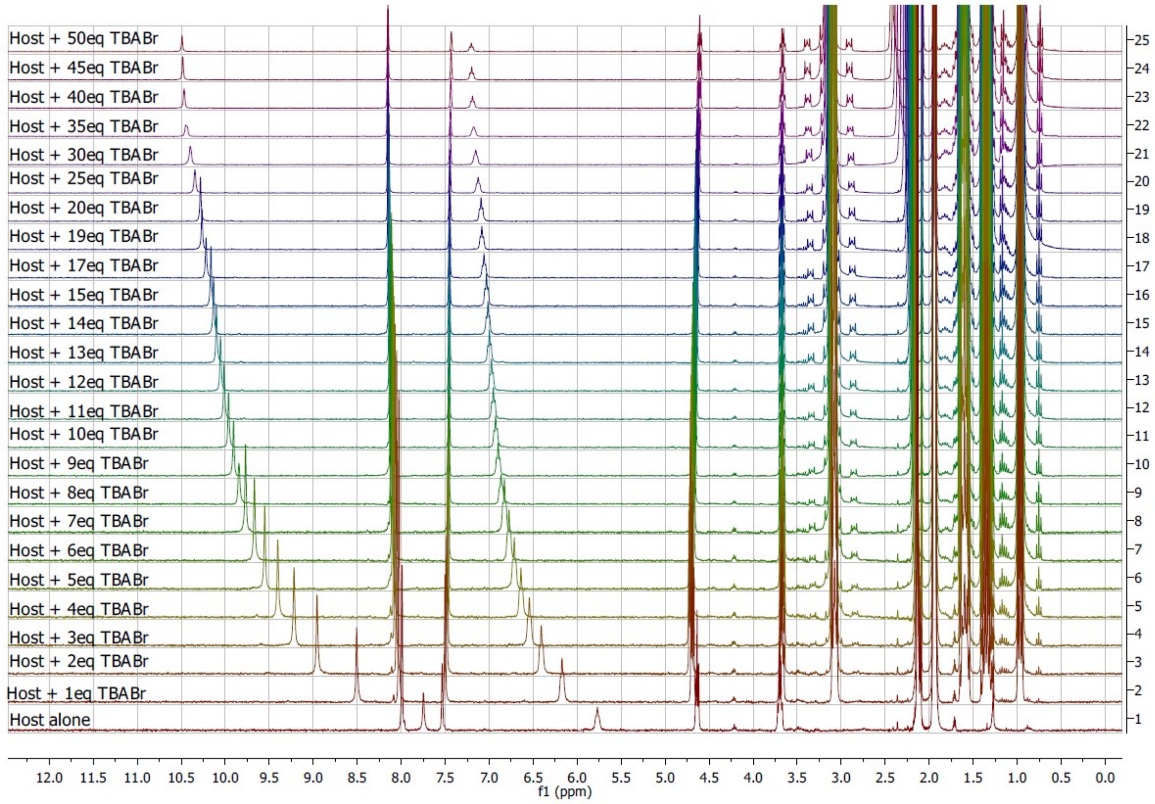

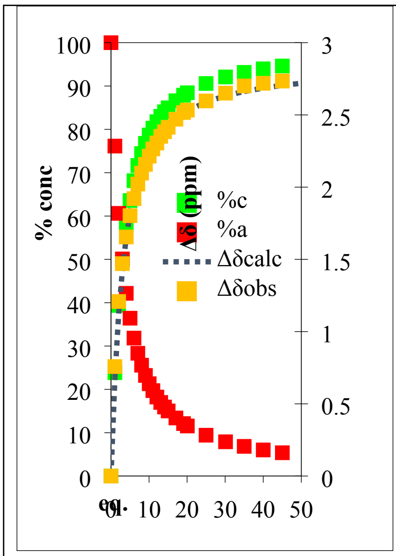

Ha/Hd

Ka = 114 L/mol

[ligand] = 0.0035 M

$\delta_{c,\ lim}$ = 10.61 ppm

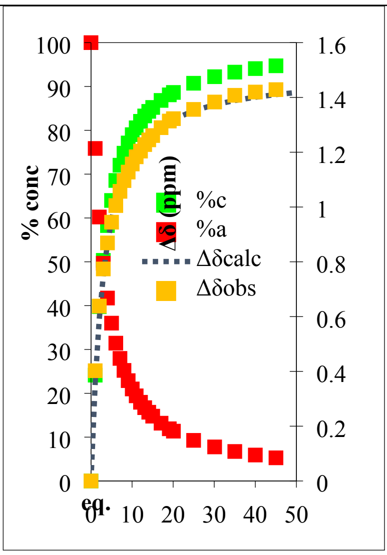

Hb/Hc

Ka = 116 L/mol

[ligand] = 0.0035 M

$\delta_{c,\ lim}$ = 7.26 ppm

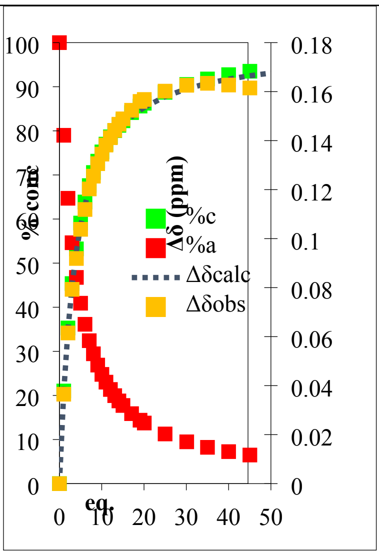

He/Hf

Ka = 93 L/mol

[ligand] = 0.0035 M

$\delta_{c,\ lim}$ = 8.17 ppm

*Figure S18: ^{1}H NMR titration of **2** with tetrabutylammonium bromide (0 to 50 equivalents)*
%c = percentage of complex %a = percentage of free receptor Δδcalc = chemical shift calculated Δδobs = chemical shift observed

[PROGRAM]
Name = SPECFIT
Version = 3.0

[FILE]
Name = RP05+TBABR_RMN_FORMAT_SPECFIT.FAC
Path = C:\Program Files\SPECFIT\DATA\
Date = 07-juil-20
Time = 11:12:15
Ncomp = 2
Nmeas = 25
Nwave = 3

[FACTOR ANALYSIS]
Tolerance = 1,000E-09
Max.Factors = 10
Num.Factors = 3
Significant = 3
Eigen Noise = 9,923E-08
Exp't Noise = 9,923E-08

#	Eigenvalue	Square Sum	Residual	Prediction
1	5,265E+03	3,714E+00	2,240E-01	Data Vector
2	3,714E+00	1,409E-04	1,389E-03	Data Vector
3	1,409E-04	7,090E-13	9,923E-08	Data Vector

[MODEL]
Date = 07-juil-20
Time = 11:12:46
Model = 0
Index = 3
Function = 1
Species = 3
Params = 3

[SPECIES]	[COLORED]	[FIXED]	[SPECTRUM]
1 0 0	False	False	
0 1 0	True	False	
1 1 0	True	False	

[SPECIES]	[FIXED]	[PARAMETER]		[ERROR]
1 0 0	True	0,00000E+00	+/-	0,00000E+00
0 1 0	True	0,00000E+00	+/-	0,00000E+00
1 1 0	False	2,47058E+00	+/-	5,89609E-02

[CONVERGENCE]
Iterations = 21
Convergence Limit = 1,000E-03
Convergence Found = -8,040E-01
Marquardt Parameter = 1,000E-07
Sum(Y-y)^2 Residuals = 8,72600E-01
Std. Deviation of Fit(Y) = 1,08590E-01

[STATISTICS]
Experimental Noise = 9,923E-08
Relative Error Of Fit = 1,2870%
Durbin-Watson Factor = 0,2703
Goodness Of Fit, Chi^2 = 1,198E+12
Durbin-Watson Factor (raw data) = None
Goodness Of Fit, Chi^2 (raw data) = None

[COVARIANCE]
2,114E-02

[CORRELATION]
1,000E+00

[END FILE]

*Figure S19: Determination of binding constant using SPECFIT software for the ^{1}H NMR titration of **2** with tetrabutylammonium bromide*

5.4 Titration of **2** with NBu_4I

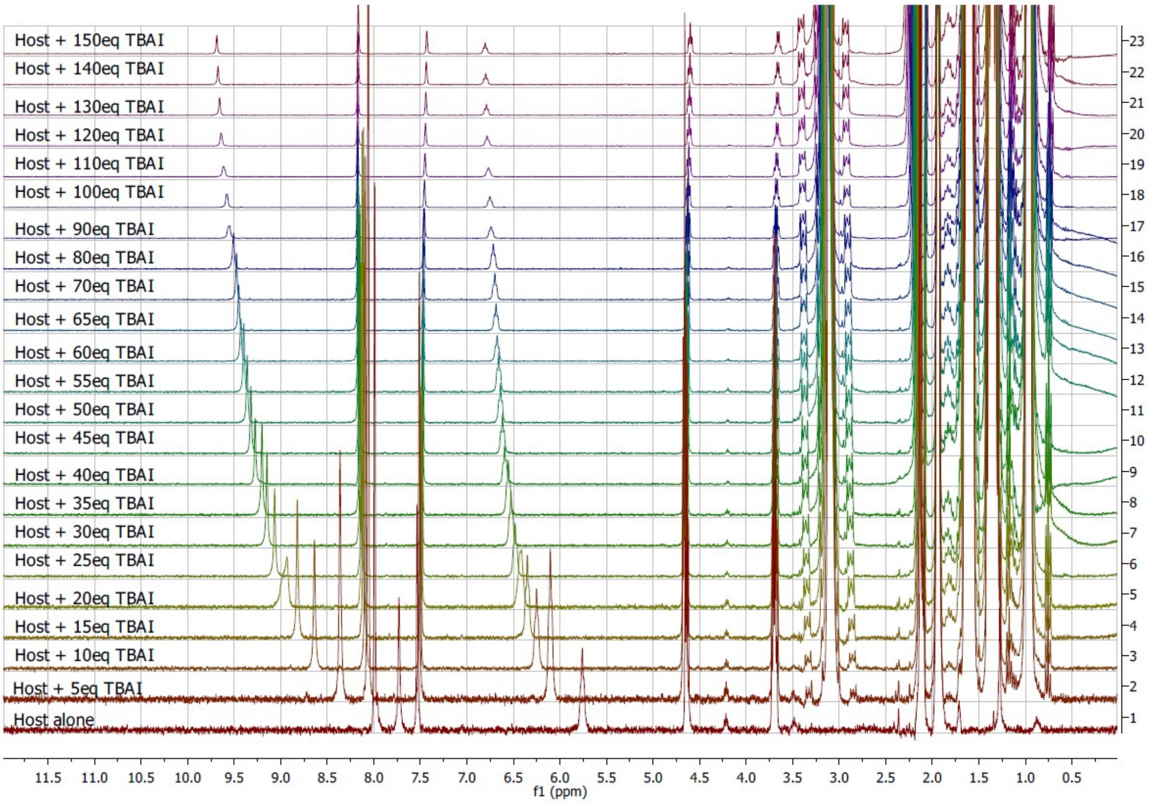

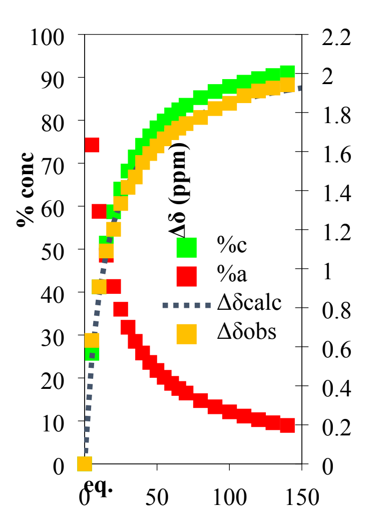

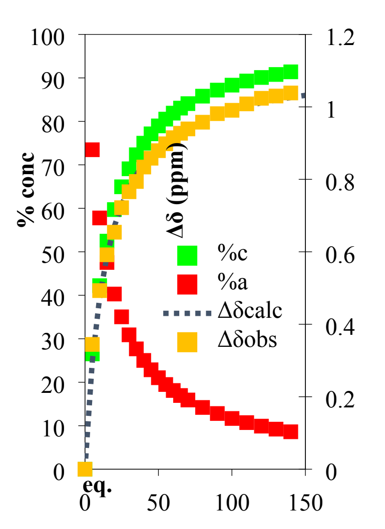

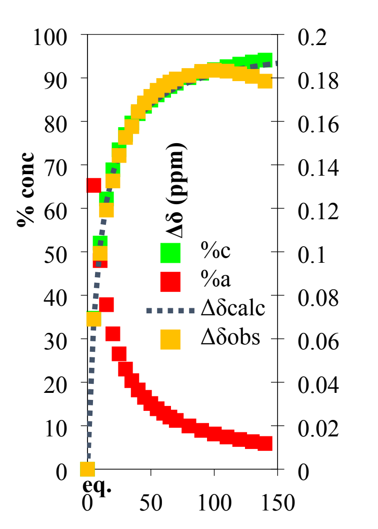

**Ha/Hd**	**Hb/Hc**	**He/Hf**
Ka = 21 L/mol	Ka = 22 L/mol	Ka = 33 L/mol
[ligand] = 0.0035 M	[ligand] = 0.0035 M	[ligand] = 0.0035 M
$\delta_{c,\ lim}$ = 9.83 ppm	$\delta_{c,\ lim}$ = 7.26 ppm	$\delta_{c,\ lim}$ = 8.19 ppm

*Figure S20: ^{1}H NMR titration of **2** with tetrabutylammonium iodide (0 to 150 equivalents)*
%c = percentage of complex %a = percentage of free receptor Δδcalc = chemical shift calculated Δδobs = chemical shift observed

[PROGRAM]
Name = SPECFIT
Version = 3.0

[FILE]
Name = RP05+TBAI_RMN_FORMAT_SPECFIT.FAC
Path = C:\Program Files\SPECFIT\DATA\
Date = 07-juil-20
Time = 11:27:37
Ncomp = 2
Nmeas = 23
Nwave = 3

[FACTOR ANALYSIS]
Tolerance = 1,000E-09
Max.Factors = 10
Num.Factors = 3
Significant = 3
Eigen Noise = 9,983E-08
Exp't Noise = 9,983E-08

#	Eigenvalue	Square Sum	Residual	Prediction
1	4,488E+03	1,808E+00	1,631E-01	Data Vector
2	1,808E+00	7,420E-05	1,052E-03	Data Vector
3	7,420E-05	6,577E-13	9,983E-08	Data Vector

[MODEL]
Date = 07-juil-20
Time = 11:27:53
Model = 0
Index = 3
Function = 1
Species = 3
Params = 3

[SPECIES]	[COLORED]	[FIXED]	[SPECTRUM]
1 0 0	False	False	
0 1 0	True	False	
1 1 0	True	False	

[SPECIES]	[FIXED]	[PARAMETER]		[ERROR]
1 0 0	True	0,00000E+00	+/-	0,00000E+00
0 1 0	True	0,00000E+00	+/-	0,00000E+00
1 1 0	False	1,30176E+00	+/-	1,32427E-02

[CONVERGENCE]
Iterations = 15
Convergence Limit = 1,000E-03
Convergence Found = 3,520E-05
Marquardt Parameter = 0,0
Sum(Y-y)^2 Residuals = 2,00560E-02
Std. Deviation of Fit(Y) = 1,71739E-02

[STATISTICS]
Experimental Noise = 9,983E-08
Relative Error Of Fit = 0,2114%
Durbin-Watson Factor = 1,2721
Goodness Of Fit, Chi^2 = 2,960E+10
Durbin-Watson Factor (raw data) = None
Goodness Of Fit, Chi^2 (raw data) = None

[COVARIANCE]
9,587E-04

[CORRELATION]
1,000E+00

[END FILE]

*Figure S21: Determination of binding constant using SPECFIT software for the ^{1}H NMR titration of **2** with tetrabutylammonium iodide*

5.5 Titration of **2** with NBu_4SCN

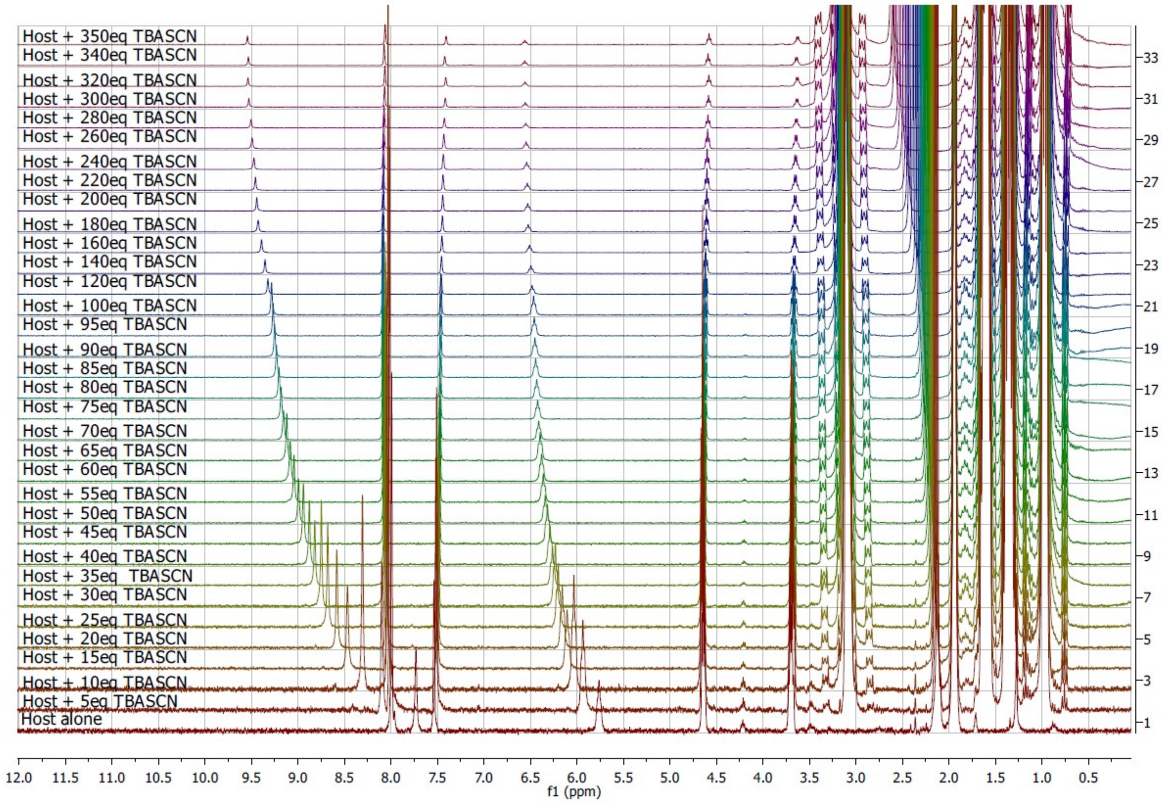

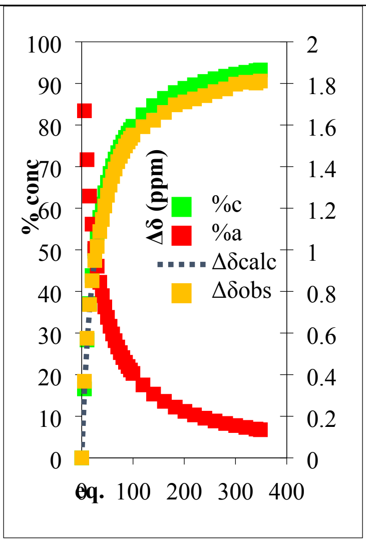	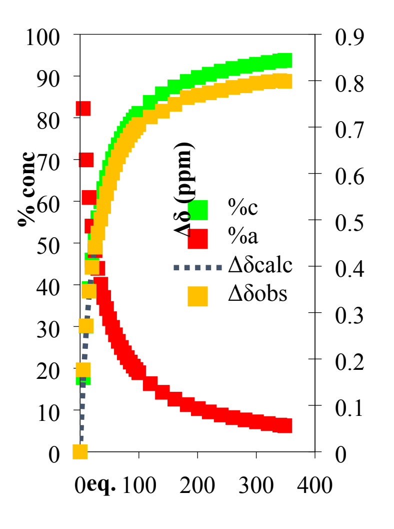	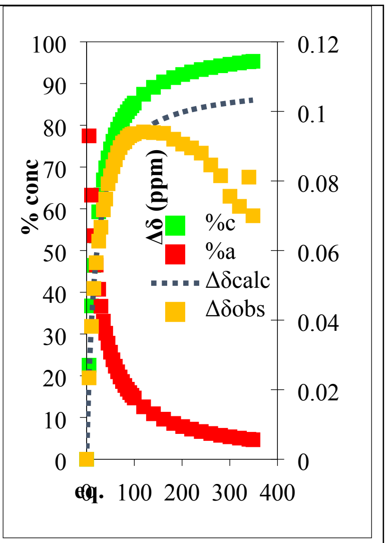
**Ha/Hd**	**Hb/Hc**	**He/Hf**
Ka = 11 L/mol	Ka = 12 L/mol	Ka = 17 L/mol
[ligand] = 0.0035 M	[ligand] = 0.0035 M	[ligand] = 0.0035 M
$\delta_{c,\ lim}$ = 9.66 ppm	$\delta_{c,\ lim}$ = 6.62 ppm	$\delta_{c,\ lim}$ = 8.10 ppm

*Figure S22: ^{1}H NMR titration of **2** with tetrabutylammonium thiocyanate (0 to 320 equivalents)*
%c = percentage of complex %a = percentage of free receptor Δδcalc = chemical shift calculated Δδobs = chemical shift observed

[PROGRAM]
Name = SPECFIT
Version = 3.0

[FILE]
Name = RP05+TBASCN_RMN_FORMAT_SPECFIT.FAC
Path = C:\Program Files\SPECFIT\DATA\
Date = 07-juil-20
Time = 11:33:16
Ncomp = 2
Nmeas = 34
Nwave = 3

[FACTOR ANALYSIS]
Tolerance = 1,000E-09
Max.Factors = 10
Num.Factors = 3
Significant = 3
Eigen Noise = 7,562E-08
Exp't Noise = 7,562E-08

#	Eigenvalue	Square Sum	Residual	Prediction
1	6,410E+03	2,615E+00	1,609E-01	Data Vector
2	2,614E+00	4,718E-04	2,172E-03	Data Vector
3	4,718E-04	5,661E-13	7,562E-08	Data Vector

[MODEL]
Date = 07-juil-20
Time = 11:33:38
Model = 0
Index = 3
Function = 1
Species = 3
Params = 3

[SPECIES]	[COLORED]	[FIXED]	[SPECTRUM]
1 0 0	False	False	
0 1 0	True	False	
1 1 0	True	False	

[SPECIES]	[FIXED]	[PARAMETER]		[ERROR]
1 0 0	True	0,00000E+00	+/-	0,00000E+00
0 1 0	True	0,00000E+00	+/-	0,00000E+00
1 1 0	False	1,04307E+00	+/-	8,05608E-03

[CONVERGENCE]
Iterations = 8
Convergence Limit = 1,000E-03
Convergence Found = 6,397E-07
Marquardt Parameter = 0,0
Sum(Y-y)^2 Residuals = 1,39250E-02
Std. Deviation of Fit(Y) = 1,17419E-02

[STATISTICS]
Experimental Noise = 7,562E-08
Relative Error Of Fit = 0,1474%
Durbin-Watson Factor = 0,5562
Goodness Of Fit, Chi^2 = 2,411E+10
Durbin-Watson Factor (raw data) = None
Goodness Of Fit, Chi^2 (raw data) = None

[COVARIANCE]
3,505E-04

[CORRELATION]
1,000E+00

[END FILE]

*Figure S23: Determination of binding constant using SPECFIT software for the ^{1}H NMR titration of **2** with tetrabutylammonium thiocyanate*

5.6 Superposition of experimental curves

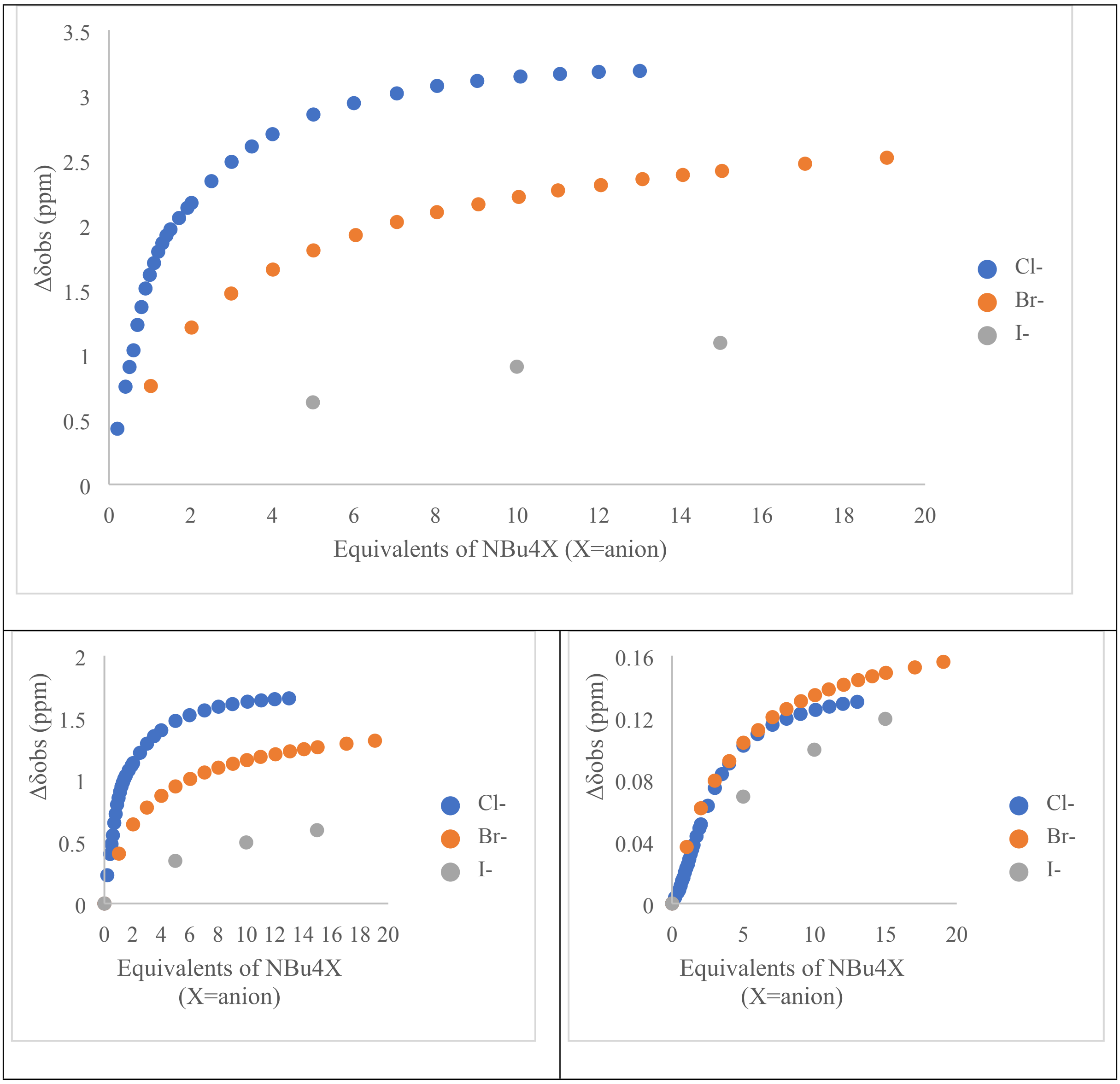

Figure S24: Differences in chemical shifts observed as a function of the number of equivalents of salts added protons a, b and c

6. Photophysical analysis and procedures

6.1 General practical analysis procedure

Previously to each analysis, anion salts were solubilized into acetone and precipitated by addition of diethylether to remove water. Salts were then dried to remove residual solvents and stored in the dessicator until use.

10mL of a stock solution of the anion receptor were prepared (10^{-3}mol/L) in acetonitrile. 2mL of this solution was taken and diluted at 2.10^{-5}mol/L. 2mL of this solution at 2.10^{-5}mol/L were introduced into a quartz cuvette. 2mL of the stock solution was taken, desired amount of salt was added (55mg for Bu_4NI, 330mg for Bu_4NBr, 600mg for Bu_4NI and 600mg for Bu_4NSCN) and diluted at 2.10^{-5}mol/L.

2.5mL of solution were added to a 1cm quartz glass cuvette. Aliquots of the solution containing the anion and the receptor are subsequently added to the sample cuvette for each measurement.

After blank subtraction, absorbance spectra were measured from 200 to 700nm. From the absorbance spectra were determined the absorbance maximum (520nm), that corresponds to the excitation wavelength for the emission spectra.

Emission spectra were measured from 530 nm ($\lambda_{abs,max}$+10) to 700nm using the wavelength determined before as excitation wavelength. Slites were calibrated at 2.1nm. All experiments were proceeded in temperature-controlled room at 300K.

Fluorescence decay data were analyzed using the Globals software package developed at the Laboratory for Fluorescence Dynamics at the University of Illinois at Urbana-Champaign, which includes reconvolution analysis and global non-linear least-squares minimization method.

Experimental measurements were plotted using Excel software. Determination of binding constants was done using a method developed by Valeur *et al.* [8] using non-linear least-squares minimization method.

$$Y = Y_0 + \frac{Y_{\text{lim}} - Y_0}{2}\left\{1 + \frac{c_M}{c_L} + \frac{1}{K_s c_L} - \left[\left(1 + \frac{c_M}{c_L} + \frac{1}{K_s c_L}\right)^2 - 4\frac{c_M}{c_L}\right]^{1/2}\right\}$$

Where :

Y : Measured intensity at fluorescence maximum

Y_0 : Measured intensity when no salt was added

Y_{lim} : Calculated intensity when an infinity of equivalents of salts are added

c_M : Anion concentration

c_L : Receptor concentration

K_s : Association constant of receptor/anion complex

Binding constants were also determined using SPECFIT/32 ™ Global Analysis System software [9,10]. This software allows global analysis of equilibrium and kinetic systems with Expanded SVD and nonlinear regression modeling by the Levenberg-Marquardt method.

6.2 Determination of quantum yield of **2**

Emission spectra of reference and compound **2** were recorded using the maximum absorption wavelength of the reference, Rhodamine-6G, as excitation wavelength. The fluorescence quantum yield Φ_F was determined using Rhodamine-6G as reference (Φ_F= 0.91 in ethanol). [11]

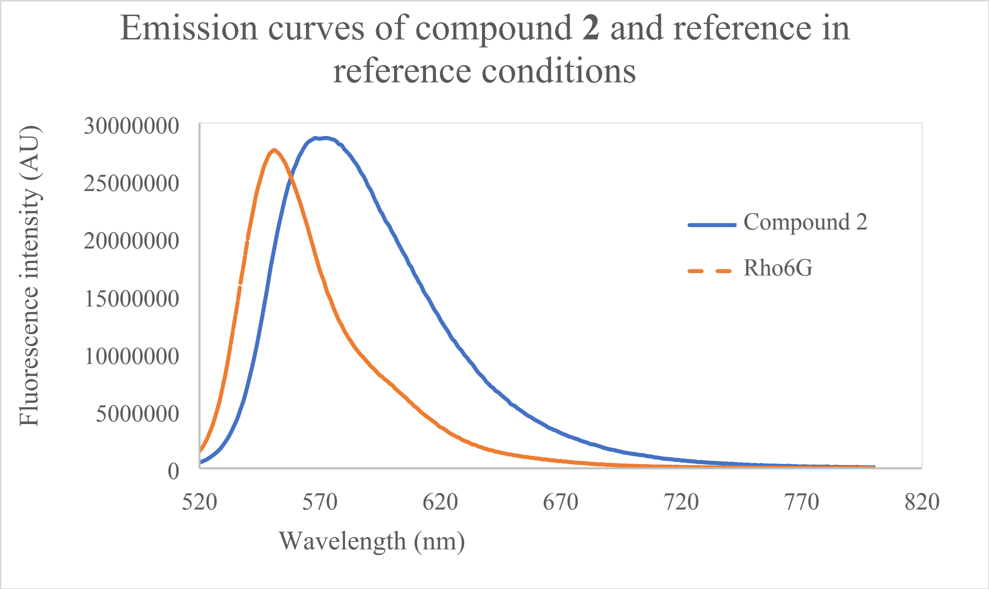

*Figure S25: Emission curves of compound **2** and reference in reference conditions*

From these spectra was determined the quantum yield using the definition described by Brouwer *et al.* [12] *:*

$$\Phi_f^i = \frac{F^i f_s n_i^2}{F^s f_i n_s^2} \Phi_f^s$$

Where :

Φ_f^i and Φ_f^s are fluorescence quantum yields of sample and standard reference

F^i and F^s are integrated areas of sample and standard fluorescence curves

f_i and f_s are absorption factors of sample and standard reference, calculated from the formula $f_x = 1 - 10^{-A_x}$, where A_x is the absorbance of species x

n_i and n_s correspond to refraction indexes of sample and standard reference

6.3 Time Dependent DFT analysis of compound **2**

In order to attribute the bands of the different fragments of **2**, theoretical UV spectra were calculated on three compounds **2**

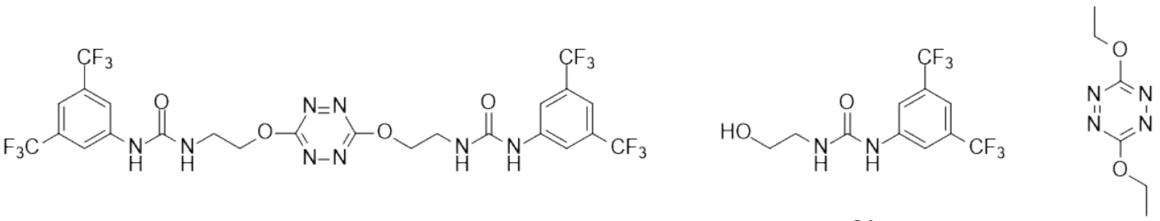

2 **S1** **Tz**

Figure S26: Compounds studied by time dependent DFT analysis

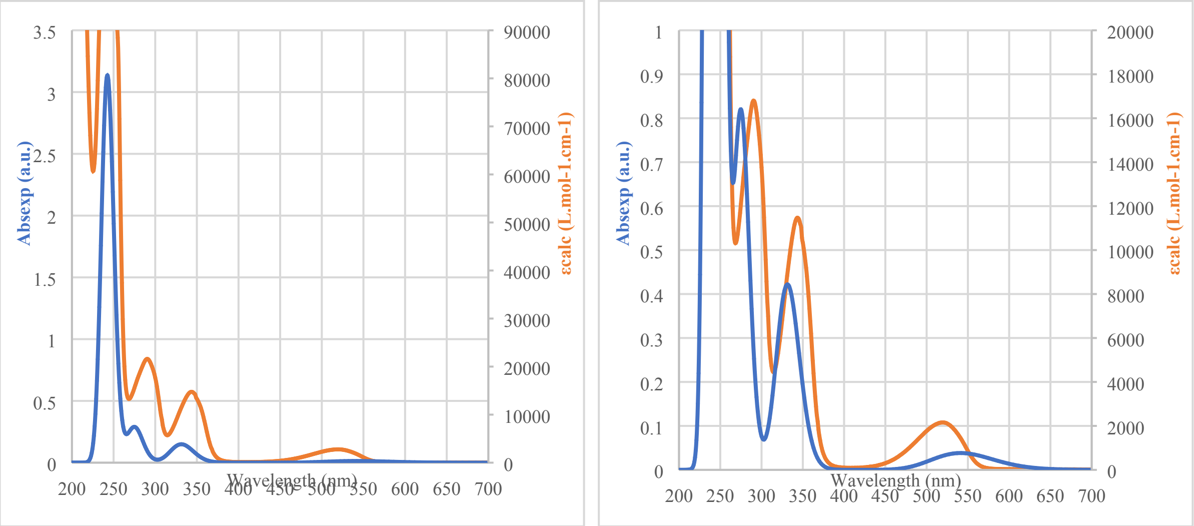

Figure S27: Experimental (orange) and calculated (blue) absorption spectra

No.	*Wavelength (nm)*	*Osc. Strength*	*Major contributions*	*Transition*
1	***539***	***0,0048***	***H-2->LUMO (100%)***	***n-π* Tz***
2	*367*	*0*	*HOMO->LUMO (98%)*	*CT Phényl->Tz*
3	*366*	*0*	*H-1->LUMO (98%)*	*CT Phényl->Tz*
4	***329***	***0,0521***	***H-3->LUMO (98%)***	***π-π* Tz***
5	***295***	***0,0019***	***H-4->LUMO (96%)***	***n(urée)-π*(Tz)***
6	*293*	*0*	*H-5->LUMO (97%)*	*n(urée)-π*(Tz)*
7	***273***	***0,0516***	***H-1->L+1 (72%), HOMO->L+1 (19%)***	***π-π* Phényl***
8	***272***	***0,0518***	***HOMO->L+2 (72%), H-1->L+2 (19%)***	***π-π* Phényl***
9	*269*	*0*	*H-2->L+5 (99%)*	
10	*266*	*0*	*H-9->LUMO (17%), H-8->LUMO (76%)*	
11	*266*	*0,0003*	*H-9->LUMO (63%), H-8->LUMO (23%), H-6->LUMO (13%)*	
12	*258*	*0*	*H-7->LUMO (93%)*	
13	*258*	*0*	*H-9->LUMO (18%), H-6->LUMO (75%)*	
14	*253*	*0*	*H-13->LUMO (76%), H-11->LUMO (18%)*	
15	***241***	***0,8059***	***HOMO->L+3 (57%), H-1->L+4 (19%), H-1->L+3 (14%)***	***π-π* Phényl***

16	***240***	***0,3037***	***H-1->L+4 (55%), HOMO->L+3 (18%), HOMO->L+4 (15%)***	***π-π* Phényl***
17	*235*	*0*	*H-1->L+1 (21%), HOMO->L+1 (79%)*	
18	*235*	*0*	*H-1->L+2 (79%), HOMO->L+2 (21%)*	
19	*232*	*0,0001*	*H-12->LUMO (25%), H-10->LUMO (73%)*	
20	*229*	*0,0004*	*H-2->L+1 (99%)*	
21	*229*	*0,0002*	*H-2->L+2 (99%)*	
22	*222*	*0*	*H-15->LUMO (58%), HOMO->L+5 (33%)*	
23	*222*	*0*	*H-15->LUMO (31%), HOMO->L+5 (65%)*	
24	*221*	*0*	*H-1->L+5 (97%)*	

Figure S28: Calculated transitions (major transitions in bold)

6.4 Titration of **2** with NBu_4Cl

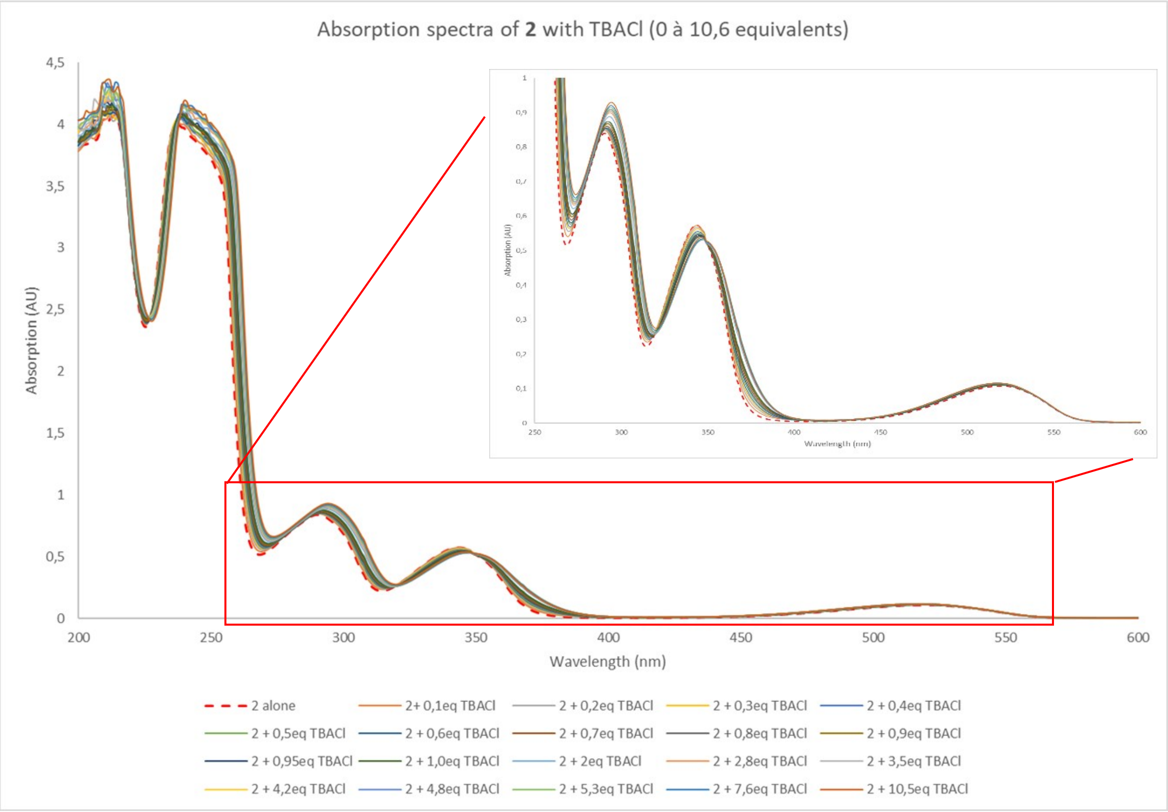

*Figure S29: Experimental UV-Visible spectra measured during the titration of **2** with NBu_4Cl (0 to 10,5 equivalents)*

[PROGRAM]
Name = SPECFIT
Version = 3.0

[FILE]
Name = HB16+TBACL_ABS_FORMAT_SPECFIT.FAC
Path = C:\Program Files\SPECFIT\DATA\
Date = 18-juil-19
Time = 18:41:04
Ncomp = 2
Nmeas = 20
Nwave = 501

[FACTOR ANALYSIS]
Tolerance = 1,000E-09
Max.Factors = 10
Num.Factors = 8
Significant = 3
Eigen Noise = 6,121E-03
Exp't Noise = 6,121E-03

#	Eigenvalue	Square Sum	Residual	Prediction
1	1,719E+04	4,685E+01	6,838E-02	Data Vector
2	4,532E+01	1,533E+00	1,237E-02	Data Vector
3	1,158E+00	3,753E-01	6,121E-03	Data Vector
4	1,229E-01	2,524E-01	5,020E-03	Possibly Data
5	8,254E-02	1,698E-01	4,118E-03	Probably Noise
6	3,956E-02	1,303E-01	3,607E-03	Probably Noise
7	2,791E-02	1,024E-01	3,197E-03	Probably Noise
8	1,898E-02	8,338E-02	2,886E-03	Probably Noise

[MODEL]
Date = 18-juil-19
Time = 18:41:32
Model = 0
Index = 3
Function = 1
Species = 3
Params = 3

[SPECIES]	[COLORED]	[FIXED]	[SPECTRUM]
1 0 0	False	False	
0 1 0	True	False	
1 1 0	True	False	

[SPECIES]	[FIXED]	[PARAMETER]		[ERROR]
1 0 0	True	0,00000E+00	+/-	0,00000E+00
0 1 0	True	0,00000E+00	+/-	0,00000E+00
1 1 0	False	4,07883E+00	+/-	7,70214E-02

[CONVERGENCE]
Iterations = 3
Convergence Limit = 1,000E-03
Convergence Found = 1,384E-05
Marquardt Parameter = 0,0
Sum(Y-y)^2 Residuals = 1,85499E+00
Std. Deviation of Fit(Y) = 1,36069E-02

[STATISTICS]
Experimental Noise = 6,121E-03
Relative Error Of Fit = 1,0375%
Durbin-Watson Factor = 0,5055
Goodness Of Fit, Chi^2 = 4,941E+00
Durbin-Watson Factor (raw data) = None
Goodness Of Fit, Chi^2 (raw data) = None

[COVARIANCE]
3,765E-02

[CORRELATION]
1,000E+00

[END FILE]

*Figure S30: Determination of binding constant using SPECFIT software for the UV-Visible titration of **2** with tetrabutylammonium chlorid*

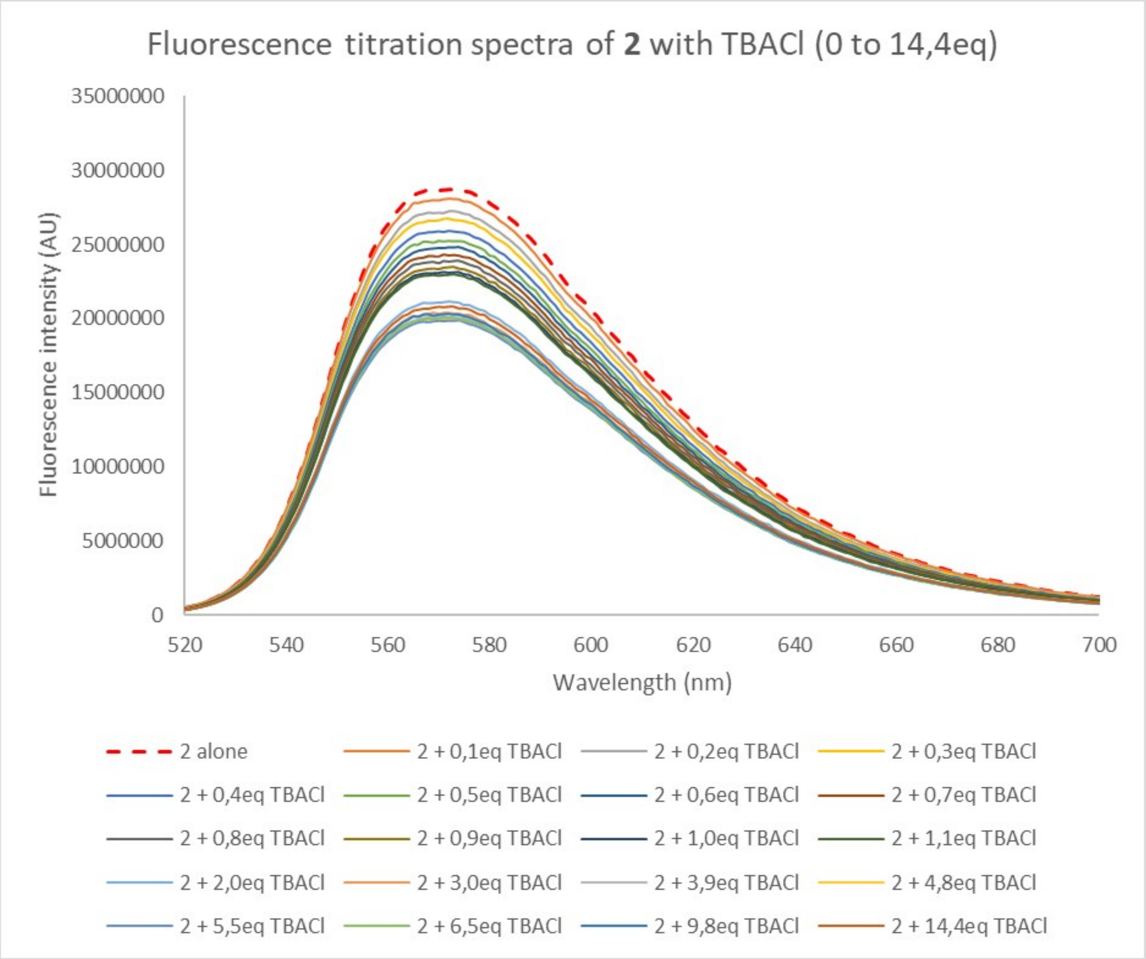

*Figure S31: Experimental fluorescence spectra during the titration of **2** with NBu_4Cl (0 to 14,4 equivalents)*

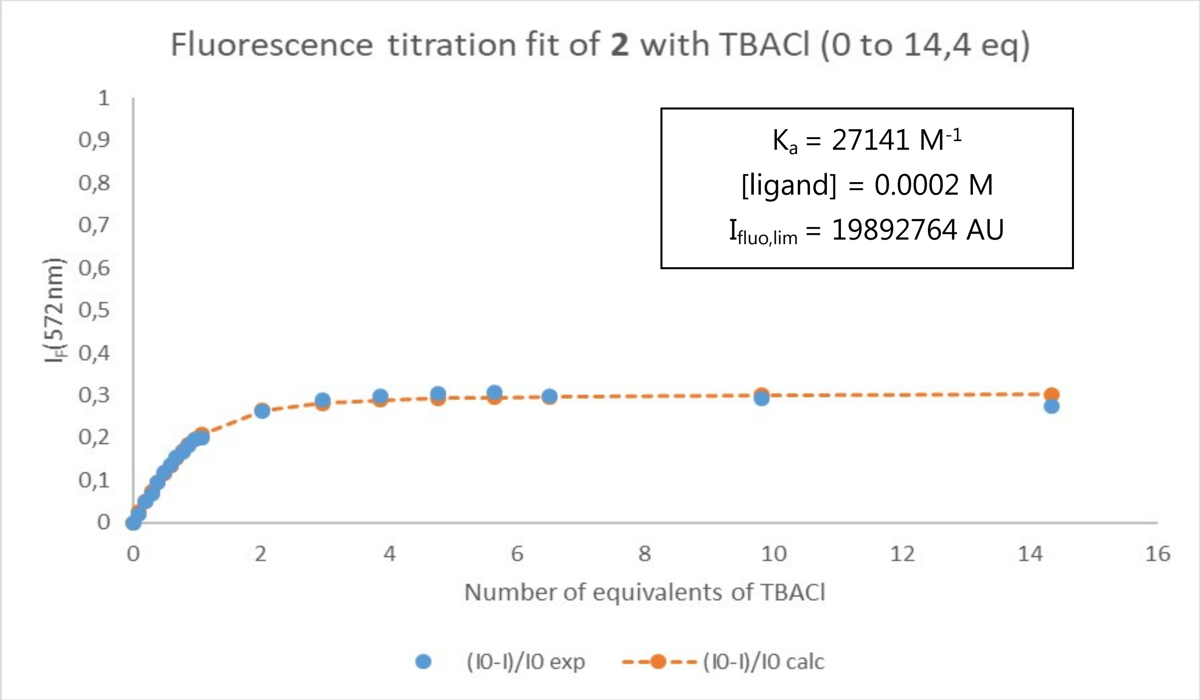

*Figure S32: Mathematical fit during the fluorescence titration of **2** with NBu_4Cl (0 to 70 equivalents) and determination of the association constant*

[PROGRAM]
Name = SPECFIT
Version = 3.0

[FILE]
Name = HB16+TBACL_FLUO_FORMAT_SPECFIT.FAC
Path = C:\Program Files\SPECFIT\DATA\
Date = 18-juil-19
Time = 18:47:25
Ncomp = 2
Nmeas = 20
Nwave = 281

[FACTOR ANALYSIS]
Tolerance = 1,000E-09
Max.Factors = 10
Num.Factors = 4
Significant = 2
Eigen Noise = 1,980E+04
Exp't Noise = 1,980E+04

#	Eigenvalue	Square Sum	Residual	Prediction
1	5,808E+17	2,871E+13	7,148E+04	Data Vector
2	2,650E+13	2,202E+12	1,980E+04	Data Vector
3	2,523E+11	1,950E+12	1,863E+04	Probably Noise
4	1,942E+11	1,755E+12	1,768E+04	Probably Noise

[MODEL]
Date = 18-juil-19
Time = 18:48:04
Model = 0
Index = 3
Function = 1
Species = 3
Params = 3

[SPECIES]	[COLORED]	[FIXED]	[SPECTRUM]
1 0 0	False	False	
0 1 0	True	False	
1 1 0	True	False	

[SPECIES]	[FIXED]	[PARAMETER]		[ERROR]
1 0 0	True	0,00000E+00	+/-	0,00000E+00
0 1 0	True	0,00000E+00	+/-	0,00000E+00
1 1 0	False	4,44539E+00	+/-	4,13210E-02

[CONVERGENCE]
Iterations = 9
Convergence Limit = 1,000E-03
Convergence Found = 1,571E-05
Marquardt Parameter = 0,0
Sum(Y-y)^2 Residuals = 5,94920E+13
Std. Deviation of Fit(Y) = 1,02896E+05

[STATISTICS]
Experimental Noise = 1,980E+04
Relative Error Of Fit = 1,0122%
Durbin-Watson Factor = 0,6396
Goodness Of Fit, Chi^2 = 2,701E+01
Durbin-Watson Factor (raw data) = None
Goodness Of Fit, Chi^2 (raw data) = None

[COVARIANCE]
9,964E-03

[CORRELATION]
1,000E+00

[END FILE]

*Figure S33: Determination of binding constant using SPECFIT software for the fluorescence titration of **2** with tetrabutylammonium chloride*

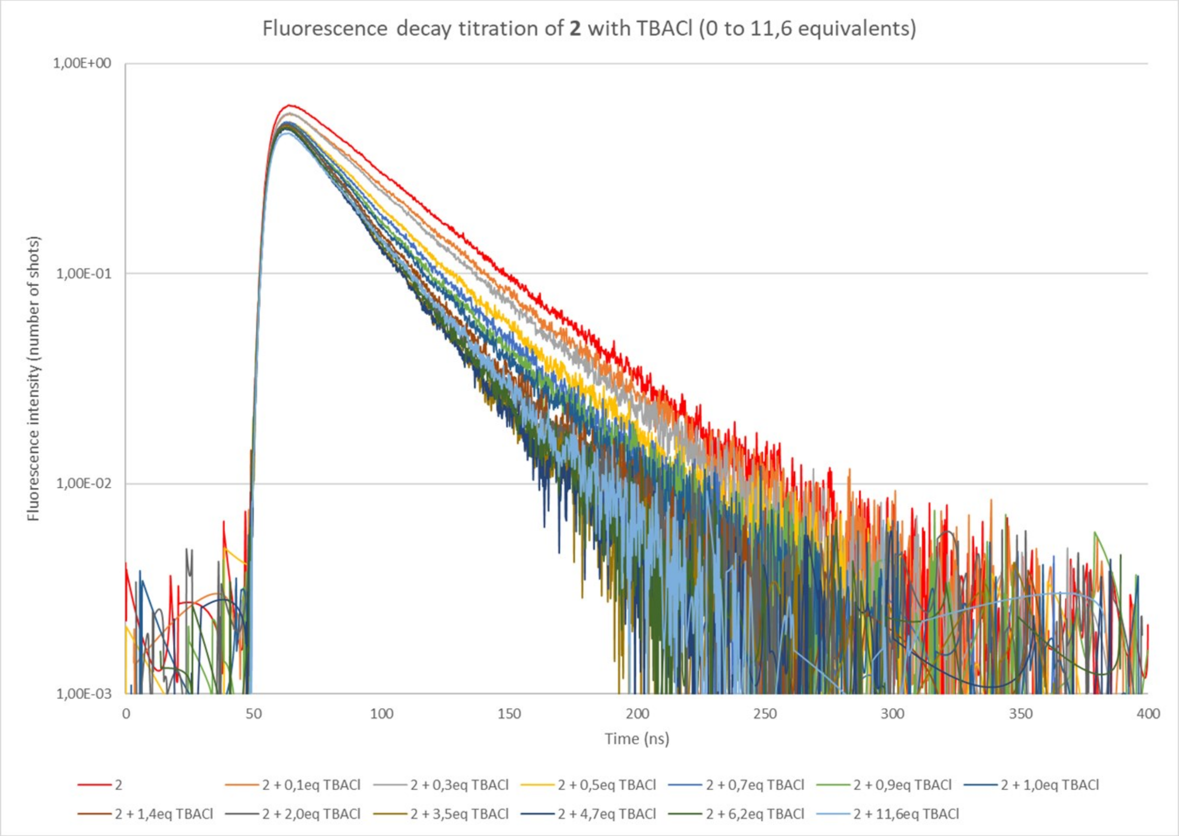

*Figure S34: Fluorescence decay titration of **2** with NBu_4Cl (0 to 11,6 equivalents)*
Logarithmic scale

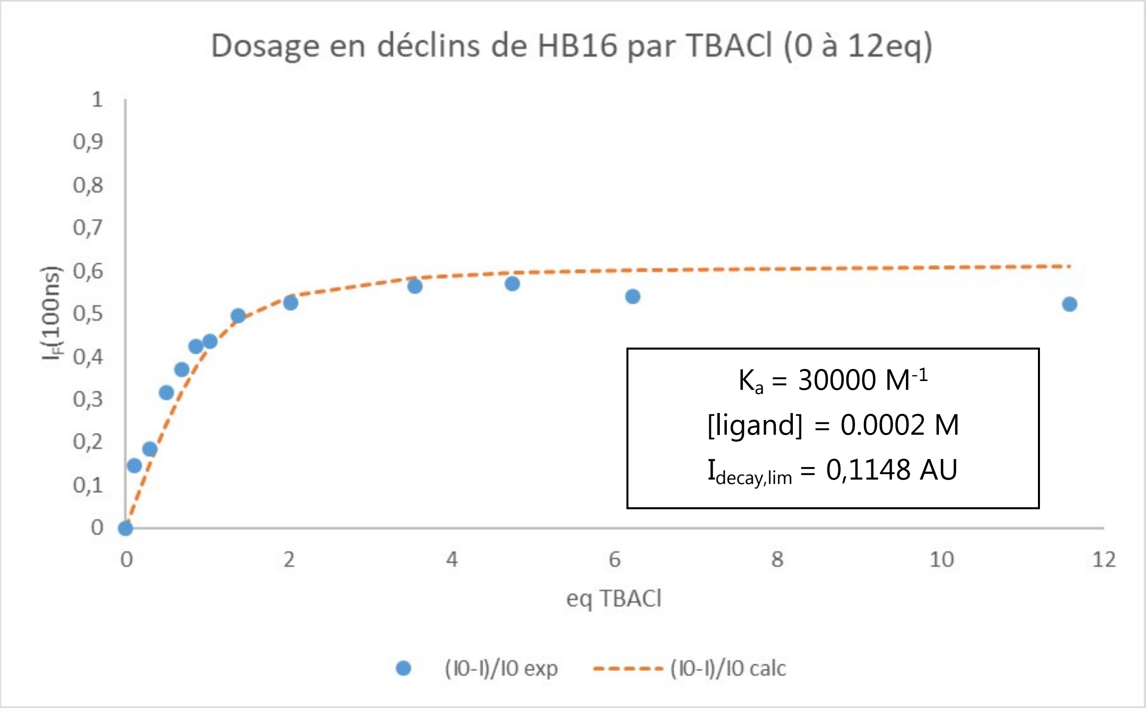

*Figure S35: Mathematical fit during the fluorescence decay titration of **2** with NBu_4Cl (0 to 11,6 equivalents) and determination of the association constant*

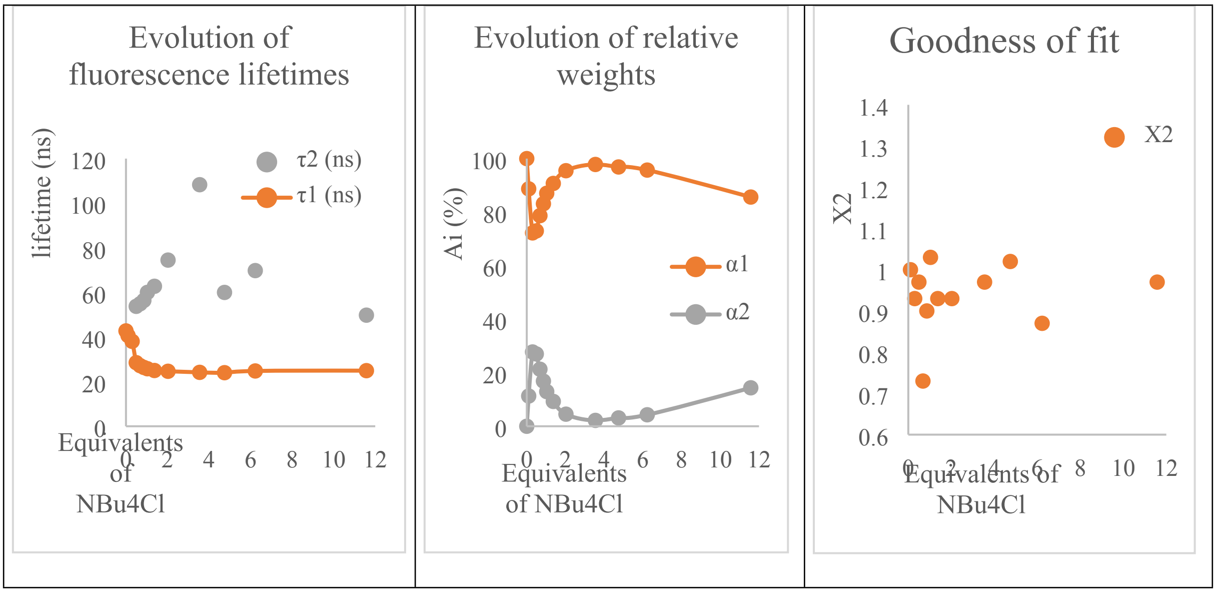

*Figure S36: Analysis of fluorescence decay titration of **2** with NBu_4Cl*

6.5 Titration of **2** with NBu_4Br

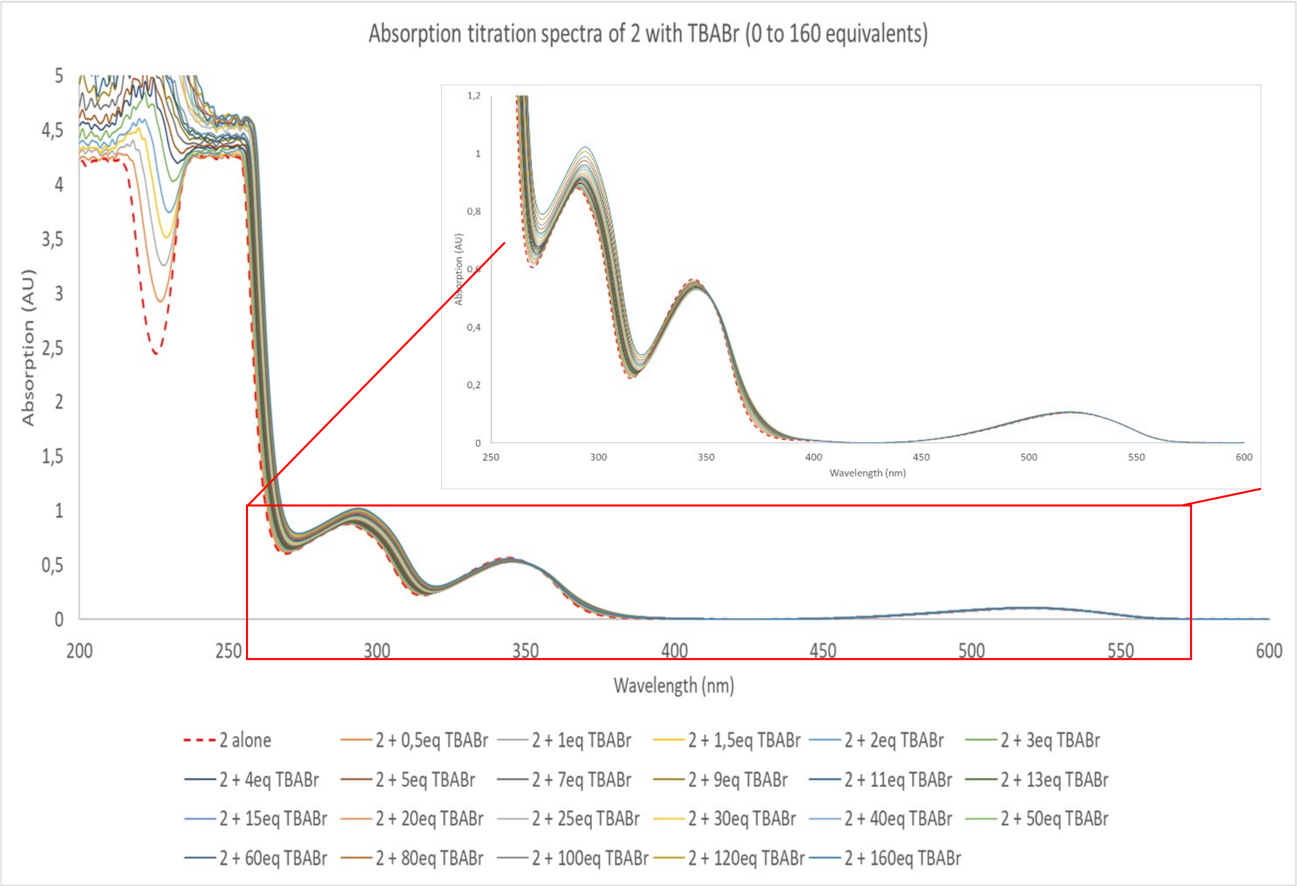

*Figure S37: Experimental UV-Visible spectra measured during the titration of **2** with NBu_4Br (0 to 160 equivalents)*

```
[PROGRAM]
Name = SPECFIT
Version = 3.0

[FILE]
Name = RP05+TBABR_ABS_FORMAT_SPECFIT C.FAC
Path = C:\Program Files\SPECFIT\DATA\
Date = 07-juil-20
Time = 11:23:33
Ncomp = 2
Nmeas = 23
Nwave = 426

[FACTOR ANALYSIS]
Tolerance = 1,000E-09
Max.Factors = 10
Num.Factors = 9
Significant = 4
Eigen Noise = 8,809E-05
Exp't Noise = 8,809E-05
 #  Eigenvalue  Square Sum  Residual   Prediction
 1  7,558E+02   1,674E+00   1,307E-02  Data Vector
 2  1,499E+00   1,747E-01   4,223E-03  Data Vector
 3  1,736E-01   1,088E-03   3,333E-04  Data Vector
 4  1,012E-03   7,601E-05   8,809E-05  Data Vector
 5  2,081E-05   5,519E-05   7,507E-05  Possibly Data
 6  1,104E-05   4,416E-05   6,715E-05  Probably Noise
 7  7,800E-06   3,636E-05   6,094E-05  Probably Noise
 8  4,847E-06   3,151E-05   5,673E-05  Probably Noise
 9  3,828E-06   2,768E-05   5,318E-05  Probably Noise

[MODEL]
Date = 07-juil-20
Time = 11:23:46
Model = 0
Index = 3
Function = 1
Species = 3
Params = 3

[SPECIES]       [COLORED]       [FIXED]         [SPECTRUM]
1 0 0           False           False
0 1 0           True            False
1 1 0           True            False

[SPECIES]       [FIXED]         [PARAMETER]         [ERROR]
1 0 0           True            0,00000E+00  +/-    0,00000E+00
0 1 0           True            0,00000E+00  +/-    0,00000E+00
1 1 0           False           2,29190E+00  +/-    8,78013E-02

[CONVERGENCE]
Iterations = 7
Convergence Limit = 1,000E-03
Convergence Found = 6,738E-04
Marquardt Parameter = 0,0
Sum(Y-y)^2 Residuals = 3,24482E-01
Std. Deviation of Fit(Y) = 5,75504E-03

[STATISTICS]
Experimental Noise = 8,809E-05
Relative Error Of Fit = 2,0702%
Durbin-Watson Factor = 0,1340
Goodness Of Fit, Chi^2 = 4,268E+03
Durbin-Watson Factor (raw data) = None
Goodness Of Fit, Chi^2 (raw data) = None

[COVARIANCE]
5,020E-02

[CORRELATION]
1,000E+00

[END FILE]
```

*Figure S38: Determination of binding constant using SPECFIT software for the UV-Visible titration of **1** with tetrabutylammonium bromide*

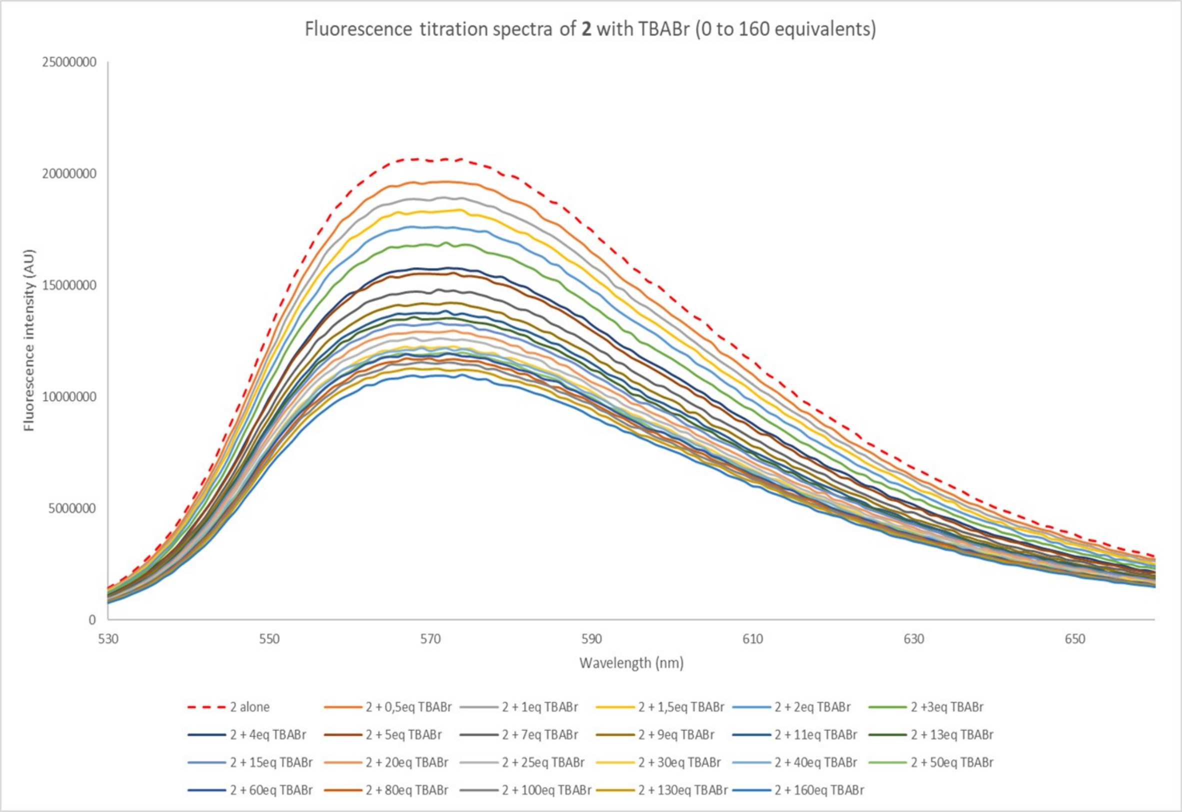

*Figure S39: Experimental fluorescence spectra during the titration of **2** with NBu_4Br (0 to 160 equivalents)*

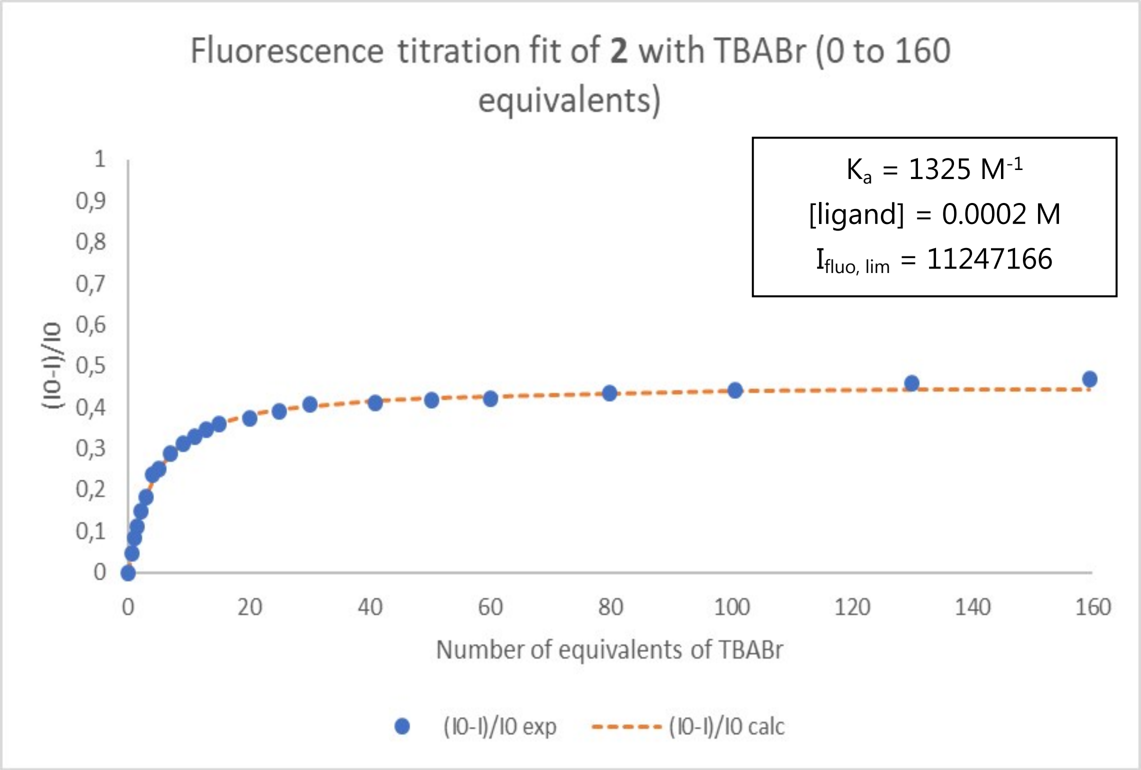

*Figure S40: Mathematical fit during the fluorescence titration of **2** with NBu_4Br (0 to 160 equivalents) and determination of the association constant*

[PROGRAM]
Name = SPECFIT
Version = 3.0

[FILE]
Name = RP05+TBABR_FLUO_FORMAT_SPECFIT.FAC
Path = C:\Program Files\SPECFIT\DATA\
Date = 07-juil-20
Time = 11:10:05
Ncomp = 2
Nmeas = 23
Nwave = 271

[FACTOR ANALYSIS]
Tolerance = 1,000E-09
Max.Factors = 10
Num.Factors = 4
Significant = 2
Eigen Noise = 2,175E+04
Exp't Noise = 2,175E+04

#	Eigenvalue	Square Sum	Residual	Prediction
1	2,565E+17	1,018E+13	4,041E+04	Data Vector
2	7,230E+12	2,947E+12	2,175E+04	Data Vector
3	3,204E+11	2,626E+12	2,053E+04	Probably Noise
4	2,462E+11	2,380E+12	1,955E+04	Probably Noise

[MODEL]
Date = 07-juil-20
Time = 11:10:27
Model = 0
Index = 3
Function = 1
Species = 3
Params = 3

[SPECIES]	[COLORED]	[FIXED]	[SPECTRUM]
1 0 0	False	False	
0 1 0	True	False	
1 1 0	True	False	

[SPECIES]	[FIXED]	[PARAMETER]		[ERROR]
1 0 0	True	0,00000E+00	+/-	0,00000E+00
0 1 0	True	0,00000E+00	+/-	0,00000E+00
1 1 0	False	3,10281E+00	+/-	1,36987E-02

[CONVERGENCE]
Iterations = 8
Convergence Limit = 1,000E-03
Convergence Found = 1,920E-05
Marquardt Parameter = 0,0
Sum(Y-y)^2 Residuals = 2,54364E+13
Std. Deviation of Fit(Y) = 6,38872E+04

[STATISTICS]
Experimental Noise = 2,175E+04
Relative Error Of Fit = 0,9959%
Durbin-Watson Factor = 0,9016
Goodness Of Fit, Chi^2 = 8,631E+00
Durbin-Watson Factor (raw data) = None
Goodness Of Fit, Chi^2 (raw data) = None

[COVARIANCE]
1,027E-03

[CORRELATION]
1,000E+00

[END FILE]

*Figure S41: Determination of binding constant using SPECFIT software for the fluorescence titration of **2** with tetrabutylammonium bromide*

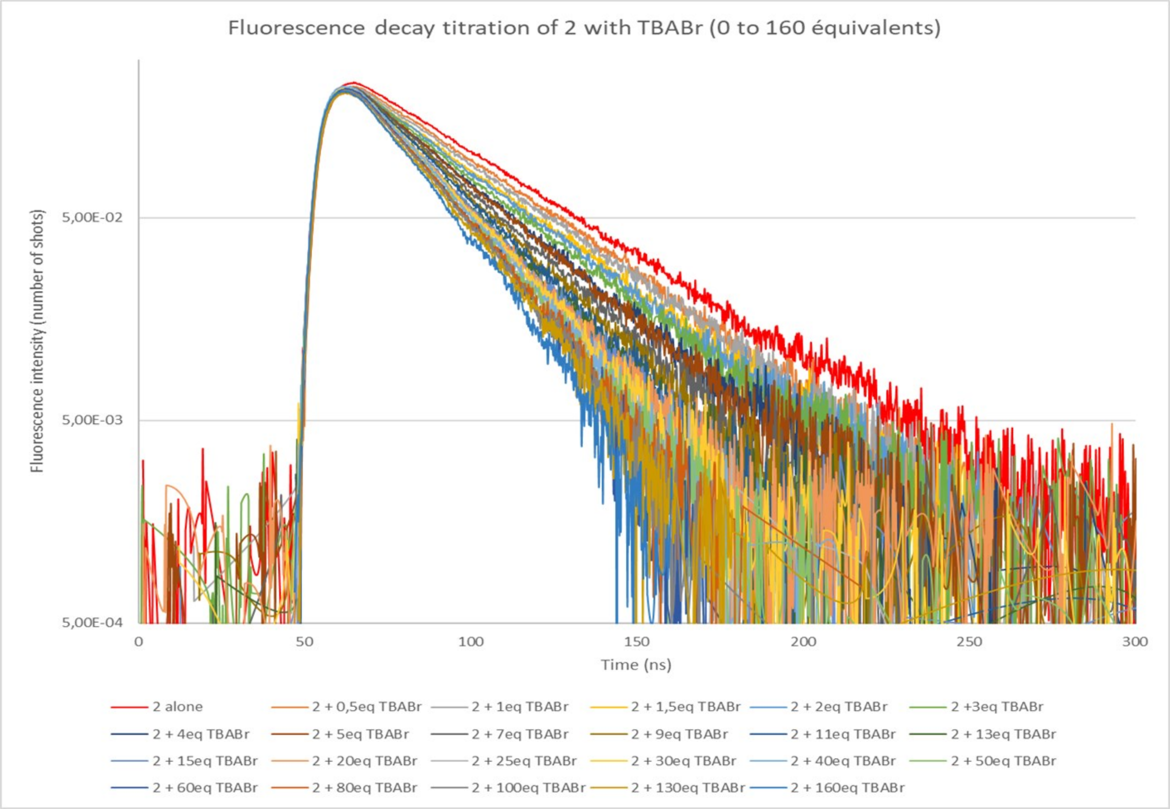

*Figure S42: Fluorescence decay titration of **2** with tetrabutylammonium bromide (0 to 160 equivalents) Logarithmic scale*

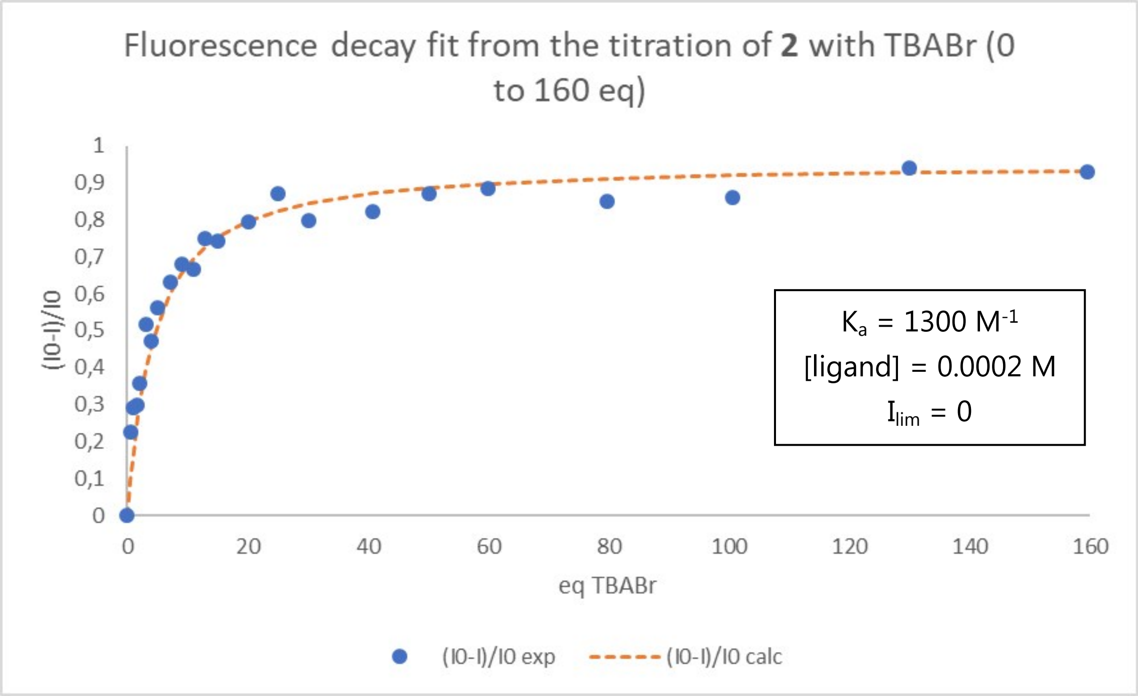

*Figure S43: Mathematical fit during the fluorescence decay titration of **2** with NBu_4Br (0 to 160 equivalents) and determination of the association constant*

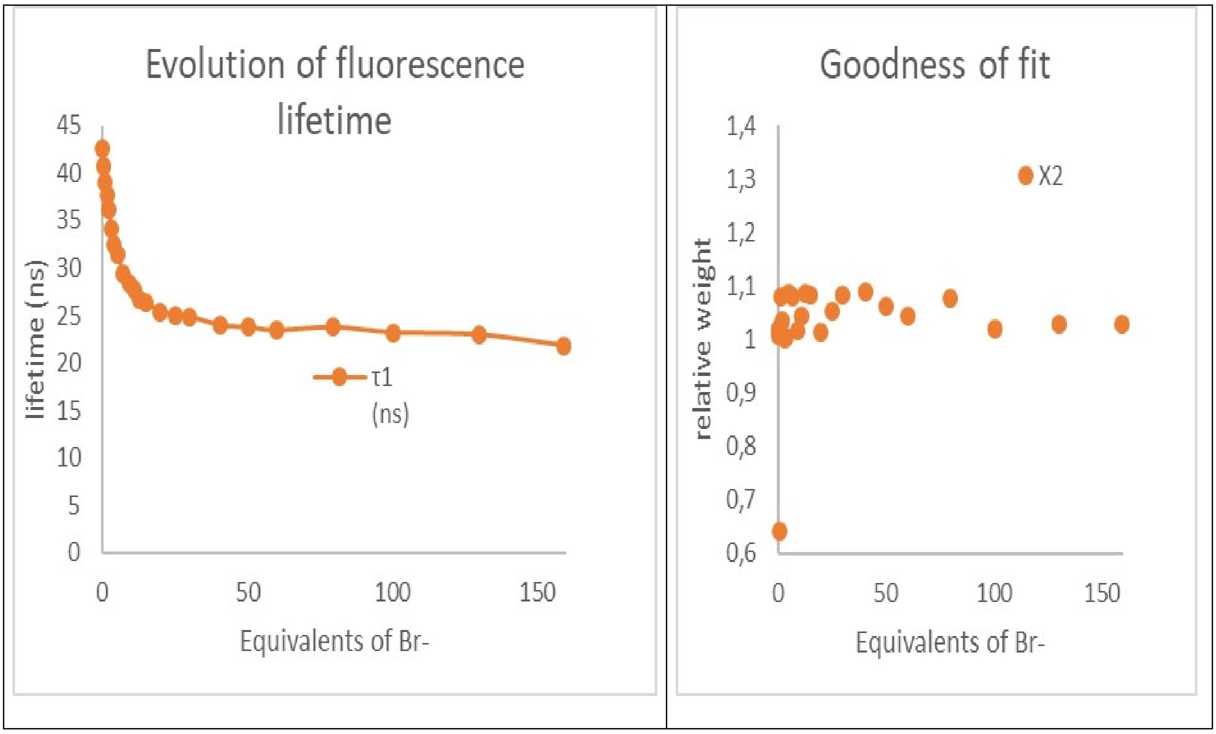

*Figure S44: Analysis of fluorescence decay titration of **2** with tetrabutylammonium bromide*

6.6 Titration of 2 with NBu_4I

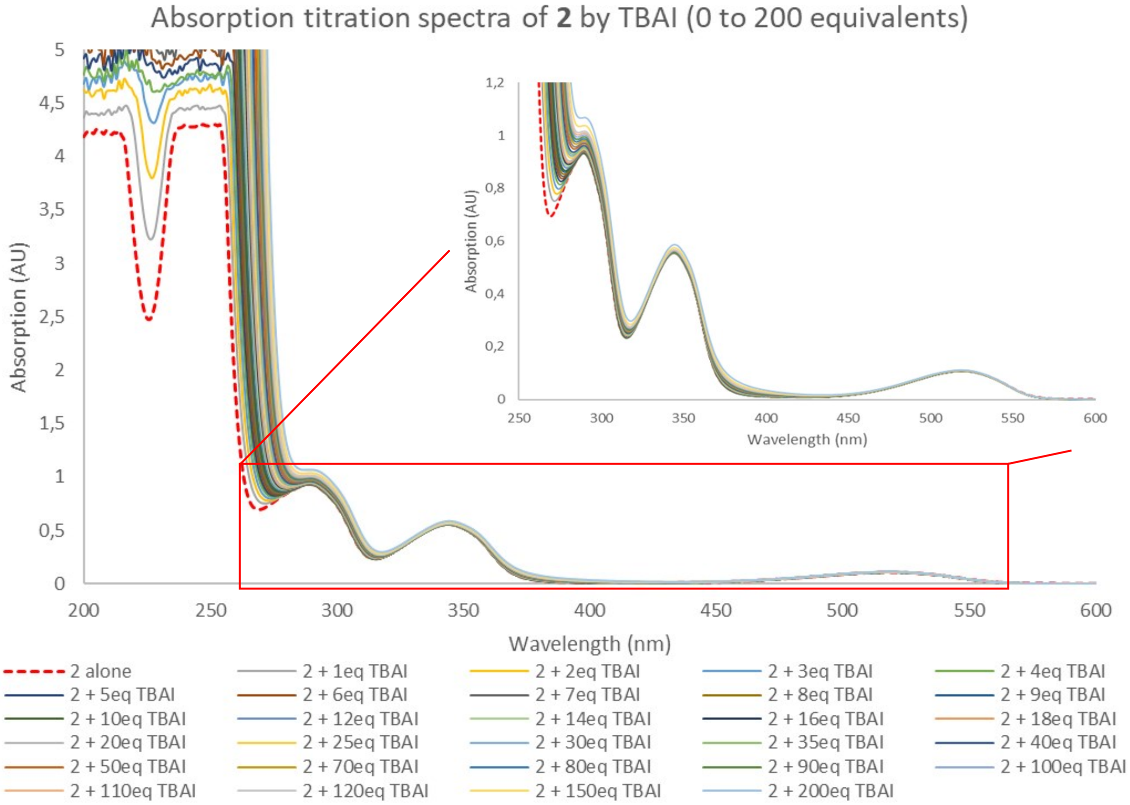

*Figure S45: Experimental UV-Visible spectra measured during the titration of **2** with NBu_4I (0 to 200 equivalents)*

[PROGRAM]
Name = SPECFIT
Version = 3.0

[FILE]
Name = RP05+TBAI_ABS_FORMAT_SPECFITC.FAC
Path = C:\Program Files\SPECFIT\DATA\
Date = 07-juil-20
Time = 11:17:56
Ncomp = 2
Nmeas = 30
Nwave = 426

[FACTOR ANALYSIS]
Tolerance = 1,000E-09
Max.Factors = 10
Num.Factors = 10
Significant = 5
Eigen Noise = 1,035E-04
Exp't Noise = 1,035E-04

#	Eigenvalue	Square Sum	Residual	Prediction
1	1,131E+03	1,265E+01	3,147E-02	Data Vector
2	1,263E+01	1,861E-02	1,207E-03	Data Vector
3	1,734E-02	1,273E-03	3,156E-04	Data Vector
4	9,513E-04	3,213E-04	1,586E-04	Data Vector
5	1,846E-04	1,367E-04	1,035E-04	Data Vector
6	4,366E-05	9,307E-05	8,536E-05	Possibly Data
7	2,526E-05	6,781E-05	7,286E-05	Probably Noise
8	1,286E-05	5,495E-05	6,559E-05	Probably Noise
9	8,867E-06	4,608E-05	6,007E-05	Probably Noise
10	5,315E-06	4,077E-05	5,650E-05	Probably Noise

[MODEL]
Date = 07-juil-20
Time = 11:18:15
Model = 0
Index = 3
Function = 1
Species = 3
Params = 3

[SPECIES]	[COLORED]	[FIXED]	[SPECTRUM]
1 0 0	False	False	
0 1 0	True	False	
1 1 0	True	False	

[SPECIES]	[FIXED]	[PARAMETER]		[ERROR]
1 0 0	True	0,00000E+00	+/-	0,00000E+00
0 1 0	True	0,00000E+00	+/-	0,00000E+00
1 1 0	False	8,75297E-01	+/-	3,18841E-02

[CONVERGENCE]
Iterations = 15
Convergence Limit = 1,000E-03
Convergence Found = 4,523E-05
Marquardt Parameter = 0,0
Sum(Y-y)^2 Residuals = 2,90083E-02
Std. Deviation of Fit(Y) = 1,50665E-03

[STATISTICS]
Experimental Noise = 1,035E-04
Relative Error Of Fit = 0,5036%
Durbin-Watson Factor = 0,3295
Goodness Of Fit, Chi^2 = 2,121E+02
Durbin-Watson Factor (raw data) = None
Goodness Of Fit, Chi^2 (raw data) = None

[COVARIANCE]
5,803E-03

[CORRELATION]
1,000E+00

[END FILE]

*Figure S46: Determination of binding constant using SPECFIT software for the UV-Visible titration of **2** with tetrabutylammonium iodide*

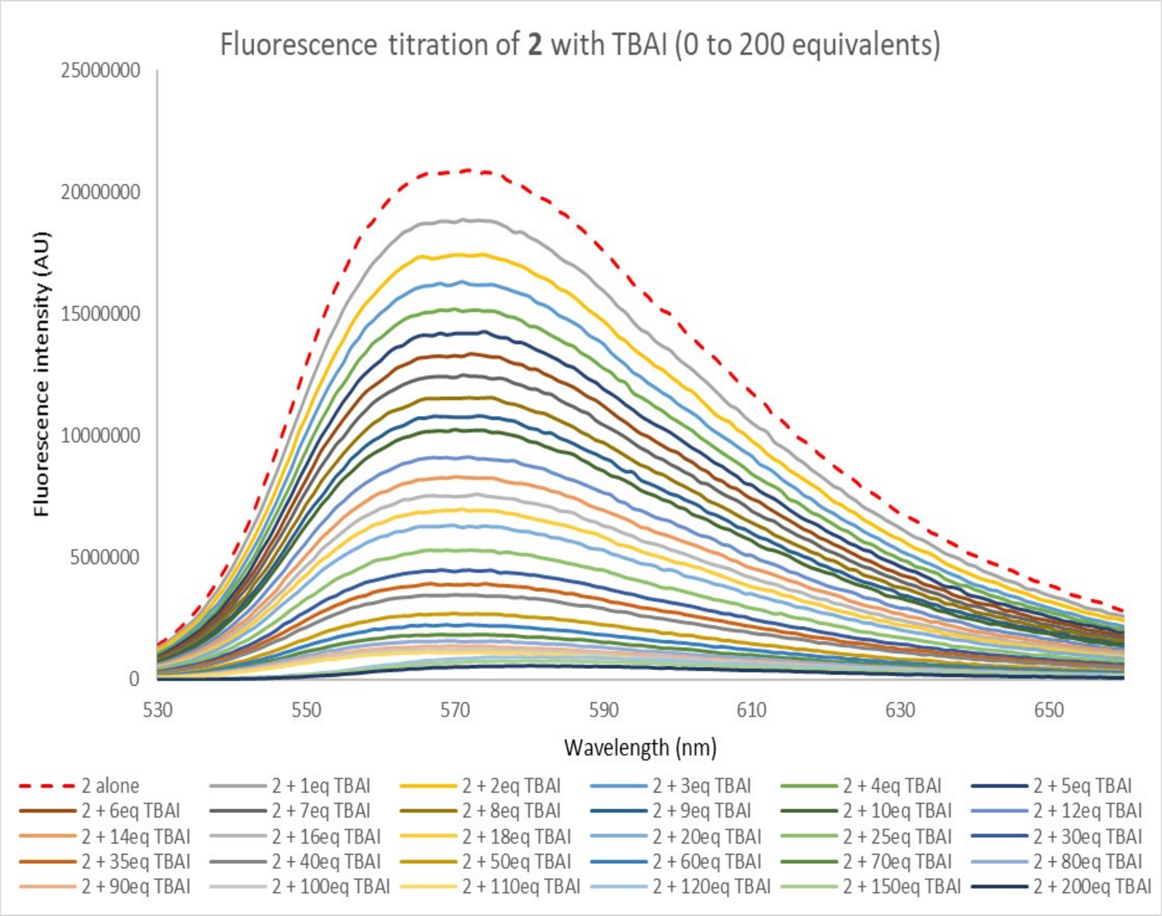

*Figure S47: Experimental fluorescence spectra during the titration of **2** with NBu_4I (0 to 200 equivalents)*

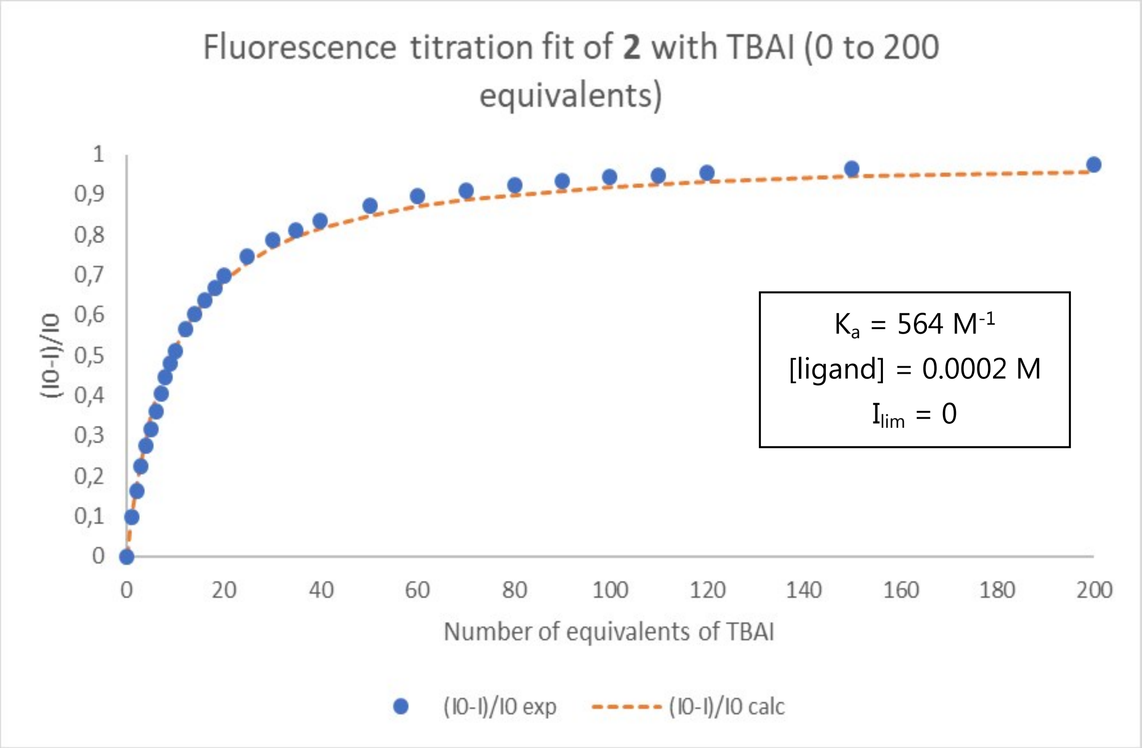

*Figure S48: Mathematical fit during the fluorescence titration of **2** with NBu_4I (0 to 200 equivalents) and determination of the association constant*

[PROGRAM]
Name = SPECFIT
Version = 3.0

[FILE]
Name = RP05+TBAI_FLUO_FORMAT_SPECFIT.FAC
Path = C:\Program Files\SPECFIT\DATA\
Date = 07-juil-20
Time = 11:25:33
Ncomp = 2
Nmeas = 30
Nwave = 271

[FACTOR ANALYSIS]
Tolerance = 1,000E-09
Max.Factors = 10
Num.Factors = 4
Significant = 2
Eigen Noise = 1,642E+04
Exp't Noise = 1,642E+04

#	Eigenvalue	Square Sum	Residual	Prediction
1	1,482E+17	8,461E+12	3,226E+04	Data Vector
2	6,269E+12	2,192E+12	1,642E+04	Data Vector
3	3,398E+11	1,852E+12	1,510E+04	Probably Noise
4	1,937E+11	1,659E+12	1,429E+04	Probably Noise

[MODEL]
Date = 07-juil-20
Time = 11:26:05
Model = 0
Index = 3
Function = 1
Species = 3
Params = 3

[SPECIES]	[COLORED]		[FIXED]	[SPECTRUM]
1 0 0	False		False	
0 1 0	True	False		
1 1 0	True	False		

[SPECIES]	[FIXED]		[PARAMETER]		[ERROR]
1 0 0	True	0,00000E+00 +/-	0,00000E+00		
0 1 0	True	0,00000E+00 +/-	0,00000E+00		
1 1 0	False		2,71082E+00	+/-	6,05215E-03

[CONVERGENCE]
Iterations = 8
Convergence Limit = 1,000E-03
Convergence Found = 5,201E-05
Marquardt Parameter = 0,0
Sum(Y-y)^2 Residuals = 4,09197E+13
Std. Deviation of Fit(Y) = 7,09493E+04

[STATISTICS]
Experimental Noise = 1,642E+04
Relative Error Of Fit = 1,6620%
Durbin-Watson Factor = 0,3350
Goodness Of Fit, Chi^2 = 1,866E+01
Durbin-Watson Factor (raw data) = None
Goodness Of Fit, Chi^2 (raw data) = None

[COVARIANCE]
1,969E-04

[CORRELATION]
1,000E+00

[END FILE]

*Figure S49: Determination of binding constant using SPECFIT software for the fluorescence titration of **2** with tetrabutylammonium iodide*

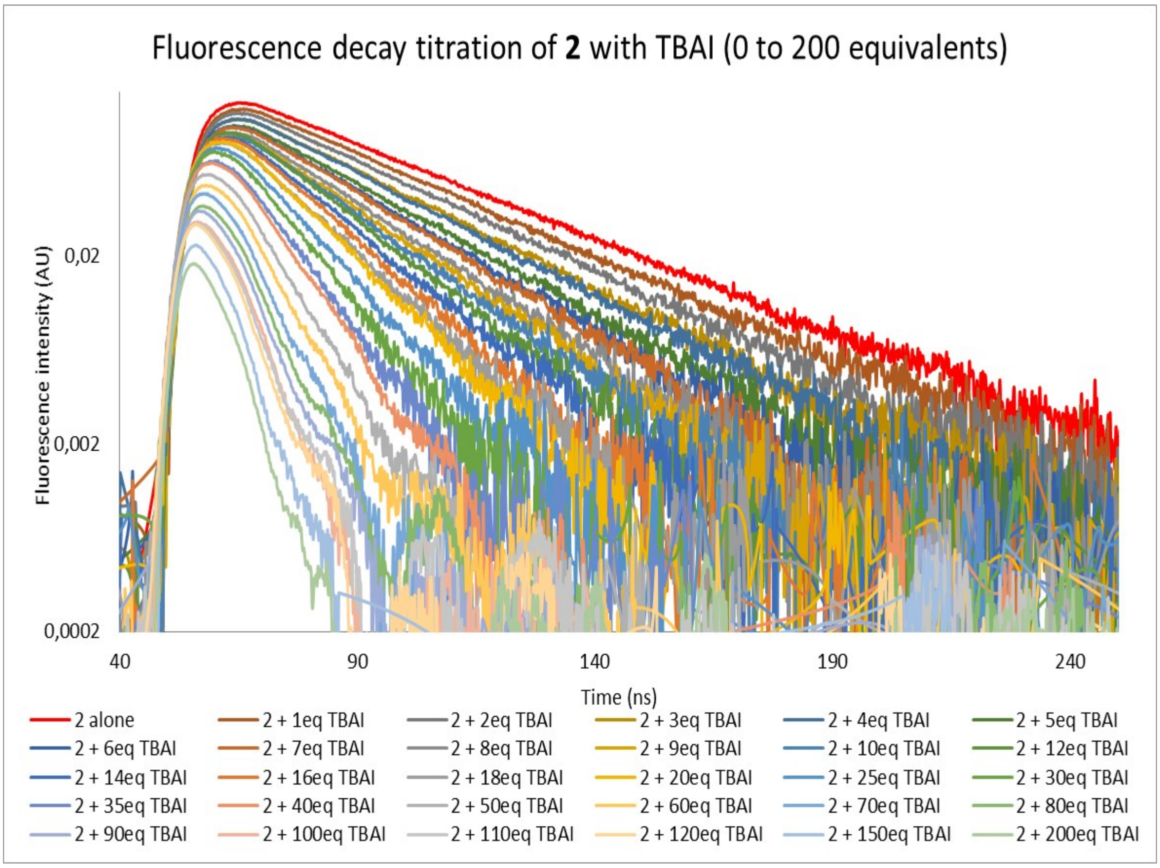

*Figure S50: Fluorescence decay titration of **2** with tetrabutylammonium iodide (0 to 200 equivalents) Logarithmic scale*

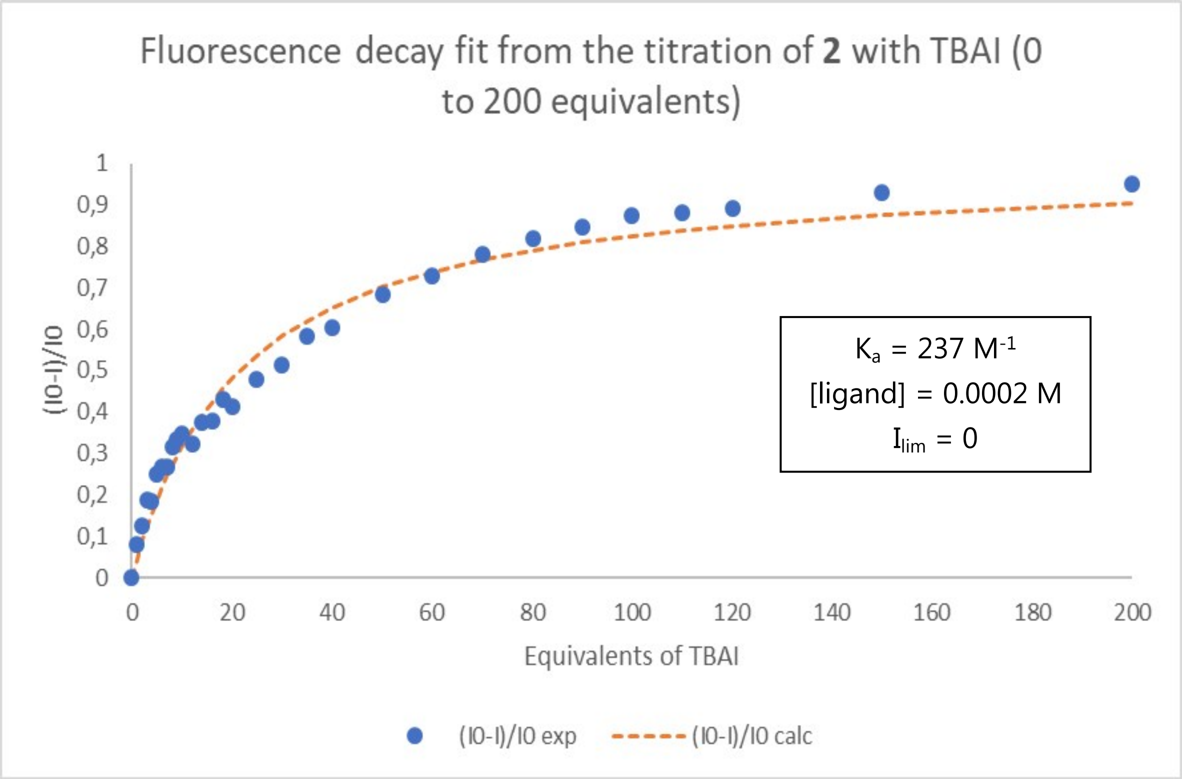

*Figure S51: Mathematical fit during the fluorescence decay titration of **2** with NBu_4I (0 to 200 equivalents) and determination of the association constant*

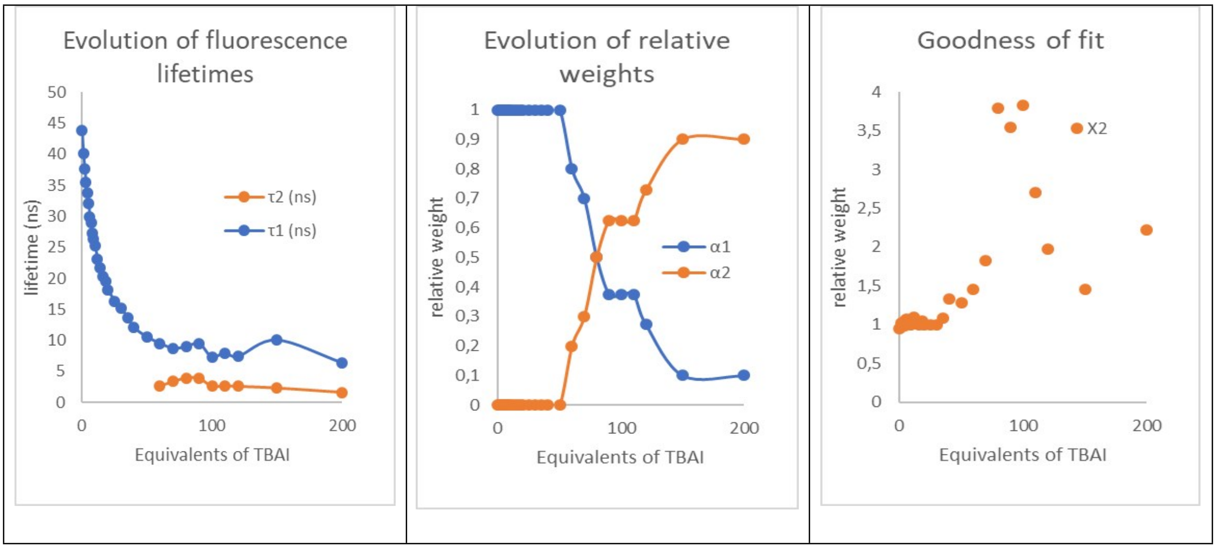

*Figure S52: Analysis of fluorescence decay titration of **2** with tetrabutylammonium iodide*

6.7 Titration of <u>2</u> with NBu_4SCN

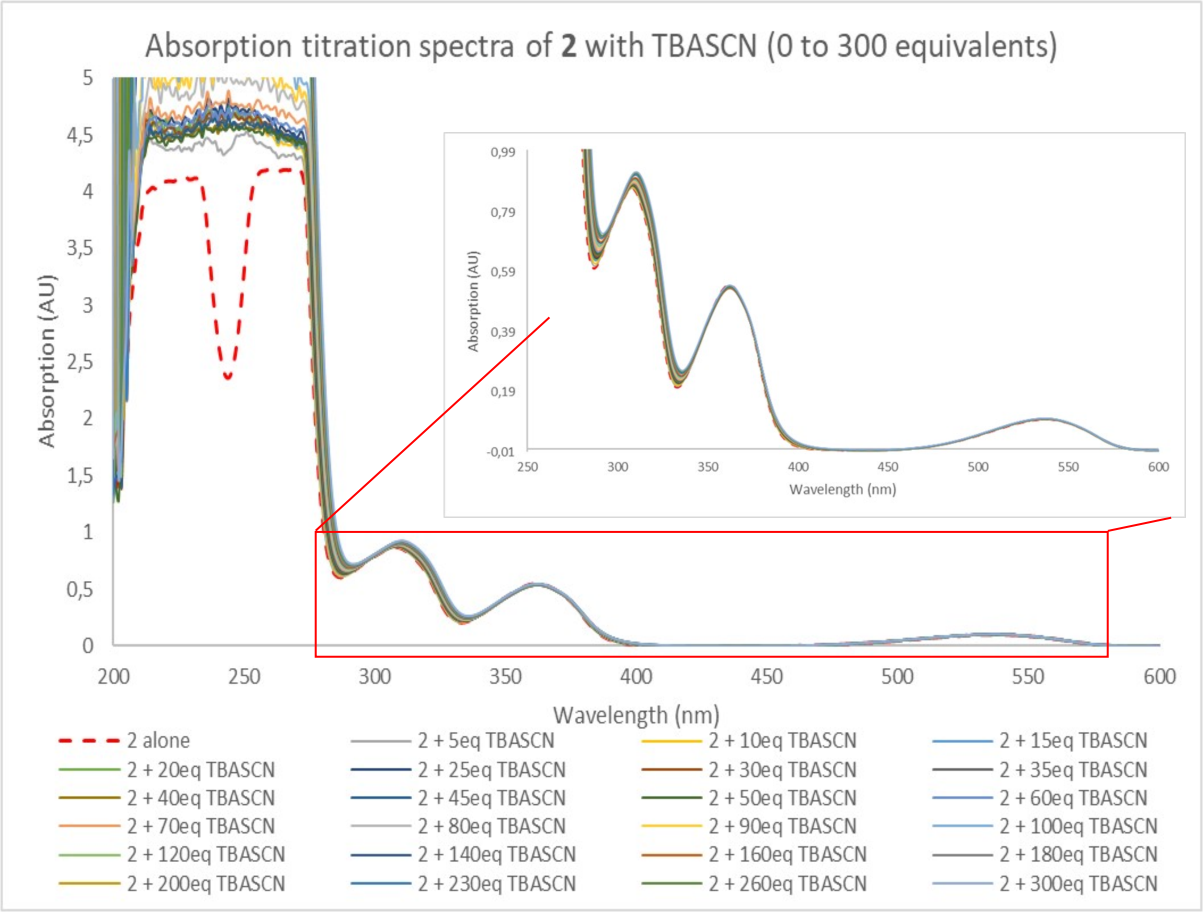

*Figure S53: Experimental UV-Visible spectra measured during the titration of **2** with NBu_4SCN (0 to 300 equivalents)*

[PROGRAM]
Name = SPECFIT
Version = 3.0

[FILE]
Name = RP05+TBASCN_ABS_FORMAT_SPECFIT C.FAC
Path = C:\Program Files\SPECFIT\DATA\
Date = 07-juil-20
Time = 11:36:26
Ncomp = 2
Nmeas = 24
Nwave = 421

[FACTOR ANALYSIS]
Tolerance = 1,000E-09
Max.Factors = 10
Num.Factors = 9
Significant = 5
Eigen Noise = 1,077E-04
Exp't Noise = 1,077E-04

#	Eigenvalue	Square Sum	Residual	Prediction
1	1,039E+03	9,196E+00	3,017E-02	Data Vector
2	9,179E+00	1,761E-02	1,320E-03	Data Vector
3	1,510E-02	2,507E-03	4,982E-04	Data Vector
4	2,195E-03	3,120E-04	1,758E-04	Data Vector
5	1,950E-04	1,171E-04	1,077E-04	Data Vector
6	3,905E-05	7,804E-05	8,791E-05	Possibly Data
7	1,883E-05	5,920E-05	7,657E-05	Probably Noise
8	1,500E-05	4,421E-05	6,617E-05	Probably Noise
9	6,365E-06	3,784E-05	6,122E-05	Probably Noise

[MODEL]
Date = 07-juil-20
Time = 11:36:46
Model = 0
Index = 3
Function = 1
Species = 3
Params = 3

[SPECIES]	[COLORED]	[FIXED]	[SPECTRUM]
1 0 0	False	False	
0 1 0	True	False	
1 1 0	True	False	

[SPECIES]	[FIXED]	[PARAMETER]		[ERROR]
1 0 0	True	0,00000E+00	+/-	0,00000E+00
0 1 0	True	0,00000E+00	+/-	0,00000E+00
1 1 0	False	1,26492E+00	+/-	2,21780E-02

[CONVERGENCE]
Iterations = 6
Convergence Limit = 1,000E-03
Convergence Found = 5,990E-05
Marquardt Parameter = 0,0
Sum(Y-y)^2 Residuals = 3,62219E-02
Std. Deviation of Fit(Y) = 1,89348E-03

[STATISTICS]
Experimental Noise = 1,077E-04
Relative Error Of Fit = 0,5878%
Durbin-Watson Factor = 0,4814
Goodness Of Fit, Chi^2 = 3,092E+02
Durbin-Watson Factor (raw data) = None
Goodness Of Fit, Chi^2 (raw data) = None

[COVARIANCE]
2,745E-03

[CORRELATION]
1,000E+00

[END FILE]

*Figure S54: Determination of binding constant using SPECFIT software for the UV-Visible titration of **2** with tetrabutylammonium thiocyanate*

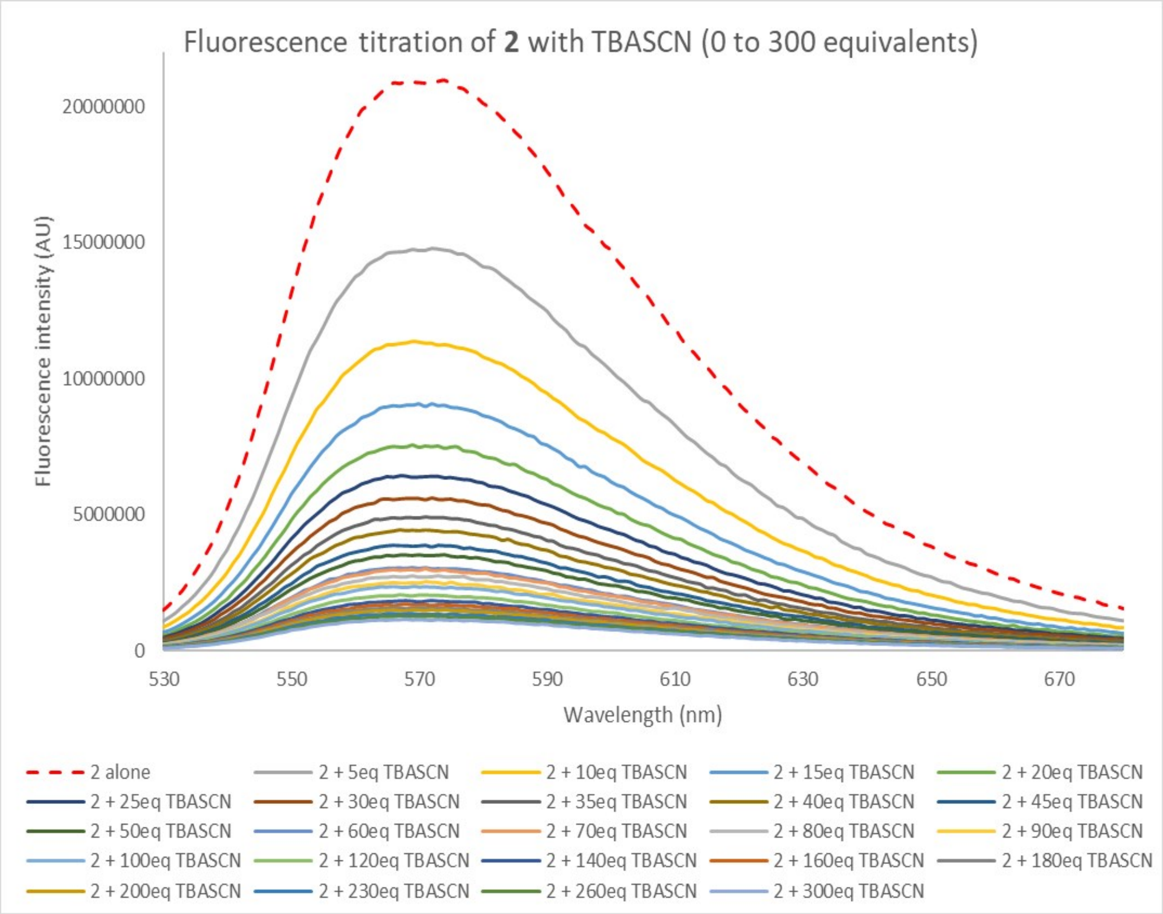

*Figure S55: Experimental fluorescence spectra during the titration of **2** with NBu_4SCN (0 to 300 equivalents)*

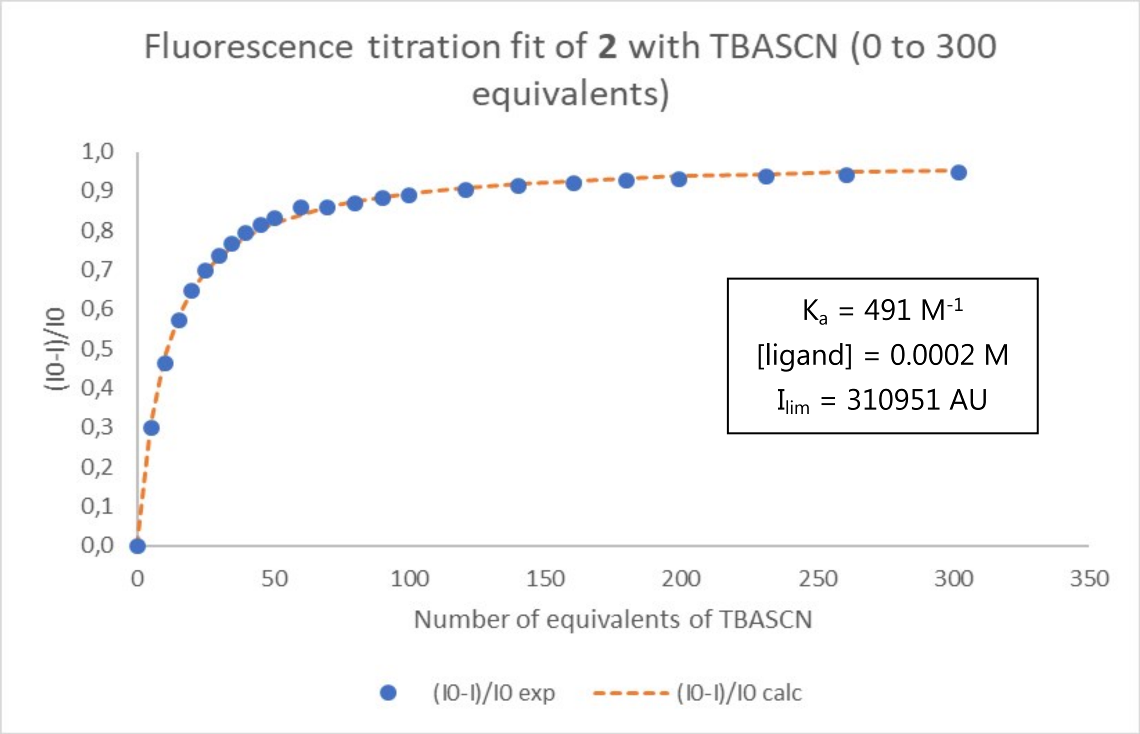

*Figure S56: Mathematical fit during the fluorescence titration of **2** with NBu_4SCN (0 to 300 equivalents) and determination of the association constant*

[PROGRAM]
Name = SPECFIT
Version = 3.0

[FILE]
Name = RP05+TBASCN_FLUO_FORMAT_SPECFIT.FAC
Path = C:\Program Files\SPECFIT\DATA\
Date = 07-juil-20
Time = 11:31:34
Ncomp = 2
Nmeas = 24
Nwave = 271

[FACTOR ANALYSIS]
Tolerance = 1,000E-09
Max.Factors = 10
Num.Factors = 5
Significant = 2
Eigen Noise = 1,248E+04
Exp't Noise = 1,248E+04

#	Eigenvalue	Square Sum	Residual	Prediction
1	5,819E+16	3,613E+12	2,357E+04	Data Vector
2	2,601E+12	1,012E+12	1,248E+04	Data Vector
3	1,680E+11	8,439E+11	1,139E+04	Probably Noise
4	1,328E+11	7,112E+11	1,046E+04	Probably Noise
5	8,539E+10	6,258E+11	9,813E+03	Probably Noise

[MODEL]
Date = 07-juil-20
Time = 11:31:49
Model = 0
Index = 3
Function = 1
Species = 3
Params = 3

[SPECIES]	[COLORED]	[FIXED]	[SPECTRUM]
1 0 0	False	False	
0 1 0	True	False	
1 1 0	True	False	

[SPECIES]	[FIXED]	[PARAMETER]		[ERROR]
1 0 0	True	0,00000E+00	+/-	0,00000E+00
0 1 0	True	0,00000E+00	+/-	0,00000E+00
1 1 0	False	2,69825E+00	+/-	7,81192E-03

[CONVERGENCE]
Iterations = 6
Convergence Limit = 1,000E-03
Convergence Found = 7,481E-04
Marquardt Parameter = 0,0
Sum(Y-y)^2 Residuals = 3,73201E+13
Std. Deviation of Fit(Y) = 7,57555E+04

[STATISTICS]
Experimental Noise = 1,248E+04
Relative Error Of Fit = 2,5333%
Durbin-Watson Factor = 0,4869
Goodness Of Fit, Chi^2 = 3,687E+01
Durbin-Watson Factor (raw data) = None
Goodness Of Fit, Chi^2 (raw data) = None

[COVARIANCE]
3,294E-04

[CORRELATION]
1,000E+00

[END FILE]

*Figure S57: Determination of binding constant using SPECFIT software for the fluorescence titration of **2** with tetrabutylammonium thioisocyanate*

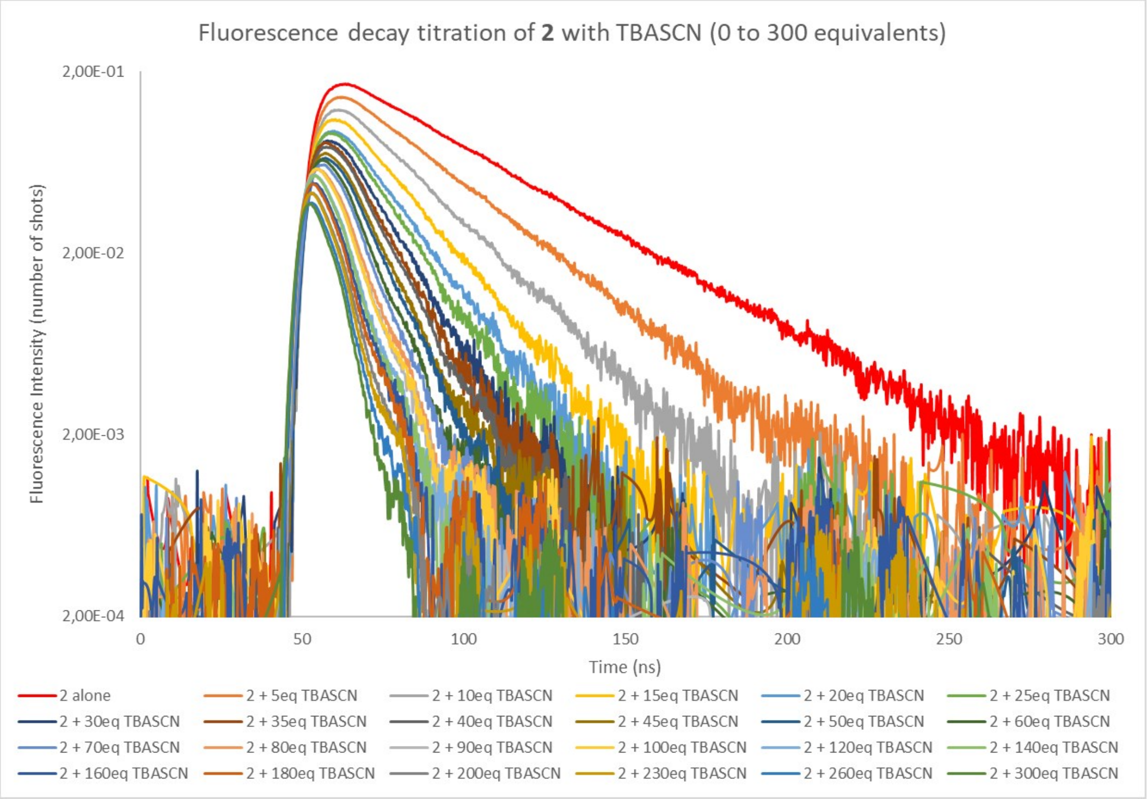

*Figure S58: Fluorescence decay titration of **2** with tetrabutylammonium thiocyanate (0 to 300 equivalents) Logarithmic scale*

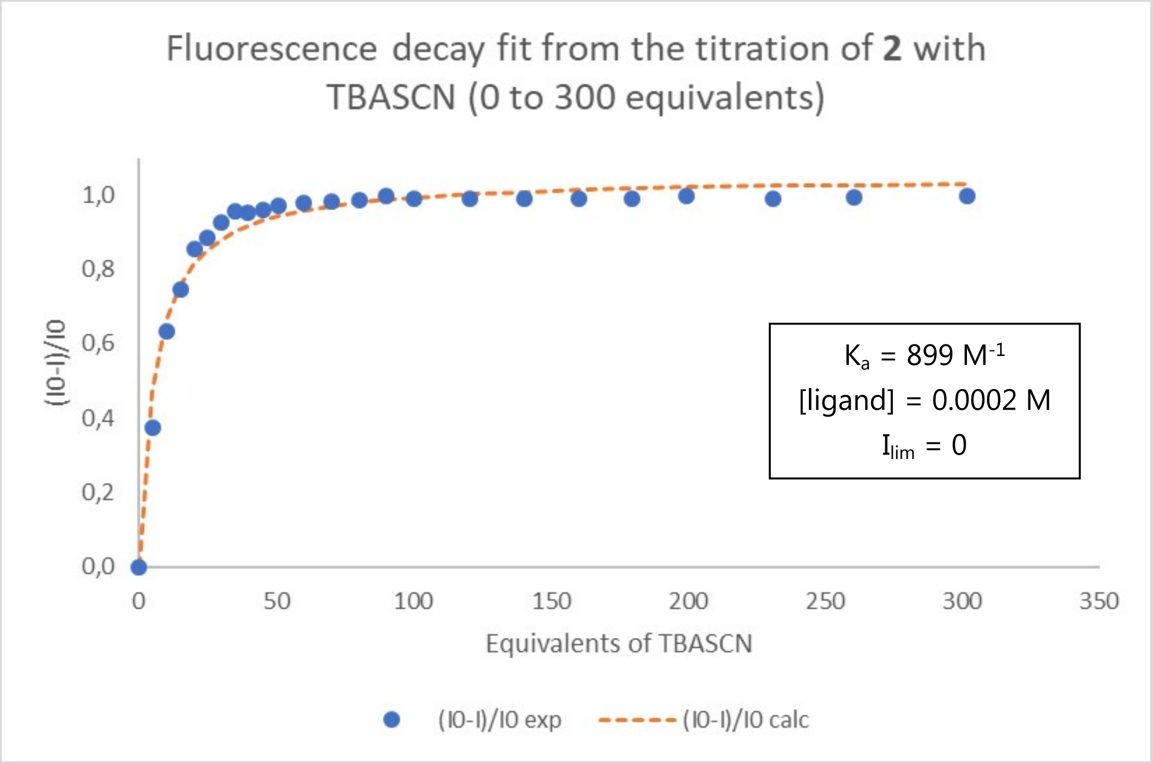

*Figure S59: Mathematical fit during the fluorescence decay titration of **2** with NBu_4SCN (0 to 300 equivalents) and determination of the association constant*

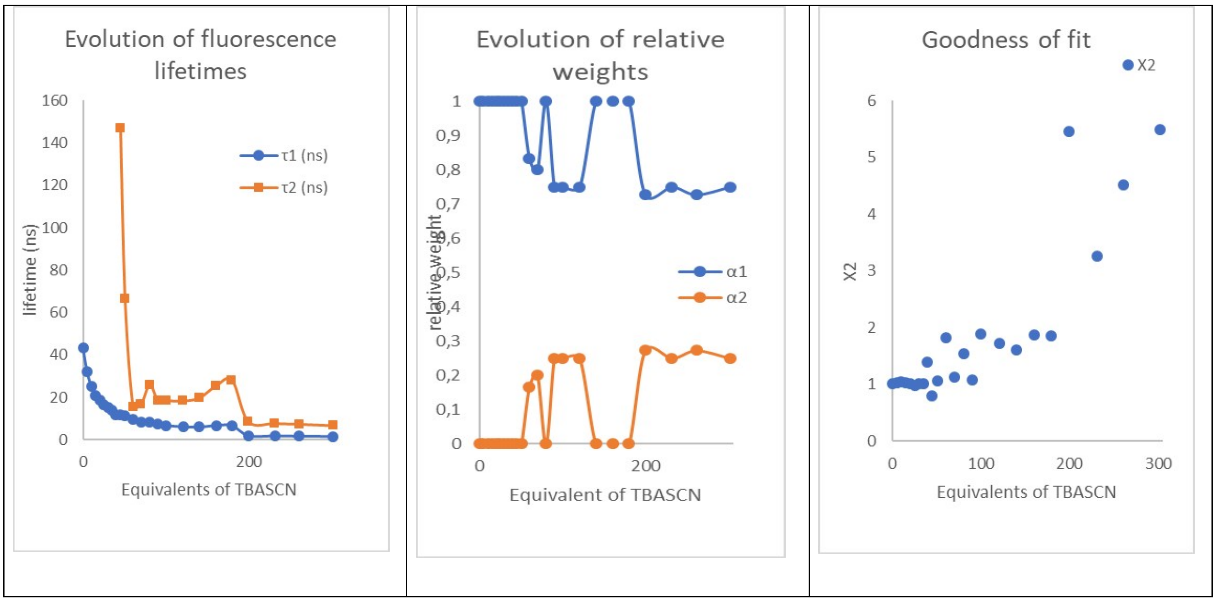

*Figure S60: Analysis of fluorescence decay titration of **2** with tetrabutylammonium thiocyanate*

7. Comparison of 1 and 2

Data from **1** were extracted from our previous study.[5]

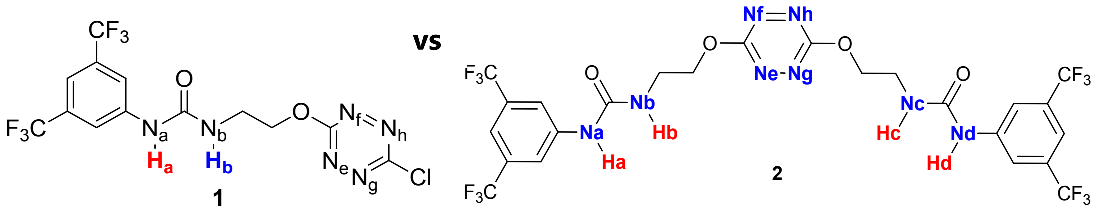

7.1 Geometrical parameters

Entry	Bond[a]	**1**	**1**-Cl	**1**-Br	**1**-I	**2**	**2**-Cl	**2**-Br	**2**-I
1	N_a-H_a	1.011	1.037	1.034	1.029	1.009	1.026	1.026	1.023
2	N_b-H_b	1.011	1.030	1.027	1.023	1.009	1.023	1.022	1.020
3	N_c-H_c	---	---	---	---	1.008	1.022	1.020	1.020
4	N_d-H_d	---	---	---	---	1.010	1.027	1.027	1.026
5	H_a-X	---	2.099	2.217	2.545	---	2.190	2.343	2.617
6	H_b-X	---	2.207	2.399	2.751	---	2.256	2.440	2.702
7	H_c-X	---	---	---	---	---	2.243	2.464	2.745
8	H_d-X	---	---	---	---	---	2.223	2.310	2.533

9	X-centroid	---	3.12	3.15	3.49	---	3.313	3.421	3.785
10	N_e-X	---	3.491	3.515	3.774	---	3.494	3.761	4.109
11	N_f-X	---	3.500	3.579	3.722	---	3.664	3.753	3.877
12	N_g-X	---	3.411	3.393	3.874	---	3.561	3.851	4.223
13	N_h-X	---	3.403	3.436	3.822	---	3.736	3.854	4.017

[a] distances in Å

*Figure S61: Geometrical parameters of receptors **1** and **2***

7.2 NMR shifts

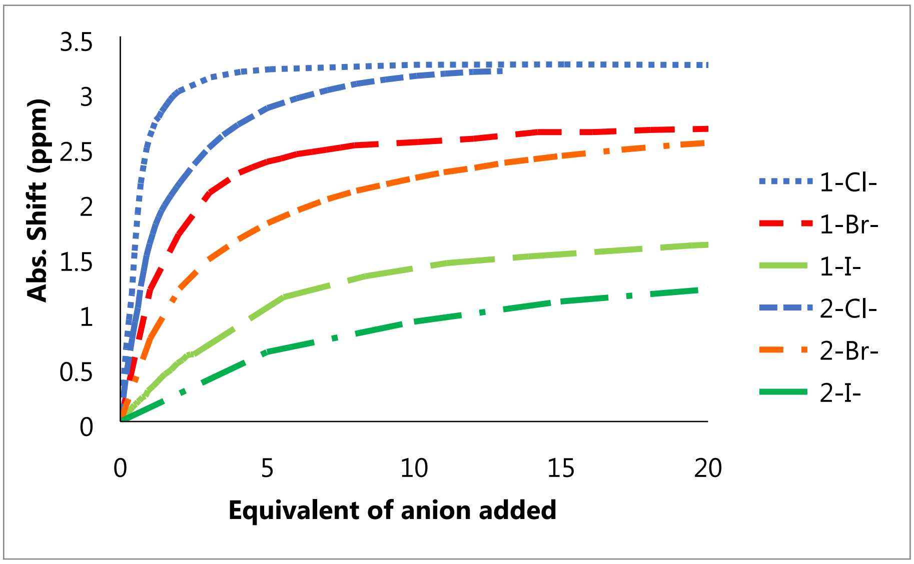

*Figure S62: Absolute NMR shifts for H_a **1** and **2***

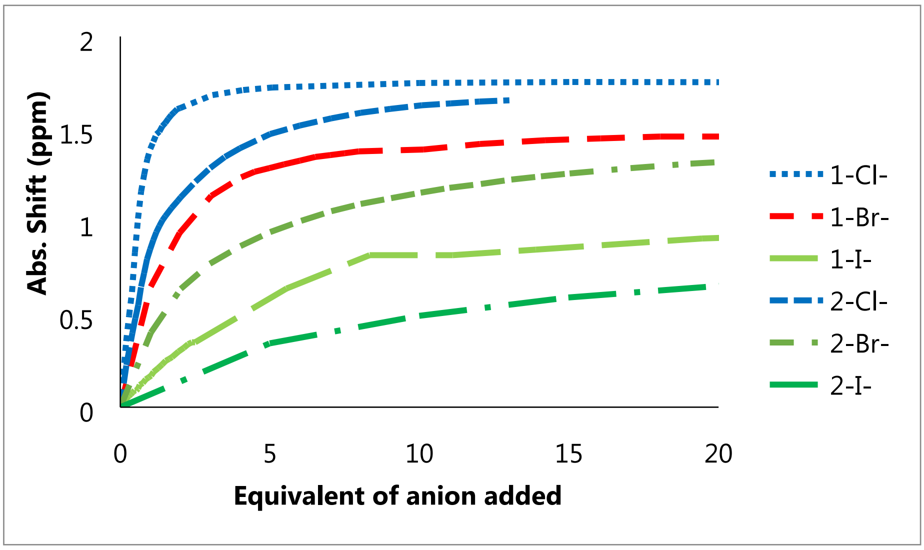

*Figure S63: Absolute NMR shifts for H_b **1** and **2***

7.3 Association constants

Entry		$K_{A, Ha/Hd}$[a]	$K_{A, Hb/Hc}$[a]	$K_{A, Hg/Hh}$[a]	$K_{A, SPECFIT}$[a]
1	**1**-Cl	5893	5700	5000	4892 ± 1 (0.6%)
2	**2**-Cl	459	487	61	424 ± 1 (0.4%)
3	**1**-Br	375	371	452	375 ± 1 (0.1%)
4	**2**-Br	114	116	93	296 ± 1 (1.3%)
5	**1**-I	64	67	77	70 ± 1 (0.2%)
6	**2**-I	21	22	33	20 ± 1 (0.2%)
7	**1**-SCN	37	37	59	37 ± 1 (0.4%)
8	**2**-SCN	11	12	17	11 ± 1 (0.1%)
9	**1**-PF_6	--[b]	--[b]	--[b]	--[b]
10	**2**-PF_6	--[b]	--[b]	--[b]	--[b]

[a] Binding constants in L/mol [b] No binding was observed

*Figure S64: Association constants measured by NMR titrations of **1** and **2** with different anions*

Entry		$K_{A, abs, SPECFIT}$[a]	$K_{A, fluo}$[a]	$K_{A, fluo, SPECFIT}$[a]	$K_{A, decay}$[a]
1	**1**-Cl	108 ± 1 (10%)	1763	1875 ± 1 (1%)	1461
2	**2**-Cl	11190 ± 1 (1%)	27141	27886 ± 1 (1%)	30000
3	**1**-Br	810 ± 1 (11%)	1176	1217 ± 1 (3%)	478
4	**2**-Br	196 ± 1 (2.1%)	1325	1267 ± 1 (1.0%)	1300
5	**1**-I	980 ± 1 (14%)	1479	1390 ± 1 (1%)	532
6	**2**-I	8 ± 1 (0.5%)	564	514 ± 1 (1.7%)	237
7	**1**-SCN	26 ± 1 (1%)	1386	1341 ± 1 (1%)	163

8	**2**-SCN	18 ± 1 (0.6%)	491	499 ± 1 (2.5%)	899
9	**1**-PF_6	--[b]	--[b]	--[b]	--[b]
10	**2**-PF_6	--[b]	--[b]	--[b]	--[b]

[a] Binding constants in L/mol [b] No binding was observed

*Figure S65: Association constants measured by photophysical titrations of **1** and **2** with different anions*

8. Bibliography

1 H. E. Gottlieb, V. Kotlyar and A. Nudelman, NMR Chemical Shifts of Common Laboratory Solvents as Trace Impurities, *J. Org. Chem.*, 1997, **62**, 7512–7515.

2 J. Contreras-García, E. R. Johnson, S. Keinan, R. Chaudret, J. P. Piquemal, D. N. Beratan and W. Yang, NCIPLOT: A Program for Plotting Noncovalent Interaction Regions, *J. Chem. Theory Comput.*, 2011, **7**, 625–632.

3 Henry S. Rzepa, Script for creating an NCI surface as a JVXL compressed file from a (Gaussian) cube of total electron density, https://www.ch.ic.ac.uk/rzepa/cub2nci/, (accessed 3 September 2019).

4 E. F. Pettersen, T. D. Goddard, C. C. Huang, G. S. Couch, D. M. Greenblatt, E. C. Meng and T. E. Ferrin, UCSF Chimera--a visualization system for exploratory research and analysis, *J. Comput. Chem.*, 2004, **25**, 1605–1612.

5 R. Plais, G. Gouarin, A. Gaucher, V. Haldys, A. Brosseau, G. Clavier, J.-Y. Salpin and D. Prim, Intertwined Detection and Recognition Roles of Tetrazine in Synergistic Anion-π and H-Bond Based Anion Receptor, *ChemPhysChem*, 2020, **21**, 1249–1257.

6 Y. H. Gong, P. Audebert, G. Clavier, F. Miomandre, J. Tang, S. Badré, R. Méallet-Renault and E. Naidus, Preparation and physicochemical studies of new multiple rings *s*-tetrazines, *New J. Chem.*, 2008, **32**, 1235–1242.

7 G. González-Gaitano and G. Tardajos, Chemical Equilibrium in Supramolecular Systems as Studied by NMR Spectrometry, *J. Chem. Educ.*, 2004, **81**, 270–274.

8 B. Valeur and M. N. Berberan-Santos, *Molecular Fluorescence: Principles and Applications, Second Edition*, Wiley-VCH, Weinheim, 2012.

9 H. Gampp, M. Maeder, C. J. Meyer and A. D. Zuberbühler, Calculation of equilibrium constants from multiwavelength spectroscopic data--I: mathematical considerations, *Talanta*, 1985, **32**, 95–101.

10 H. Gampp, M. Maeder, C. J. Meyer and A. D. Zuberbühler, Calculation of equilibrium constants from multiwavelength spectroscopic data--II: SPECFIT: two user-friendly programs in basic and standard FORTRAN 77, *Talanta*, 1985, **32**, 257–264.

11 C. Würth, M. Grabolle, J. Pauli, M. Spieles and U. Resch-Genger, Relative and absolute determination of fluorescence quantum yields of transparent samples, *Nat. Protoc.*, 2013, **8**, 1535–1550.

12 A. M. Brouwer, Standards for photoluminescence quantum yield measurements in solution (IUPAC Technical Report), *Pure Appl. Chem.*, 2011, **83**, 2213–2228.
